# Evolution of Rosaceae Plastomes Highlights Unique *Cerasus* Diversification and Independent Origins of Fruiting Cherry

**DOI:** 10.3389/fpls.2021.736053

**Published:** 2021-11-19

**Authors:** Jing Zhang, Yan Wang, Tao Chen, Qing Chen, Lei Wang, Zhen-shan Liu, Hao Wang, Rui Xie, Wen He, Ming Li, Cong-li Liu, Shao-feng Yang, Meng-yao Li, Yuan-xiu Lin, Yun-ting Zhang, Yong Zhang, Ya Luo, Hao-ru Tang, Li-zhi Gao, Xiao-rong Wang

**Affiliations:** ^1^College of Horticulture, Sichuan Agricultural University, Chengdu, China; ^2^Institute of Pomology and Olericulture, Sichuan Agricultural University, Chengdu, China; ^3^College of Life Science, Sichuan Agricultural University, Ya’an, China; ^4^Zhengzhou Fruit Research Institute, Chinese Academy of Agricultural Sciences, Zhengzhou, China; ^5^Institute of Genomics and Bioinformatics, South China Agricultural University, Guangzhou, China; ^6^Plant Germplasm and Genomics Center, Germplasm Bank of Wild Species in Southwest China, Kunming Institute of Botany, Chinese Academy of Sciences, Kunming, China

**Keywords:** *Cerasus*, evolutionary pattern, fruiting cherry, genomic variation, plastomes, Rosaceae

## Abstract

Rosaceae comprises numerous types of economically important fruits, ornamentals, and timber. The lack of plastome characteristics has blocked our understanding of the evolution of plastome and plastid genes of Rosaceae crops. Using comparative genomics and phylogenomics, we analyzed 121 Rosaceae plastomes of 54 taxa from 13 genera, predominantly including *Cerasus* (true cherry) and its relatives. To our knowledge, we generated the first comprehensive map of genomic variation across Rosaceae plastomes. Contraction/expansion of inverted repeat regions and sequence losses of the two single-copy regions underlie large genomic variations in size among Rosaceae plastomes. Plastid protein-coding genes were characterized with a high proportion (over 50%) of synonymous variants and insertion-deletions with multiple triplets. Five photosynthesis-related genes were specially selected in perennial woody trees. Comparative genomic analyses implied divergent evolutionary patterns between pomaceous and drupaceous trees. Across all examined plastomes, unique and divergent evolution was detected in *Cerasus* plastomes. Phylogenomic analyses and molecular dating highlighted the relatively distant phylogenetic relationship between *Cerasus* and relatives (*Microcerasus*, *Amygdalus*, *Prunus*, and *Armeniaca*), which strongly supported treating the monophyletic true cherry group as a separate genus excluding dwarf cherry. High genetic differentiation and distinct phylogenetic relationships implied independent origins and domestication between fruiting cherries, particularly between *Prunus pseudocerasus* (*Cerasus pseudocerasus*) and *P. avium* (*C. avium*). Well-resolved maternal phylogeny suggested that cultivated *P. pseudocerasus* originated from Longmenshan Fault zone, the eastern edge of Himalaya-Hengduan Mountains, where it was subjected to frequent genomic introgression between its presumed wild ancestors and relatives.

## Introduction

Rosaceae, comprising over 3,000 species, is the third economically important family in temperate region with many famous fruit, ornamental, and timber crops (Yü et al., 1986; Shulaev et al., 2008; Hummer and Janick, 2009; Phipps, 2014). This family has a wide distribution in warm temperate and subtropical regions of the Northern Hemisphere (Yü, 1984; Potter et al., 2007; Shulaev et al., 2008; Zhang et al., 2017). It covers over 80% of deciduous fruit species in temperate regions (Yü, 1984). Rosaceae fruit crops, with production of over one million tons, include apple, peach, pear, plum, strawberry, cherry, and apricot (FAO statistics in 2018)^1^, six of which are the perennial pomaceous and drupaceous woody fruit trees in the two tribes, Maleae (Pyreae) and Amygdaleae, of the Amygdaloideae (the former Spiraeoideae) subfamily (Potter et al., 2007; McNeill et al., 2012). Many other important woody fruit trees, such as quince, loquat, and fruiting mei; famous perennial woody ornamentals, such as flowering cherry and mei (*Prunus mume*); and timber, such as cherrywood (*Prunus serotina*), also belong to the two tribes. Especially, perennial woody fruit trees belonging to the two tribes are also of economical importance in ornamental and timber. Thus, the tribes Maleae and Amygdaleae of the Amygdaloideae subfamily represent economically important groups in the Rosaceae family.

*Cerasus* plants are one of the most representative economically important groups in the Rosaceae family, and include many fruit trees (*P. pseudocerasus*, *Prunus avium*, *Prunus tomentosa*, *Prunus cerasus*, fruiting cherry) and ornamentals (*Prunus yedoensis*, *Prunus serrulata*, *Prunus campanulata*, *Prunus cerasoides*, flowering cherry). It consists of ∼150 species, which account for over one-third of the total number of the tribe Amygdaleae species (∼400) (Yü et al., 1986). *Cerasus* plants are naturally distributed in temperate Asia, Europe, and North America, and one of their diversity centers is thought to be Southwest China, in which *Cerasus* plants have wide and overlapping distributions along the Qinling Mountains (QLM), Himalaya-Hengduan Mountains (HHM), and Yun-Gui Plateau (YGP) (Chen et al., 2020). Generally, *Cerasus* plants consist of true cherry (*Cerasus*) and dwarf cherry (*Microcerasus*). Some plant taxonomists and horticulturalists treated *Cerasus* as a separate genus based on morphology, and isozyme and molecular markers (de Tournefort, 1700; Linnaeus, 1754; Bate-Smith, 1961; Komarov, 1971; Shishkin and Yuzepchuk, 1971; Yü et al., 1986; Takhtajan, 1997), while other scholars merged *Cerasus* into the broad-sensed *Prunus* genus as one of its subgenera (Bentham and Hooker, 1865; Focke, 1894; Schneider, 1905; Koehne, 1911; Rehder, 1940; Ingram, 1948; Hutchinson, 1964; Krüssmann, 1978; Ghora and Panigrahi, 1995; Supplementary Table 1). The classification of dwarf cherry also varies among taxonomists (Supplementary Table 1). Recent molecular studies have provided valuable insights into the phylogeny and geographical origin of true cherry, dwarf cherry, and relatives (Shi et al., 2013; Chin et al., 2014; Zhao et al., 2016). Nevertheless, the taxonomy of *Cerasus* remains unresolved.

Chinese cherry (*P. pseudocerasus*/*C. pseudocerasus*, 2*n* = 4*x* = 32, Wang et al., 2018) and European sweet cherry (*P. avium*/*C. avium*, 2*n* = 2*x* = 16, Wang et al., 2018) are two economically important fruiting cherry species, and they have largely contributed to the poverty alleviation and rural vitalization of China. Chinese cherry, native to China, is characterized by full-flavored but small size fruits, and European sweet cherry produces large-sized fruits with excellent shipping quality but exhibits narrow ecological adaptation. European sweet cherry was first introduced to China in the 1870s, and then it has been widely cultivated since 1990s. In China, European sweet cherry shows excellent performance in suitability, productivity, and fruit quality when it is cultivated in the Southwest China where wild *Cerasus* plants are widely distributed, while Chinese cherry exhibits intensive disease/pest resistance and excellent adaptation to diverse ecological environments (Yü, 1979; Huang et al., 2013; Chen et al., 2016a, 2020). Thus, to effectively utilize their advantageous traits in further cherry breeding and genetic improvement, it is quite necessary to investigate the genetic relationship, as well as the origin and domestication history, of the two cultivated cherry species. However, the detailed geographical origin and dispersal routes of Chinese cherry (*P. pseudocerasus*) still need direct cytoplasmic genome data. The phylogenetic relationship between the two cultivated cherry species, *P. pseudocerasus* and *P. avium*, also remains unknown.

Rosaceae crops are often characterized by complex genome compositions and diverse parental contributions (Yamamoto and Terakami, 2016; Aranzana et al., 2019; Chen et al., 2019), which leads to a huge challenge in exploring their evolutionary history. Recently, plastomes data have played increasingly important roles in revealing the origin and domestication of fruit crops with complex genetic backgrounds due to their maternal transmission, small genome size, and low substitution rate (Carbonell-Caballero et al., 2015; Aubriot et al., 2018; Li et al., 2020; Sudianto et al., 2020). Meantime, the information on single nucleotide polymorphism (SNP), insertion-deletions (InDels), and simple sequence repeats (SSRs) have also enhanced our understanding of the evolutionary patterns and mechanisms of the maintenance or disruption of plant plastomes (Gao et al., 2019). Rapid development of the next-generation sequencing techniques nowadays allows us to conduct comparative genomic and phylogenomic analyses with a large sample size. Although expansion/contraction of inverted repeats (IRs) and rich genomic variations have been reported in Rosaceae plastomes (Terakami et al., 2012; Wang et al., 2013; Xue et al., 2019), how these genomic variations trigger the evolution of plastomes and plastid genes still remains unclear.

Herein, we reported 91 newly assembled plastomes of *Cerasus* and its relatives. Combined with publicly available plastomes, we analyzed 124 plastomes that represented 54 taxa from 13 genera from the two subfamilies of family Rosaceae and three species from three other families in Rosales, predominantly including the *Cerasus* and its relatives. By comparative genomic and phylogenomic analyses, our aims are (i) to investigate and compare the evolutionary patterns of Rosaceae plastomes and plastid genes by selecting economically important Rosaceae crops mainly from tribes Amygdaleae and Maleae of subfamily Amygdaloideae, (ii) to better solve the taxonomic status of *Cerasus* referring to family- and subfamily-, and tribe-level analyses, and (iii) to clarify the origin of fruiting cherry species with particular emphasis on cultivated *P. pseudocerasus*/*C. pseudocerasus*.

## Materials and Methods

### Plant Materials

A total of 124 plastomes were preliminary selected, among which 91 were newly assembled and 33 were downloaded from the National Center for Biotechnology Information (NCBI) database (Supplementary Table 2). Our samples covered 13 genera of subfamilies Amygdaloideae (tribes Amygdaleae, Exochordeae, Spiraeeae, and Maleae) and Rosoideae of family Rosaceae. Three species, *Morus mongolica* (Moraceae), *Ziziphus jujuba* (Rhamnaceae), and *Elaeagnus macrophylla* (Elaeagnaceae), were used as out-groups. Based on our previous studies (Huang et al., 2013; Chen et al., 2015, 2016a,b, 2020; Liu et al., 2016; Zhang et al., 2018), 90 representative true cherry (*Cerasus*) and dwarf cherry (*Microcerasus*) accessions were selected for whole-genome re-sequencing, consisting of 34 *P. pseudocerasus* accessions (11 landraces and 23 wild types) representing diverse genotypes, phenotypes and geographical distributions, 6 accessions referring to 4 European cherry taxa [*P. avium*, *Prunus fruticosa*, *P. cerasus* × *Prunus canescens* (Gisela 5), and *Prunus mahaleb*], 46 accessions covering 20 other *Cerasus* taxa, and 4 dwarf cherry accessions (*P. tomentosa* and *P. tianshanica*). We also obtained the genomic pair-end reads of *Prunus cerasifera* (SRR4036106) from the GenBank database to assemble its plastome.

### Genomic DNA Extraction, Sequencing, and Plastome Assembly

Total genomic DNA was extracted from silica-gel dried leaf tissues following the modified cetyltrimethyl ammonium bromide (CTAB) method used by Chen et al. (2013). Ninety-one Illumina paired-end (PE) libraries with 500-bp insert size were constructed and sequenced using an Illumina HiSeq 2000 (Illumina, San Diego, CA, United States) instrument by BGI-Shenzhen (Shenzhen, China). Taking *Prunus persica* (Jansen et al., 2011) and *P. pseudocerasus* (Feng et al., 2017) as reference plastomes, we obtained plastid reads for these 91 accessions. These reads were then assembled into contigs and scaffolds using SPAdes v.3.9.0 (Bankevich et al., 2012). The scaffolds were aligned to the reference plastomes of *P. persica* in Geneious v.8.1 (Kearse et al., 2012) and then were manually ordered as the genomes in the SnapGene v.2.3.2 software^2^. The newly assembled plastomes were deposited in the GenBank database under the following accession numbers: MT576845-MT576934 (Supplementary Table 2).

### Gene Annotation and Visualization

Gene annotation was conducted in GeSeq^3^ (Tillich et al., 2017). All ambiguous annotations, such as the absence of start/stop codons, were manually corrected in SnapGene, referring to the downloaded 33 Rosaceae plastomes. Genome structures were drawn with Circos v.0.69.6 (Krzywinski et al., 2009).

### Genomic Variation Analyses

The plastome sequences were aligned in MAFFT v.7.037b (Katoh and Standley, 2013). Nucleotide contents and coefficients of sequence similarities were calculated with BioEdit v.7.0.5 (Hall, 1999). Genetic distances were calculated with Tajima-Nei model in MEGA v.5.1 (Tamura et al., 2011). Genetic differentiation coefficient (*F*_ST_ value) was estimated using DnaSP v.6 (Rozas et al., 2017).

Microsatellites in each plastome were screened with MISA perl script^4^ with the following parameters: mononucleotide SSR repeat units ≥ 10, dinucleotide repeat units ≥ 6, trinucleotide repeat units ≥ 5, tetranucleotide repeat units ≥ 4, and pentanucleotide and hexanucleotide repeat units ≥ 3.

Taking *P. pseudocerasus* (NC030599.1) as the reference plastome, we employed MUMmer v.3.3 (Kurtz et al., 2004) to identify SNP and InDels for each Rosaceae plastome. Independent SNP and InDel files from different individuals were transformed and combined into one SNP and InDel vcf file with BCFTools v.1.7. BEDTools v.2.26.0 (Quinlan and Hall, 2010) and SnpEff_latest_core^5^ were used to detect the distributions of SSRs, SNPs, and InDels across the plastomes and estimate the effects of SNPs and InDels on gene functions.

To calculate the proportion of mutational events (Gielly and Taberlet, 1994; Huang et al., 2014), we also detected SNPs and InDels between pairwise plastomes in MUMmer v.3.3. The proportion of mutation events (PME) was calculated as [(NS + NI)/LA] × 100, where NS represented the number of nucleotide substitution between plastomes, NI represented the number of InDels, and LA denoted the length of the aligned plastome sequences.

We employed site models and likelihood ratio test (LRT) implemented in PAML v.4.9h (Yang, 2007) to detect the signatures of positive selection for 81 unique plastid protein-coding genes. At the Rosaceae level, we removed *rps19*-fragment (not completely assembled in some taxa), *rps12* (the special gene structure), and *infA* (pseudogene) from this analysis. Except for the three genes, 43 highly conserved plastid protein-coding genes have also been excluded for true cherries. Seventy-eight and 35 protein-coding genes remained for Rosaceae taxa and true cherries, respectively. First, selective pressures were computed in CodeML (included in PAML package) with three site models: M0 (model = 0, NS sites = 0), M1a (model = 0, NS sites = 1), and M2a (model = 0, NS sites = 1) (Nielsen and Yang, 1998). Then, likelihood ratio test was conducted to compare M1a against M2a by calculating the χ^2^ critical value and *P* value. Finally, when the log likelihoods between the two models were statistically different (*P* < 0.05 in LRT), positively selected sites of genes were identified by Bayes empirical Bayes (BEB) analysis (posterior probabilities for site class > 0.95 and ϖ > 1) (Zhang et al., 2005) in the CodeML program. In addition, a branch-site model (Yang et al., 2005; Yang, 2007) was also used to investigate branch-specific selection for true cherries. Likelihood ratio test for positive selection on each examined branch was compared model A (model = 2, NS sites = 2, fix_omega = 0, omega = 1.5) against null model (model = 2, NS sites = 2, fix_omega = 1, omega = 1). Also, positively selected sites were determined by LRT and BEB analyses.

### Phylogenetic Analysis and Molecular Dating

Systematic errors are thought to mainly result from the inaccurate alignment caused by rapidly evolving sites and may lead to an incorrect tree with strong supports, while the removal of problematic regions is an effective method for improving the robustness of phylogenomic reconstruction (Rodríguez-Ezpeleta et al., 2007). To reduce potential systematic errors, we constructed 12 different datasets to carry out the phylogenomic analyses at the Rosaceae family level and at the tribe Amygdaleae level. Since gene order and content were highly conserved in the studied plastomes of Rosaceae and the three outgroups, the alignment could be straightforward. The 12 datasets were as follows: (i) WCGD (whole plastomes dataset, *n* = 124) and PCGD (Amygdaleae plastomes dataset, *n* = 107) were constructed with complete plastome sequences, both removing all missing data (N) and long insertion (> 50 bp) sequences that were only detected in an individual; (ii) WOID (whole one inverted-repeat dataset, *n* = 124) and POID (Amygdaleae one inverted-repeat dataset, *n* = 107) were generated using large single-copy (LSC), short single-copy (SSC), and one IR sequences, also both removing all missing data and long insertion (> 50 bp) sequences; (iii) VSWD (variant sites of whole plastomes dataset, *n* = 124) and VSPD (variant sites of Amygdaleae plastomes dataset, *n* = 107) were constructed with the variant sites (SNPs) of WCGD and PCGD using the custom bash script, respectively; (iv) WGSD (whole gene sequence dataset, *n* = 124) and PGSD (Amygdaleae gene sequence dataset, *n* = 107) contained sequences of 102 unique genes; (v) PCWD (protein-coding sequence of whole plastomes dataset, *n* = 124) and PCPD (protein-coding sequence of Amygdaleae plastomes dataset, *n* = 107) consisted of exon sequences of 72 unique protein-coding genes; and (vi) PWGD (pruned whole plastomes dataset, *n* = 124) and PPGD (pruned Amygdaleae plastomes dataset, *n* = 107) were generated by removing rapidly evolving sites, and large InDels and sequences with rich structural variation of WCGD and PCGD in GBlocks v.0.91b (Castresana, 2000) (parameters: minimum sequences per conserved position, 65; minimum sequences per flank position, 110 (PWGD)/100 (PPGD); maximum number of contiguous non-conserved positions, 8; minimum block length, 10; allowed gap positions, none).

We employed Maximum likelihood (ML) methods to generate phylogenetic trees for each dataset mentioned above. For each dataset, the best-fit model of nucleotide substitution was selected with jModelTest v.2.1.7 (Darriba et al., 2012) using the Akaike Information Criterion (AIC). Bayesian inference (BI) analyses were conducted with MrBayes v.3.2.6 (Ronquist et al., 2012). Two independent Markov chain Monte Carlo (MCMC) algorithm chains were carried out, and each of them ran with one cold and three heated chains for 12,000,000 generations and started with a random tree and sampling one tree every 100 generations. When the average standard deviation of split frequencies was below0.01 between the two runs, analyses were considered to reach stationarity. The first 25% of generations were treated as burn-in. ML analyses were performed using IQ-TREE v.1.5.5 (Nguyen et al., 2015) with 1,000 regular bootstrap replicates (-b 1,000). All the phylogenetic trees were edited and presented using iTOL v.5 (Letunic and Bork, 2021) and FigTree v.1.4.4.^6^

Divergence time was estimated with BEAST v.2.6.6 (Bouckaert et al., 2019). Since 8 (WCGD, WOID, PWGD, VSWD, PCGD, POID, PPGD, and VSPD) of the 12 datasets produced congruent topologies with high statistical supports, we used the PWGD in molecular dating following the method of Zhang et al. (2017). To further decrease computation power requirement, with the exception of *P. pseudocerasus* and *P. avium*, we kept only one accession for each taxon within inter nodes to construct a pruned PWGD. Software parameters were finally set as the GTR substitution model and exponential uncorrelated relaxed model with Yule process. Two independent MCMC runs were conducted, each with 300,000,000 generations and sampling every 1,000 generations. The first 12,000,000 generations in each run were removed as burn-in. The fossil *P. wutuensis* from Wutu Formation, Shandong province, China, has been dated to 47.8–55 Mya in Early Eocene (Li et al., 2011), and age estimates of *Prunus* was from 60.7 to 62.4 Mya (Chin et al., 2014; Table 1). Therefore, the age of crown *Prunus* (N1) was constrained by a log-normal distribution with a mean of 55 Mya and a standard deviation of 0.09 in our study. The divergence time of the tribe Maleae (only containing *Malus*, *Pyrus*, *Chaenomeles*, *Cydonia*, *Docynia*, *Eriobotrya*, and *Sorbus*) was estimated at approximately 42 Mya in recent molecular study (Xiang et al., 2017). Leaf fossils distinguishing *Malus* from *Pyrus* has been dated to 45 Mya (Wehr and Hopkins, 1994). Here, we set the age of crown Maleae (N2) as a log-normal distribution with a mean value of 45 Mya and a standard deviation of 0.01. Based on mesofossil (Edelman, 1975), ages of crown Rosoideae (only containing *Rosa*, *Fragaria*, and *Potentilla*) (N3) were constrained by the log-normal distribution with a mean value of 48.6 and standard deviation of 0.05. The recent age estimate of the divergence between Rosaceae and other Rosales taxa was at 106.5 Mya (Zhang et al., 2017). Thus, the crown Rosales (N4) was constrained by log-normal distribution with a mean value of 106.5 Mya and standard deviation of 0.05. Tree files and log files from the two independent runs were combined with LogCombiner v.2.6.6 (part of the BEAST package). The effective sample size (ESS) for each logged statistic was estimated in Tracer v.1.7.2 (Rambaut et al., 2018), and most of the ESSs were above 200. Finally, the consensus tree and divergence time were calculated and annotated in the TreeAnnotator v.2.6.6 (part of the BEAST package).

**TABLE 1:** Fossils and molecular estimation used as calibration points for molecular dating.

**Node**	**Anchor fossil**	**Molecular estimation**	**Fossil assigned date, Myr/molecular estimation time, Myr**	**Mean values/standard deviation used at calibration points**	**References**
N1	*Prunus wutuensis*	Node *Prunus*	47.8–55/60.7–62.4	55.0/0.09	Li et al., 2011; Chin et al., 2014
N2	*Malus*, *Pyrus*	Tribe Maleae^a^	45/∼42	45.0/0.01	Wehr and Hopkins, 1994; Xiang et al., 2017
N3	*Rosa germerensis*	–	48.6/–	48.6/0.05	Edelman, 1975
N4	–	Rosales	–/106.5	106.5/0.05	Zhang et al., 2017

*^*a*^In this study, the Maleae tribe only included Malus, Pyrus, Chaenomeles, Cydonia, Docynia, Eriobotrya, and Sorbus. –, not available.*

## Results

### Assembly and Characterization of Rosaceae Plastomes

Ninety-one new plastomes of 27 *Cerasus* and *Microcerasus* taxa (25 species and two varieties) and one closely related species (*P. cerasifera*) were assembled (Supplementary Table 3). Mean coverage of these newly assembled plastomes ranged from 171 (*P. pseudocerasus*) to 9,065 × (*P. cerasifera*) (Supplementary Table 3). Thirty previously published plastomes of Rosaceae were also downloaded. The overall Guanine-Cytosine (GC) content of the 121 Rosaceae plastomes was ∼37% (Supplementary Table 4).

**FIGURE 1:**
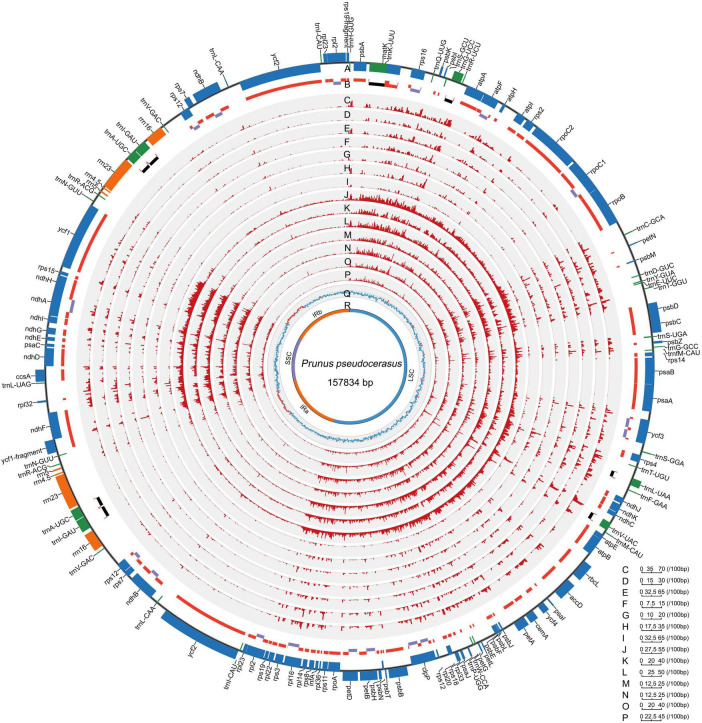
Landscape of genomic variations across the 121 Rosaceae plastomes. A. Gene distribution (blue, protein-coding genes; green, tRNAs; orange, rRNAs). B. Exons and introns (red, exons in protein-coding genes; black, exons in tRNAs; purple, introns). C-I/J-P. Density of insertion-deletion (InDel)/single nucleotide polymorphism (SNP) in plastomes of the Rosaceae family, Rosoideae and Amygdaloideae subfamilies, and Spiraeeae, Exochordeae, Maleae, and Amygdaleae tribes (100-bp window). Q/R. GC contents/distributions of large single-copy (LSC), short single-copy (SSC) and two inverted repeats (IRs) (100-bp window; light blue, GC content over 50%; orange, GC content below 50%). *Prunus pseudocerasus* (*Cerasus pseudocerasus*) (NC030599.1) was used as reference genome.

All the 121 examined Rosaceae plastomes exhibited typical genomic structures, consisting of one LSC and SSC, and two conserved inverted repeats (IRa and IRb) (Figure 1). They also possessed conserved gene orders and gene contents with 132 identified genes (115 unique genes), namely, 87 (81 unique) protein-coding genes, and 37 tRNA and 8 rRNA coding genes (Figure 1, Table 2, and Supplementary Table 4). Among these identified genes, nine protein-coding and five tDNA genes contained one intron, and three protein-coding and one tDNA genes contained more than one intron (Table 2). Most of the plastid genes were linearly concentrated on the plastomes (Figure 1), while the overlapping genomic regions that were detected between *matK* and *trnU-UUU*, between *ycf1*-fragment and *ndhF*, and between *psbC* and *psbD*. *MatK* were completely nested in the intron region of *trnU-UUU* in all the examined plastomes. The overlapping genomic region between *ycf1*-fragment and *ndhF* was observed in most plastomes of subfamily Amygdaloideae, with 21-bp length in the tribe Maleae and 4–173-bp length in the tribe Amygdaleae (Supplementary Table 4). A conserved overlapping genomic region (53-bp size) was detected between *psbC* and *psbD* in all the Rosaceae plastomes and three other Rosales plastomes (Supplementary Table 4).

**TABLE 2:** List of the 115 identified unique genes of Rosaceae plastomes.

**Classification**	**Gene category**	**Genes**
Self-replication	Ribosomal RNAS	*^*I*^rrn4.5 _(__2__)_, ^*I*^rrn5_ (__2__)_, ^*I*^rrn16 _(__2__)_, ^*I*^rrn23_ (__2__)_*
	Transfer RNAs	^I,*,§^ trnA-UGC _(__2__)_, ^*L*^trnC-GCA, ^*L*^trnD-GUC, ^*L*^trnE-UUC, ^*L*^trnF-GAA, ^L,*^trnfM-CAU, ^*L*^trnG-GCC, ^*L,§*^ trnG-UCC, ^*L*^trnH-GUG, ^I,*^trnI-CAU_ (__2__)_, ^I,*,§^ trnI-GAU_ (__2__)_, ^*L,§*^ trnK-UUU, ^I,*^trnL-CAA_ (__2__)_, ^*L,§*^ trnL-UAA, ^S^trnL-UAG, ^*L*^trnM-CAU, ^I,*^trnN-GUU_ (__2__)_, ^*L*^trnP-UGG, ^*L*^trnQ-UUG, ^I,*^trnR-ACG_ (__2__)_, ^*L*^trnR-UCU, ^*L*^trnS-GCU, ^*L*^trnS-UGA, ^*L*^trnS-GGA, ^*L*^trnT-UGU, ^*L*^trnT-GGU, ^*L,§*^ trnV-UAC, ^*I*^trnV-GAC_ (__2__)_, ^*L*^trnW-CCA, ^*L*^trnY-GUA
	Ribosomal protein (large subunit)	^I,*,§^ rpl2_ (__2__)_, ^L,*^rpl14, ^L,*,§^ rpl16, ^L,*^rpl20, ^L,*^rpl22, ^I,*^rpl23 _(__2__)_, ^S,*^rpl32, ^L,*^rpl33, ^L,*^rpl36
	Ribosomal protein (small subunit)	^L,*^rps2, ^L,*^rps3,^ L,*^rps4, ^I,*^rps7_ (__2__)_, ^L,*^rps8, ^L,*^rps11, ^S,L,I,*,§^ rps12_ (__2__)_, ^L,*^rps14, ^S,*^rps15, ^L,*,§^ rps16, ^L,*^rps18, ^L,I,*^rps19, ^L,I,*^rps19-fragment
	RNA polymerase	^L,*^rpoA, ^L,*^rpoB, ^L,*,§^ rpoC1, ^L,*^rpoC2
Photosynthesis	Photosystem I	^L,*^psaA, ^L,*^psaB, ^S,*^psaC, ^L,*^psaI, ^L,*^psaJ, ^L,*^ycf4
	Photosystem II	^L,*^psbA, ^L,*^psbB, ^L,*^psbC, ^L,*^psbD, ^L,*^psbE, ^L,*^psbF, ^L,*^psbH, ^L,*^psbI, ^L,*^psbJ, ^L,*^psbK, ^L,*^psbL, ^L,*^psbM, ^L,*^psbN, ^L,*^psbT, ^L,*^psbZ
	Cytochrome	^L,*^petA, ^L,*,§^ petB, ^L,*,§^ petD, ^L,*^petG, ^L,*^petL, ^L,*^petN
	ATP synthase	^L,*^atpA, ^L,*^atpB, ^L,*^atpE, ^L,*,§^ atpF, ^L,*^atpH, ^L,*^atpI
	Rubisco	^L,*^rbcL
	NADH dehydrogenase	^S,*,§^ ndhA, ^I,*,§^ ndhB_ (__2__)_, ^L,*^ndhC, ^S,*^ndhD, ^S,*^ndhE, ^S,I,*^ndhF, ^S,*^ndhG, ^S,*^ndhH, ^S,*^ndhI, ^L,*^ndhJ, ^L,*^ndhK
Other genes	Other proteins	^L,*^accD
	Protease	^L,*,§^ clpP
	Cytochrome c biogenesis	^S,*^ccsA
	Membrane protein	^L,*^cemA
	Maturase	^L,*^matK
Unknown function	Conserved reading frames	^S,I,*^ycf1-fragment, ^S,I,*^ycf1, ^I,*^ycf2_ (__2__)_, ^L,*,§^ ycf3, ^*L,X*^infA

A total of 132 plastid genes were detected, among which 115 were unique. LSC (long single-copy) contained 22 transfer ribonucleic acid (tRNA) and 63 protein-coding genes, inverted repeats (RIs) included 14 tRNA, 17 protein-coding, and 8 ribosomal ribonucleic (rRNA) genes, and SSC (short single-copy) harbored one tRNA and 14 protein-coding genes; _(__2__)_: two copies in cp genomes; ^*x*^: pseudogene; ^*L*^: the gene distributed in LSC region; ^*S*^: the gene distributed in SSC region; ^*I*^: the gene distributed in IR region; *: protein-coding gene; ^§^ : the gene containing intron.

### Genome Variation Across Rosaceae Plastomes

#### Genome Size Variation Across Rosaceae Plastomes

The sizes of the examined Rosaceae plastomes highly varied and ranged from 154,959 to 160,390 bp (Supplementary Table 4). Plastome sizes of the three subfamily Rosoideae species (154,959–156,749 bp) was generally smaller than those of the subfamily Amygdaloideae taxa (156,328–160,390 bp) (Supplementary Table 4). In the subfamily Amygdaloideae, the largest plastome sizes (159,137 to 160,390 bp) were observed in the tribe Maleae, followed by tribes Amygdaleae (157,107–158,955 bp), Spiraeeae (156,612 bp), and Exochordeae (156,328 bp) (Supplementary Table 4). In comparison with the plastomes of the subfamily Amygdaloideae, those of subfamily Rosoideae exhibited ∼ 900-bp decreases in the LSC region mainly because of complete or partial losses of *rps19*-fragment and *atpF* genes (Supplementary Table 4), and they also showed 80- to 1,000-bp decreases in SSC length among 34 representative Rosaceae plastomes (Supplementary Table 5). For the subfamily Amygdaloideae plastomes, the sizes of the LSC (*r* = 0.978, *P* < 2.2e^–16^) and SSC regions (*r* = 0.716, *P* = 5.877e^–06^) were significantly and positively correlated to their whole plastome size, but that of IR is not (*r* = -0.099, *P* = 0.595).

Inverted repeat contraction and expansion were also investigated in the 34 representative Rosaceae plastomes (Supplementary Figure 1 and Supplementary Table 5). Significant IR contraction was observed in the plastomes of subfamily Rosoideae, with both *rps19* and *ndhF* locating out of the IRb boundary with at least 11- and 31-bp length (Supplementary Figure 1). In subfamily Amygdaloideae, the boundaries of IR and SC regions were highly various in tribe Amygdaleae plastomes but nearly identical in tribe Maleae plastomes (Supplementary Figure 1). Remarkably, we found that plastomes size variations of *Cerasus* and *Microcerasus* were more violent in LSC (838 bp), SSC (123 bp), and IR (94 bp) than other taxonomic groups, such as *Amygdalus* (129, 170, and 20 bp), *Malus* (83, 8, and 2 bp), and *Pyrus* (21, 4, and 2 bp) (Supplementary Table 5). Meanwhile, different from the conservation of IR and SC regions observed in the three taxonomic groups, a much more abundant genomic variation existed within the boundaries of the IR and SC regions in *Cerasus* and *Microcerasus* plastomes (Supplementary Figure 1).

#### Genome Structure Variation in Rosaceae Plastomes

To reveal the mutational hotspots for Rosaceae plastomes, we examined the distribution, number, and type of InDels and SNPs, and generated a map of genomic variation across the 121 Rosaceae plastomes (Figure 1 and Supplementary Table 6). In examined Rosaceae plastomes, most InDels and SNPs were conventionally distributed in intergenic and intronic regions (Figure 1 and Supplementary Table 6). A total of 6,745 InDel loci and 20,817 SNP loci were identified, with densities of 42.73 and 131.89 per kb, respectively (Supplementary Table 6). Among these, 1,439 InDel loci and 2,464 SNP loci were polymorphic (Supplementary Table 6). We observed more insertions than deletions at most of taxonomic levels of Rosaceae (Supplementary Table 6), and most of the InDels were fewer than 10 bp (Supplementary Figure 2A). The richest nucleotide substitution was the transition from C to T in most of the taxonomic groups (Supplementary Table 7), except for tribes Amygdaleae and Exochordeae, in which the reverse mutation from T to C was dominant. Interestingly, tribe Amygdaleae plastomes contained three types of richest nucleotide substitutions (G to T, T to C, and G to A), exhibiting more diverse patterns than that of tribe Maleae plastomes (Supplementary Table 7). Especially, *Cerasus* (true cherry) plastomes exhibited unique nucleotide substitution pattern with the most abundant transversion from G to T, which was obviously different from the pattern observed in relatives (T to C in groups *Microcerasus*, *Armeniaca*, *Prunus*, and *Amygdalus*, and G to A in groups *Padus* and *Maddenia*) and other Rosaceae taxa (T to C or C to T) (Supplementary Table 7).

In addition, SSRs of Rosaceae plastomes were mainly distributed in the intergenic regions and were predominately composed of A/T with mononucleotides as the most abundant repeat motifs (Supplementary Table 8). Across family Rosaceae plastomes, the SSR number and density were 46–76 and 0.3–0.47 per kb, with an average of 63.72 and 0.4/kb, respectively (Supplementary Table 8 and Supplementary Figure 2B). Tribe Maleae plastomes of subfamily Amygdaloideae harbored the largest number of SSR loci (67–76) in the Rosaceae family, while tribe Amygdaleae plastomes exhibited the greatest difference in the number of SSRs (47–71) (Supplementary Table 8).

#### Gene Evolution in Rosaceae Plastomes

**FIGURE 2:**
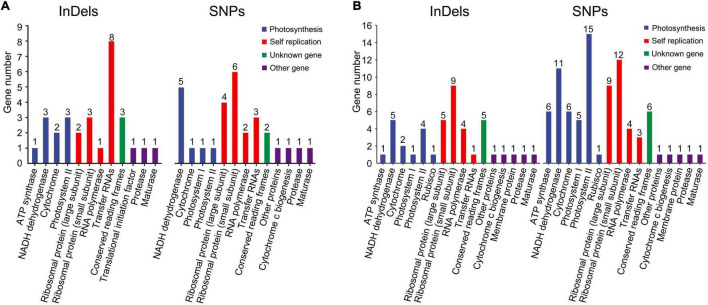
Gene ontology (GO) annotations of plastid genes with high-density SNPs and InDels **(A)** and with effective (high, low, and moderate) effects on gene functions **(B)** in Rosaceae plastomes.

The influence of InDels and SNPs on gene function was investigated in the Rosaceae plastomes (Supplementary Tables 9–12). At the family level, high InDel (≥ 13.47 per kb) and SNP densities (≥ 125.85 per kb) (third quartile, Q3) were detected in 29 genes that were mainly associated with self-replication and photosynthesis (Figure 2A and Supplementary Table 9). Meantime, 635 InDel mutations and 9,290 SNP mutations had a potential effective influence, such as high, moderate, and low potential effects, on 42 and 80 protein-coding genes related to multiple biological activities (Figure 2B and Supplementary Tables 10, 11). Of the 9,290 SNP mutations, 5,404 were synonymous (Table 3 and Supplementary Table 10). The ratio of synonymous and non-synonymous mutations ranged from 0.98 (tribe Maleae) to 1.5 (subfamily Rosoideae) at different taxonomic levels (Table 3). Majority (55.54–60.84%) of the SNP mutations had trivial effects (low level) on gene function (Supplementary Table 12).

**TABLE 3:** Genomic variations distributed in protein-coding regions at different taxonomic levels of Rosaceae.

**Groups**	**Number of SNPs**			**Number of InDels**		
	**Synonymous**	**Non-synonsymous**	**Ratio**	**Three-multiple size**	**All sizes**	**Proportion**
Rosaceae^a^	5,404	3,886	1.39	317	635	0.50
Rosoideae^b^	1,314	875	1.50	32	101	0.32
Amygdaloideae^a^	3,455	2,603	1.33	216	395	0.55
Amygdaleae^a^	997	812	1.23	87	153	0.57
*Cerasus* ^a^	537	445	1.21	45	82	0.55
*Amygdalus* ^c^	105	95	1.11	6	6	1.00
Maleae^d^	350	356	0.98	15	31	0.48
*Malus* ^e^	18	15	1.20	None	None	–
*Pyrus* ^d^	37	34	1.09	None	None	–

*^*a*–*e*^Taking Prunus pseudocerasus (NC030599.1), Fragaria vesca, Prunus persica, Pyrus bretschneideri, and Malus domestica as reference plastome, respectively. None: no insertions-deletions (InDels) were distributed in the protein-coding regions within the plastomes of Malus and Pyrus.*

Insertions-deletions with three-multiple sizes exhibited relatively slighter effects (moderate level) on plastid protein-coding genes than those with other sizes (high level) (Supplementary Table 11). In 121 Rosaceae plastomes, 317 of the 635 identified InDel mutations had a size of three (triplets) (Table 3 and Supplementary Table 11). Of the 317 InDel mutations, 299 (94%) caused disruptive in-frame InDels or conserved in-frame shift, resulting in possibly moderate influence on gene functions. Only 18 InDel mutations adjacent to the start or stop codons led to a high level of effects, which mainly caused start-gain or lost as well as stop-gain or lost mutations (Supplementary Table 11). Furthermore, the proportion of InDels with three-multiple sizes was over 0.5 in the Rosaceae family, and then increased to 0.57 in tribe Amygdaleae but decreased to 0.48 in tribe Maleae (Table 3).

In addition, the GC contents of intergenic regions (0.3095) were significantly lower than those of plastid gene regions (0.4214) (*P* < 2.2e^–16^) across all Rosaceae plastomes (Supplementary Table 13 and Supplementary Figures 3–5). The protein-coding genes of Rosaceae plastomes generally contained lower GC contents (0.2946–0.4894) than tRNA (0.324–0.6216) and rRNA (0.5051–0.5647) (Supplementary Table 13).

To gain further insight into the adaptive evolution of plastid protein-coding genes in the Rosaceae family, 78 plastid protein-coding genes were used to examine the signature of natural selection. Eleven of the 78 genes were detected under Darwinian selection (*ϖ* > 1) (Supplementary Tables 14, 15), namely, 3 self-replication genes (*rpoA*, *rps16*, and *rps18*), 5 photosynthesis genes (*psaA*, *psbL*, *rbcL*, *ndhD*, and *ndhF*), one other gene (*accD*), and two genes with unknown function (*ycf1* and *ycf2*). The *rbcL* gene exhibited the most abundant positively sites (Supplementary Table 15). Nine of the eleven genes underwent highly diverse selection, containing three or more alternative amino acids at one site (Supplementary Table 15). Seven genes (*rpoA*, *rps16*, *rps18*, *ndhD*, *accD*, *ycf1*, and *ycf2*) contained positively selected sites unique within the three subfamily Rosoideae species, *Prinsepia utilis* or *Pentactina rupicola*, which are mainly herbs or brushes (Supplementary Table 15). The footprints of positive selection were specifically detected in *ndhF*, *rpoA*, *rps18*, *rbcL* and *ycf1* genes in most woody trees of tribes Amygdaleae and Maleae (Supplementary Table 15). In addition, for the *Cerasus* taxa, *matK* and *ycf1* were detected under Darwin selection (ω > 1), while no signals of positive selection were detected within the 15 nodes using branch-site model (Supplementary Tables 14, 16 and Supplementary Figure 6).

### Plastome-Based Phylogeny, Dating, and Divergence of Rosaceae

**FIGURE 3:**
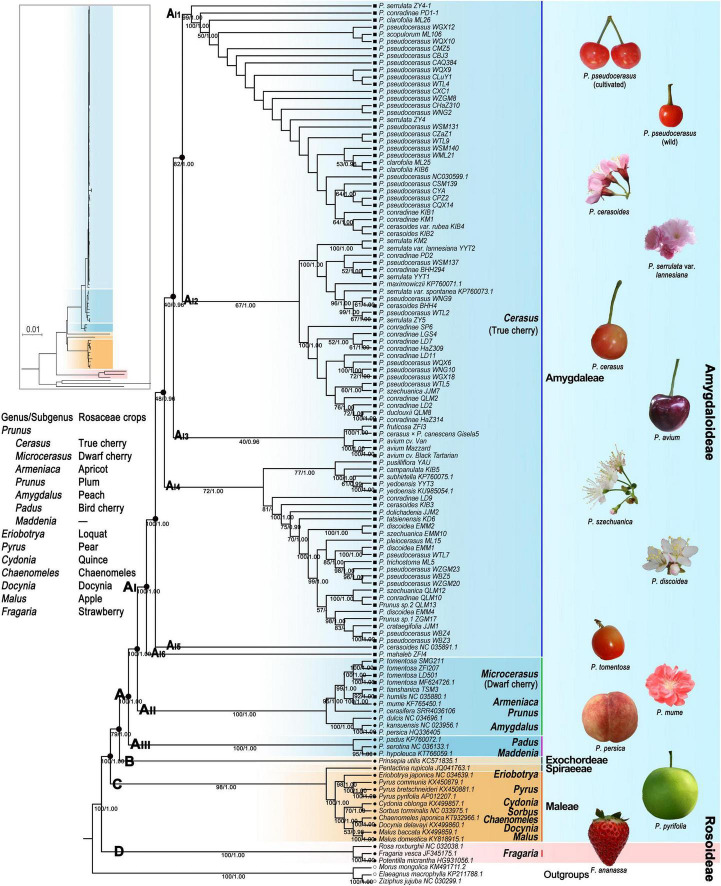
Phylogenetic tree of Rosaceae crops based on the maximum likelihood method under PWGD dataset. Bootstrap (BS)/Bayesian inference (BI) posterior probabilities (PPs) were shown below the branches. PWGD, pruned whole plastome dataset, *n* = 124.

We generated a total of 24 phylogenetic trees with 12 strictly and carefully proceeded datasets using both the ML and BI methods. Aligned sequences for each dataset ranged from 8,515 to 178,338 bp in length (Supplementary Table 17). The best-fit model GTR was set for VSWD and VSPD, and the best-fit model GTR + I + G for the remaining 10 datasets (Supplementary Table 17). The BI and ML trees generated with the same dataset had the same or highly congruent topologies; thus, we only presented the ML tree for each dataset (Figure 3 and Supplementary Figures 7–13). The topology of major clades and their ML bootstrap (BS) and BI posterior probabilities (PPs) are shown in Supplementary Figure 7. Except for the phylogenetic trees generating with exon sequences (PCWD), the remaining phylogenomic analyses suggested that Rosaceae formed four well-supported (78–100% BS, 1 PP) clades (A, B, C, and D) (Figure 3 and Supplementary Figure 7). Clade A included all examined tribe Amygdaleae taxa. Clade B only contained tribe Exochordeae species. Clade C contained taxa from tribes Spiraeeae and Maleae. Clade D corresponded to the three subfamily Rosoideae species.

In all of the ML and BI trees, clade A was further divided into three subclades (A_I_, A_II_, and A_III_) with high support values (100% BS, 1 PP) (Figure 3 and Supplementary Figures 7–13). *Prunus hypoleuca*, *P. serotina*, and *Prunus padus* were assigned as basal subclade A_III_. *Microcerasus* species were grouped with *Armeniaca*, *Prunus*, and the three *Amygdalus* taxa in subclade A_II_. All *Cerasus* taxa formed a distinct monophyletic group (A_I_). Within subclade A_I_, ML and BI trees under eight datasets (WCGD, WOID, PWGD, VSWD, PCGD, POID, PPGD, and VSPD) showed six major groups (A_I__1_, A_I__2_, A_I__3_, A_I__4_, A_I__5_, and A_I__6_) with different support values (37–100% BS, 0.96–1 PP) (Figure 3 and Supplementary Figure 7). In these trees, A_I__1_ contained all cultivated *P. pseudocerasus* accessions, 11 wild *P. pseudocerasus* accessions mainly from Longmenshan Fault Zones, and 11 accessions of six *Cerasus* taxa. A_I__2_ and A_I__4_ consisted of 26 accessions of 9 *Cerasus* taxa and 26 accessions of 16 *Cerasus* taxa, respectively. A_I__3_ was composed of five European cherry accessions of *P. avium*, *P. cerasus* × *P. canescens*, and *P. fruticosa*. The groups A_I__5_ and A_I__6_ corresponded to *P. cerasoides* and *P. mahaleb*, respectively. In the eight datasets, difference was only observed in the BI tree under the VSWD (Supplementary Figure 7A), where A_I__3_ was further divided into two non-sister subgroups, A_I__3_____1_ (*P. avium*) and A_I__3_____2_ (*P. cerasus* × *P. canescens* and *P. fruticosa*). Further subdivision of A_I__2_, A_I__3_, and A_I__4_ was also observed in the phylogenetic trees generated with gene sequence (WGSD and PGSD) and exon sequence (PCWD and PCPD), while these subdivisions were weakly supported (9–73% BS) (Supplementary Figures 7B,C). Overall, despite the aforementioned differences among phylogenetic trees, resemblance between tree topology and taxonomy was completely lost, and no phylogeographic subdivision was detected among true cherries in all the ML and BI trees (Figure 3 and Supplementary Figures 7–13).

**FIGURE 4:**
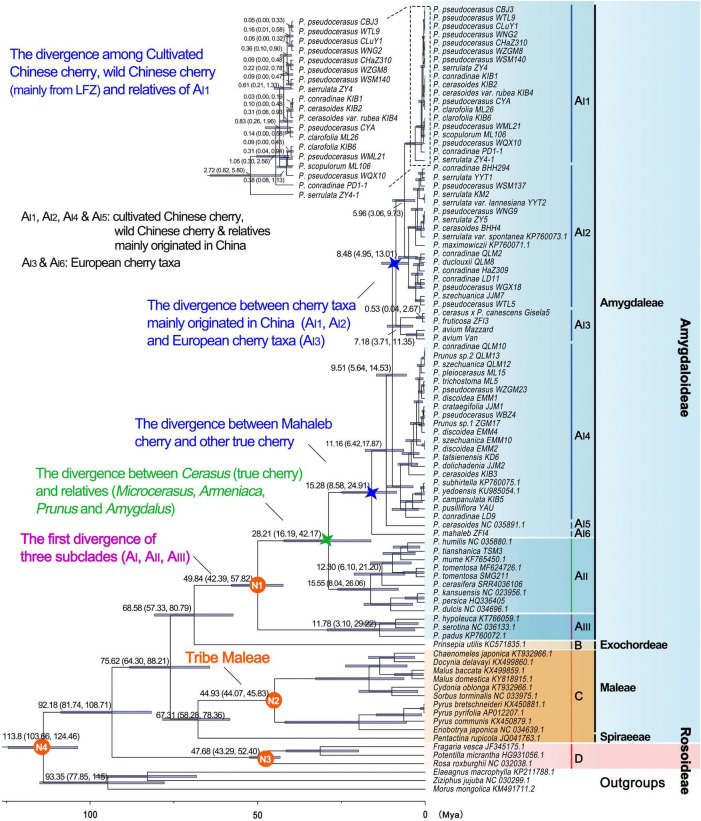
Divergence times of Rosaceae crops based on pruned PWGD. Position of the calibration points N1, N2, N3, and N4 were marked within the tree. Median values and 95% highest posterior density (HPD) were shown next to major branches. The clades A_I__1_-A_I__6_, A_II_, A_III_, B, C, and D correspond to those in Figure 3. The divergence between *Cerasus* (true cherry) and other relatives (*Microcerasus*, *Armeniaca*, *Amygdalus*, and *Prunus*) was marked with green star, and the divergence between European cherry taxa (*Prunus mahaleb*, *Prunus avium*, *Prunus fruticosa*, and *Prunus cerasus* × *Prunus canescens*) and other true cherries were marked with blue stars.

The divergence time between Rosaceae and other Rosales taxa was estimated to be 113.8 Mya (95% highest posterior density (HPD): 103.66–124.46 Mya), and the estimated divergence time for Rosaceae was 92.18 Mya (95% HPD: 81.74–108.71 Mya) (Table 4 and Figure 4). Maleae-Spiraeeae divergence occurred at 67.31 Mya (95% HPD: 58.28–78.36 Mya). The estimated time of divergence of the crown Maleae was 44.93 Mya (95% HPD: 44.07–45.83 Mya). The first divergence of the three subclades A_I_ (*Cerasus*), A_II_ (*Microcerasus*- *Armeniaca*- *Prunus* -*Amygdalus*), and A_III_ (*Padus*-*Maddenia*) occurred at 49.84 Mya (95% HPD: 42.39–57.82 Mya). *Cerasus* (true cherry) and relatives (*Microcerasus*, *Armeniaca*, *Amygdalus*, and *Prunus*) separated from each other at 28.21 Mya (95% HPD: 16.19–42.17 Mya). In subclade A_I_, A_I__6_ (*P. mahaleb*) diverged from the common ancestor of true cherries at 15.28 Mya (95% HPD: 8.58–24.91 Mya), followed by A_I__5_ and A_I__4_ with an estimated divergence time of 11.16 (95% HPD: 6.42–17.87 Mya) and 9.51 Mya (95% HPD: 5.64–14.53 Mya), respectively. European cherry taxa (A_I__3_) and the cherry taxa of A_I__1_ and A_I__2_ diverged at 8.48 Mya (95% HPD: 4.95–13.01 Mya). A_I__1_ and A_I__2_ diverged from each other at 5.96 Mya (95% HPD: 3.06–9.73 Mya). In A_I__1_, the divergence of cultivated Chinese cherry, wild Chinese cherry, and relatives occurred between0.05 (95% HPD: 0–0.32 Mya) and0.83 Mya (95% HPD:0.26–1.96 Mya) (Figure 4).

**TABLE 4:** Estimated divergence times of major clades across Rosaceae using Bayesian method.

**Node**	**Estimated AGES**		
	**Median value (Ma)**	**95% HPD Lower bound**	**95% HPD Upper bound**
Family Rosaceae	113.80	103.66	124.46
Subfamily Amygdaloideae + subfamily Rosoideae	92.18	81.74	108.71
(Tribe Maleae + tribe Spiraeeae) + tribe Amygdaleae	75.62	64.30	88.21
Tribe Maleae + tribe Spiraeeae	67.31	58.28	78.36
Tribe Maleae	44.93	44.07	45.83
(((*Maddenia* + *Padus*^1^*)* + *Padus*^2^) + ((*Amygdalus* + (*Prunus* + (*Microcerasus* + *Armeniaca*))) + *Cerasus*)) + *Prinsepia*	68.58	57.33	80.79
((*Maddenia* + *Padus*^1^*)* + *Padus*^2^) + ((*Amygdalus* + (*Prunus* + (*Microcerasus* + *Armeniaca*))) + *Cerasus*)	49.84	42.39	57.82
(*Maddenia* + *Padus*^1^) + *Padus*^2^	11.78	3.10	29.22
(*Amygdalus* + (*Prunus* + (*Microcerasus* + *Armeniaca*))) + *Cerasus*	28.21	16.19	42.17
*Amygdalus* + (*Prunus* + (*Microcerasus* + *Armeniaca*))	15.55	8.04	26.06
*Prunus* + (*Microcerasus* + *Armeniaca*)	12.30	6.10	21.20
((((A_I__1_ + A_I__2_) + A_I__3_) + A_I__4_) + A_I__5_) + A_I__6_	15.28	8.58	24.91
(((A_I__1_ + A_I__2_) + A_I__3_) + A_I__4_) + A_I__5_	11.16	6.42	17.87
((A_I__1_ + A_I__2_) + A_I__3_) + A_I__4_	9.51	5.64	14.53
(A_I__1_ + A_I__2_) + A_I__3_	8.48	4.95	13.01
A_I__1_ + A_I__2_	5.96	3.06	9.73

*A_*I*__1_, A_*I*__2_,A_*I*__3_, A_*I*__4_, A_*I*__5_, and A_*I*__6_ corresponded to the six groups within A_*I*_ (true cherries) in the maximum likelihood tree of the PWGD dataset. Padus^1^: Prunus serotina; Padus^2^: Prunus padus. HPD: highest posterior density.*

### Diversification of *Cerasus* and Relatives and Genetic Differentiation of Fruiting Cherry Species

#### Plastome-Wide Divergence Within and Among Tribe Amygdaleae and Tribe Maleae

**FIGURE 5:**
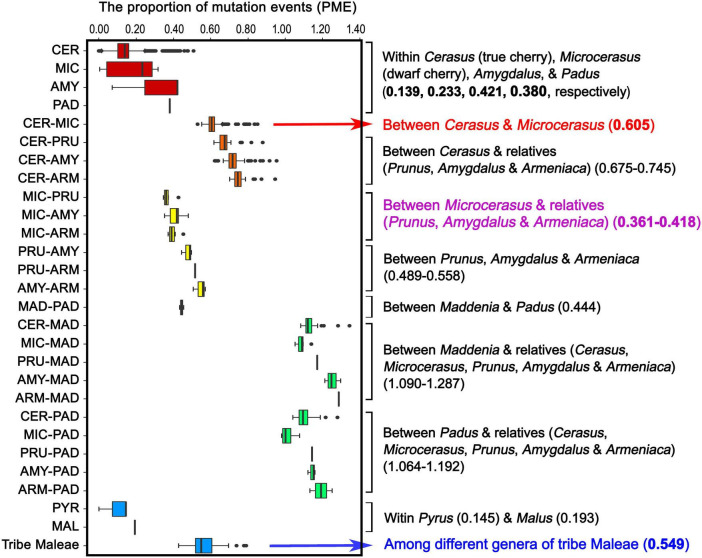
Proportion of mutation events within and among tribes Amygdaleae and Maleae of the Amygdaloideae subfamily. CER, *Cerasus* (true chery); MIC, *Microcerasus* (dwarf cherry); AMY, *Amygdalus* (peach); PAD, *Padus* (bird cherry and black cherry); PRU, *Prunus* (plum); ARM, *Armeniaca* (mei); MAD, *Maddenia*; PYR, *Pyrus* (pear); MAL, *Malus* (apple).

To estimate the levels of plastome-wide divergence for *Cerasus* (true cherry), *Microcerasus* (dwarf cherry), and relatives (peach, plum, apricot, black cherry, and bird cherry), we calculated and compared the values of the PME within and between the tribes Amygdaleae and Maleae (Figure 5 and Supplementary Table 18). High median PME values were observed between *Maddenia* and relatives (1.09–1.287), and between *Padus* and relatives (1.064–1.192) (Figure 5 and Supplementary Table 18). PME values between *Cerasus* and *Microcerasus* (0.605) and between *Cerasus* and other relatives (0.675–0.745) were significantly higher than that among different genera of tribe Maleae (0.549) (Figure 5 and Supplementary Table 18). In addition, the PME values between *Microcerasus* and relatives (*Prunus, Amygdalus* and *Armeniaca*) were only from 0.316 to 0.418 (Figure 5 and Supplementary Table 18), suggesting a close genetic relationship among these taxa. This result was further confirmed by other genetic indexes (genetic distance, genetic differentiation, number of shared InDel and SNP, as well as similarity coefficients) among *Cerasus, Microcerasus*, and relatives (Supplementary Tables 19–21).

#### Genetic Differentiation of Fruiting Cherry Species

Six genetic indexes were calculated to estimate the genetic differentiation among the six fruiting cherry species (*P. pseudocerasus*, *P. avium*, *P. fruticosa*, *P. cerasus* × *P. canescens*, *P. mahaleb*, and *P. tomentosa*) (Supplementary Tables 22, 23). *P. tomentosa* exhibited the highest level of genomic differentiation among the six species, followed by *P. mahaleb* (Supplementary Tables 22, 23). Moderate genetic distances (0.002) and genomic differentiation (0.728) were observed between *P. pseudocerasus* and *P. avium* (Supplementary Table 22). A total of 218 InDel and 321 SNP mutations seemed unique within *P. pseudocerasus*, and 130 InDel and 204 SNP mutations were specifically detected within *P. avium*. Among these unique mutations, 11 InDels and 165 SNPs resulted in effective mutations in 49 protein-coding genes associated with almost all biological functions (Supplementary Table 24). The numbers of shared InDels (54) and SNPs (105) between *P. pseudocerasus* and *P. avium* was much smaller than those between *P. fruticosa* and *P. cerasus* × *P. canescens* (150/389) (Supplementary Table 25).

We further investigated the number of shared InDel and SNP mutations among cultivated *P. pseudocerasus*, wild *P. pseudocerasus*, and their close relatives within group A_I__1_. Eight InDels and nine SNPs were shared between cultivated *P. pseudocerasus* and its close *Cerasus* taxa. These numbers were higher than those between cultivated and wild *P. pseudocerasus* (three shared InDels and one shared SNP), and those between wild *P. pseudocerasus* and close *Cerasus* taxa (two shared InDels and one shared SNP).

## Discussion

### Conserved Genome Structure and Diversified Genomic Variation in Rosaceae Plastomes

Rosaceae plastomes exhibited conserved gene order and gene contents and showed a typical quadripartite structure (LSC, SSC, Ira, and IRb) that has been widely reported in green plants (Howe, 2016; Gao et al., 2019). The overlapping between *matK* and *trnU-UUU*, between *ycf1-*fragment and *ndhF*, and between *psbC* and *psbD* was also detected in most of the Rosaceae plastomes. Ultraconserved *psbC*-*psbD* overlapping regions (53 bp) were identified at the Rosales level. Combining with the results of Theaceae (52 bp) (Huang et al., 2014) and Malvaceae (53 bp) (Xu et al., 2012), we infer that the *psbC*-*psbD* overlapping region may have already been existing before the divergence of flowering plants.

Contraction or expansion of the IR region has been widely proposed to be the main reason for the variation in plastome size (Gao et al., 2019; Xue et al., 2019). Partial losses of plastid genes have been predicted as another main reason for the decreases of some Rosaceae plastomes (Xue et al., 2019). In this study, our data confirm that IR contraction, sequence losses in SSC region, and gene losses (*atpF* and *rps19*-fragment) in LSC regions are predominantly responsible for the relatively small sizes of subfamily Rosoideae plastomes. Our study also verifies that the plastome sizes of subfamily Amygdaloideae are particularly subject to the size increases/decreases in LSC and SSC regions.

The construction of the first accurate map of genomic variation (InDels and SNPs) has widely presented the hotspots across plastomes in Rosaceae. Most of the genomic variations were distributed in intergenic and intronic regions, which was consistent with those reported in rice (Gao et al., 2019), citrus (Bausher et al., 2006), tea (Huang et al., 2014), and other land plants (Howe, 2016; Dobrogojsk et al., 2020). High proportions of synonymous mutations and InDels with three multiple sizes exhibited slight or moderate influences on gene function and expression, suggesting strong constrains to maintain gene functions in Rosaceae plastomes. In addition, since the GC content can greatly influence gene expression (Barahimipour et al., 2016), the universally low GC contents in plastid protein-coding genes and intergenic regions of the Rosaceae plastomes may contribute to maintain efficient biological activities of chloroplasts while responding to diverse and extreme environment and climate changes.

We also detected strong signatures of positive selection in 11 plastid genes (*rpoA*, *rps16*, *rps18*, *psaA*, *psbL*, *rbcL*, *ndhD*, *ndhF*, *accD*, *ycf1*, and *ycf2*), especially in the *rbcL* gene that encodes the large subunit of Rubisco. The evolution of RuBisco large subunit has been thought to be driven by environmental pressures (Hermida-Carrera et al., 2017). Therefore, for Rosaceae crops, the positive selection in this gene probably contribute to their adaptation to various environmental stress (such as low CO_2_ partial pressure) and climate shifts (Hermida-Carrera et al., 2017; Jiang et al., 2018; Yao et al., 2019; Shen et al., 2020). The *NdhF* gene has been suggested to poise the level of redox and consequently maintain or improve the photosynthetic performance of plant crops under extreme temperature and changing light intensity (Martín et al., 2009). The remaining nine genes also play crucial roles in chloroplast protein synthesis, energy transformation and regulation, and photosynthesis. These results indicate the diverse adaptive evolution in plastid genes of Rosaceae crops. Among the 11 plastid genes, 5 photosynthesis-related genes (*rpoA*, *rps18*, *ndhF*, *rbcL*, and *ycf1*) contained positively selected sites unique in most woody fruit trees. The positive selection in the five genes probably help Rosaceae woody fruit trees efficiently capture light energy to produce adequate nutrition to adapt to their growth and development under extreme and variable environmental conditions.

### Distinct Divergence Within and Among Tribes Amygdaleae and Maleae and Taxonomic Implications for Genus *Cerasus*

Tribes Amygdaleae and Maleae of the Rosaceae family contain many economically important crops that exhibit wide adaption to various environments and remarkably diversified phenotypes and genotypes; therefore, the origin, evolution, and domestication of the taxa of the two tribes have been widely explored by botanists and horticulturalists (Yamamoto and Terakami, 2016; Duan et al., 2017; Baek et al., 2018; Wu et al., 2018; Aranzana et al., 2019). Recent genomic and transcriptomic studies (Xiang et al., 2017; Yi et al., 2020) suggested that majority of tribe Amygdaleae members (*x* = 8) only underwent the ancient WGD shared by all Rosaceae members, while tribe Maleae members (*x* = 17) might have experienced two additional WGD events. These different evolution histories possibly contribute to the evolution of distinct fruit types of the two tribes (Xiang et al., 2017). Here, our plastome data also indicate completely different evolutionary patterns between the plastomes of tribes Amygdaleae and Maleae. In comparison with tribe Amygdaleae plastomes, tribe Maleae plastomes exhibited trivial genomic structural variation. This may suggest that the selective pressure from climatic fluctuations, environment changes, and human activities (e.g., domestication) probably results in slighter plastome variation in tribe Maleae than in tribe Amygdaleae.

The plastomes of the Amygdaleae tribe are highly varied in nucleotide substitution and genome structures, implying their highly divergent evolution. Our phylogenomic analyses demonstrated a well-supported relationship of (A_I_ (*Cerasus*) + A_II_ (*Microcerasus*, *Armeniaca*, *Prunus*, and *Amygdalus*)) + A_III_ (*Maddenia* and *Padus*) (Figure 3 and Supplementary Figures 7–13). This topology verifies the three lineages (*Cerasus*-*Prunus*-*Padus*) in previous molecular studies (Wen et al., 2008; Liu et al., 2013; Shi et al., 2013), where the *Prunus* in *Cerasus*-*Prunus*-*Padus* also only contained *Microcerasus*, *Armeniaca*, *Prunus*, and *Amygdalus* but excluded *Cerasus* of the broad-sensed genus *Prunus*. This study suggests that *Cerasus* forms a distinct group and that *Microcerasus* (dwarf cherry) is genetically closer to *Armeniaca*, *Prunus*, and *Amygdalus* than to *Cerasus*. These results can be supported by a recent whole-genome analysis (Wang et al., 2020).

It has been long controversial that *Cerasus* should be treated as a separate genus (de Tournefort, 1700; Linnaeus, 1754; Bate-Smith, 1961; Komarov, 1971; Shishkin and Yuzepchuk, 1971; Yü et al., 1986; Takhtajan, 1997), or as one of the subgenera of the broad-sensed genus *Prunus* (Bentham and Hooker, 1865; Focke, 1894; Schneider, 1905; Koehne, 1911; Rehder, 1940; Ingram, 1948; Hutchinson, 1964; Krüssmann, 1978; Ghora and Panigrahi, 1995). Here, the comparative genomic analyses have revealed that *Cerasus* (true cherry) contained diverse genomic variations and a unique nucleotide substitution pattern with transversion from G to T. The phylogenomic study showed that *Cerasus* was monophyletic and genetically distinct from relatives (*Microcerasus*, *Armeniaca*, *Prunus*, and *Amygdalus*). Molecular dating indicated that *Cerasus* and relatives diverged around the late Oligocene (28.21 (95% HPD: 16.19–42.17) Mya), a period before 66% angiosperm genera in China originated (∼23 Mya) (Lu et al., 2018). The level of plastome-wide divergence between *Cerasus* and relatives (*Microcerasus*, *Armeniaca*, *Prunus*, and *Amygdalus*) was even higher than those among genera of tribe Maleae. Moreover, *Cerasus* taxa show significant morphological differences in lenticels, axillary winter buds, petiole, and endocarp from relatives (*Microcerasus*, *Armeniaca*, *Prunus*, and *Amygdalus*) (Supplementary Table 26 and Supplementary Figure 14), and, generally, *Cerasus* taxa bear inflorescences umbellate or corymbose-racemose with moderate pedicels and conspicuous bracts, while relatives show solitary or two to three sessile flowers with absent bracts (Supplementary Table 26 and Supplementary Figure 14). Therefore, our results strongly support that *Cerasus* (true cherry) is treated as a separate genus, and this will be more convenient for horticulturist. As for the classification of groups *Microcerasus*, *Armeniaca*, *Prunus*, and *Amygdalus*, based on our present results, we prefer to treat them as subgenera or sections of the genus *Prunus* as in the previous study (Supplementary Table 1), while a further analysis of nuclear genome data is necessary.

### Independent Origin of Fruiting Cherry Species

#### Discordance Between Maternal Phylogeny and Traditional Taxonomy and No Phylogeographic Signals Within *Cerasus*

All of our phylogenomic analyses found largely congruent topologies in *Cerasus* taxa (true cherry, subclade A_I_), showing clear discordance between maternal phylogeny and traditional taxonomy, and exhibiting no phylogeographic signals within *Cerasus*. Field investigation has found that abundant wild *Cerasus* taxa with morphologically intermediate forms of multiple *Cerasus* species widely inhabit along Hengduan Mountains and adjacent regions, the eastern edge of QTP and HHM (Chen et al., 2020). Hybridization events among *Cerasus* taxa have been widely reported (Ohta et al., 2007; Shi et al., 2013; Kato et al., 2014; Baek et al., 2018). Especially, a recent analysis based on whole-genome re-sequencing data indicates that four potential inter-specific hybridization events have occurred among *Cerasus* taxa (Yan et al., 2020). Therefore, we inferred that the discordance and lack of phylogeographic signals might have resulted from potential multiple hybridization events within *Cerasus*. Molecular dating in this study indicated that the four major groups (A_I__1_–A_I__4_) diverged in the Middle and Late Miocene [5.96 (95% HPD: 3.06–9.73) -9.51 (95% HPD: 5.64–14.53) Mya], a period consistent with remarkable topographic changes and climatic shifts resulting from the rapid uplift of Qinghai-Tibetan Plateau (QTP) and Himalaya-Hengduan Mountains (HHM) (Favre et al., 2015; Xu et al., 2018). Under this scenario, the long-term topographic changes and climatic shifts in QTP and HHM may have promoted the potential genomic introgressions among *Cerasus* taxa.

#### Independent Origin of Fruiting Cherry Species

**FIGURE 6:**
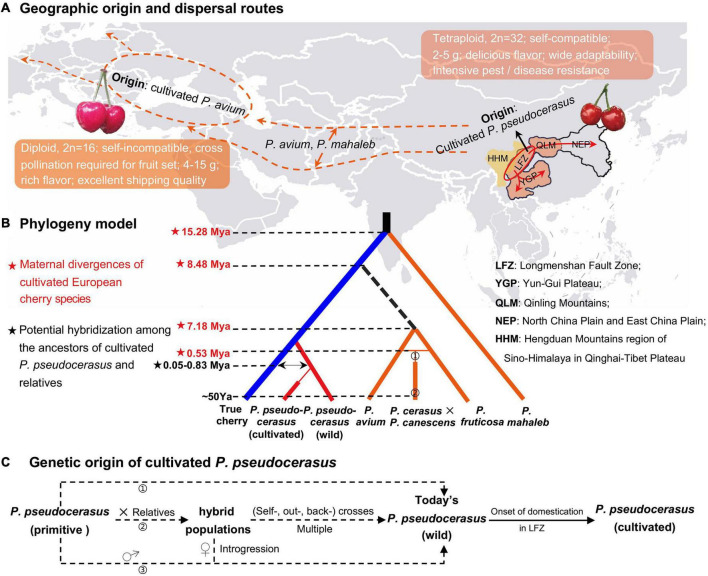
Evolutionary model and demographic history of the fruiting cherry. **(A)** Geographic origin and dispersal routes of *Prunus pseudocerasus*, *P. avium*, and *P. mahaleb*. In the world map, orange dotted line represented the putative dispersal route of *P. avium* and *P. mahaleb*, which inferred from their extant distribution (Faust et al., 2011) and ancestral area construction analysis (Chin et al., 2014). Ovals stood for the geographic origin of cultivated *P. avium* and *P. pseudocerasus*. Red arrows represented the possible dispersal routes of the cultivated *P. pseudocerasus*, which were inferred from whole plastomes (this study), nuclear SSRs (Zhang et al., 2018), intergenic and intronic chloroplast deoxyribonucleic acids (cpDNAs), and internal transcribed spacers (ITS) (Chen et al., 2015). **(B)** Phylogeny of five cherry species. This model was generated according to the present phylogenomic analyses (Figure 3 and Supplementary Figures 7–13), and divergence times were from the molecular dating (Figure 4 and Table 4) and reported studies (Gruppe, 1985). ➀ and ➁ presented the genetic origin of *P. cerasus* and *P. cerasus* × *P. canescens* (Gisela 5). Mya, million years ago; Ya, years ago. **(C)** Genetic origin of cultivated Chinese cherry (*P. pseudocerasus*). Dotted arrows represented three putative paths. In the first path (➀), no gene introgression happened between partial primitive *P. pseudocerasus* and other *Cerasus* taxa. In the second path (➁), multiple hybridization events probably produced numerous interspecific hybrid populations, within and between of which self-, out-, and back-crosses subsequently happened. In the third path (➂), it is also possible that primitive *P. pseudocerasus* (♂) had frequent introgressions into some hybrids (♀) and then produced the wild *P. pseudocerasus* of today.

In this study, we constructed a model to deeply understand the genetic relationship and origin of cultivated cherry (*P. pseudocerasus*, *P. avium*, *P. fruticosa*, *P. cerasus* × *P. canescens*, and *P. mahaleb*) (Figure 6). Fruiting dwarf cherry species *P. tomentosa*, which is significantly genetically distant from *Cerasus* species, was excluded.

European cherry species exhibited relatively distant phylogenetic relationship with the remaining *Cerasus* taxa. In all the phylogenomic analyses (Figure 3 and Supplementary Figures 7–13), *P. mahaleb* was the first that diverged in *Cerasus*, which is consistent with the results of molecular studies based on both nuclear and chloroplast sequences (Shi et al., 2013; Chin et al., 2014). *P. fruticosa*, *P. cerasus* × *P. canescens*, and *P. avium* formed a distinct group (A_I__3_) with moderate to high statistical supports in 16 of the 24 phylogenetic trees (Supplementary Figure 7), which may suggest their close genetic relationship. Given the moderately distant genetic relationship and few shared genomic variations between *P. pseudocerasus* and *P. avium*, we consider that the cultivated Chinese cherry (*P. pseudocerasus/C. pseudocerasus*) and European sweet cherry (*P. avium*/*C. avium*) had their own independent origin, and that few gene introgressions occurred during their domestication process (Figure 6A).

Based on these phylogenomic analyses (Figure 3 and Supplementary Figures 7–13) and molecular dating (Figure 4 and Table 4), integrating previous molecular data (Chin et al., 2014; Chen et al., 2015; Zhang et al., 2018), archeological findings (Liu et al., 2008; Jiang et al., 2021), and historical records (Janick, 2005; Faust et al., 2011; Meyer et al., 2012), a phylogeny model was constructed to summarize the evolutionary history of the five cherry species (Figure 6B). European cherry species diverged with other *Cerasus* taxa at least during Early-Middle Miocene (*P. mahaleb*) and Middle-Late Miocene (*P. avium*), and rapidly spread from eastern and/or western Asia to Europe (Figures 6A,B). Then, *P. cerasus* originated by natural hybridization of *P. fruticosa* (female) and *P. avium* (male) (Janick, 2005; Faust et al., 2011; Figure 6B, ➀), and *P. cerasus* × *P. canescens* (Gisela 5) derived from a cross between *P. cerasus* and *P. canescens* in the 1960s (Gruppe, 1985) (Figure 6B, ➁). In the model, the phylogeny of *P. avium* and *P. fruticosa* needs further verification because of low statistical supports and the subdivision of A_I__3_ in several phylogenetic trees (Supplementary Figure 7).

#### Origin of Cultivated Chinese Cherry

We predominantly traced the origin of cultivated Chinese cherry (*P. pseudocerasus*) (Figure 6C). The aforementioned analyses have revealed the independent origin, relatively distant genetic relationship, and few gene introgressions between *P. pseudocerasus* and *P. avium*. Cultivated *P. avium* has been reported to have domesticated around 3,000–4,000 years ago in the Caspian and Black Seas (Janick, 2005; Faust et al., 2011; Meyer et al., 2012). Archeological research indicated that Chinese cherry (*P. pseudocerasus*) has been cultivated for 3,000 years in China (Liu et al., 2008; Jiang et al., 2021). According to our previous field investigation (Huang et al., 2013; Chen et al., 2016a, 2020) and molecular study based on nuSSRs (Zhang et al., 2018), we have speculated that cultivated *P. pseudocerasus* probably originated from wild *P. pseudocerasus* populations of the Longmenshan Fault Zone (LFZ), the eastern edge of HHM (Zhang et al., 2018).

In this study, phylogenomic analyses indicated that all cultivated *P. pseudocerasus* accessions were clustered with wild *P. pseudocerasus* accessions mainly from LFZ, which strongly supports our previous speculation. Our present plastome data and previous nuclear data (Zhang et al., 2018; Supplementary Table 27) may suggest potential gene introgression among the cultivated *P. pseudocerasus* and its relatives. Given their maternal divergence time (Figure 6B), we speculated that primary hybridization events might occur between primitive *P. pseudocerasus* and relatives. On the basis of these, we proposed a model to describe the possible genetic origins of cultivated *P. pseudocerasus* (Figure 6C). In the first path, no gene introgression happened between partial primitive *P. pseudocerasus* and other *Cerasus* taxa. In the second and third paths, multiple hybridization events between partial primitive *P. pseudocerasus* and relatives probably produced numerous hybrid populations, and then continuous (self-, out- and back-) crosses between hybrids or frequent backcrosses between primitive *P. pseudocerasus* and hybrids occurred. Finally, cultivated *P. pseudocerasus* was domesticated from the wild *P. pseudocerasus* of LFZ around 3,000–4,000 years ago (Zhang et al., 2018) and then dispersed throughout QLM, YGP, and North China Plain and East China Plain (NEP) through the ancient tea-horse road and gallery road (Figure 6A). Our study findings demonstrate that plastome data are an effective tool to explore the geographical origin of the cultivated Chinese cherry. Nevertheless, analyses based on whole-genome re-sequencing data and extensive sampling are still needed to further explore and investigate the potential hybridization events between *P. pseudocerasus* and relatives in the future.

## Data Availability Statement

The datasets presented in this study can be found in online repositories. The names of the repository/repositories and accession number(s) can be found in the article/Supplementary Material.

## Author Contributions

X-RW conceived the project. X-RW and JZ designed the research. JZ performed the experiments. JZ, YW, TC, and QC analyzed the data. JZ and YW drafted and revised the manuscript. X-RW and TC identified the plant materials and revised the manuscript. L-ZG and H-RT revised the manuscript. LW, Z-SL, and RX annotated the plastomes. TC, X-RW, YW, JZ, QC, WH, ML, C-LL, S-FY, YZ, and YL collected the plant materials. M-YL, Y-XL, and Y-TZ provided analysis support. All authors contributed to the article and approved the submitted version.

## Conflict of Interest

The authors declare that the research was conducted in the absence of any commercial or financial relationships that could be construed as a potential conflict of interest.

## Publisher’s Note

All claims expressed in this article are solely those of the authors and do not necessarily represent those of their affiliated organizations, or those of the publisher, the editors and the reviewers. Any product that may be evaluated in this article, or claim that may be made by its manufacturer, is not guaranteed or endorsed by the publisher.

## Footnotes

1
http://www.fao.org/faostat

2
https://www.snapgene.com

3
https://chlorobox.mpimp-golm.mpg.de/geseq.html

4
http://pgrc.ipk-gatersleben.de/misa/misa.html

5
https://sourceforge.net/projects/snpeff/files/

6
https://github.com/rambaut/figtree/releases
