# Supplementary material for: Evolution of Rosaceae Plastomes Highlights Unique Cerasus Diversification and Independent Origins of Fruiting Cherry
Source: Front Plant Sci. 2021 Nov 19;12:736053. doi: 10.3389/fpls.2021.736053 (PMC8639594; doi:10.3389/fpls.2021.736053)
Supplement: Supplementary file 1 [file Data_Sheet_1.pdf]

Supplementary information

Contents

Supplementary Table 1 Taxonomic treatments of true cherry (*Cerasus*) and dwarf cherry (*Microcerasus*)………1
Supplementary Table 2 List of source, locality and GenBank ID of Rosaceae and outgroups in this study………2
Supplementary Table 3 The detailed information of the 91 newly assembled plastomes…………………………6
Supplementary Table 5 Summary of genomic features within 34 complete plastomes…………………………9
Supplementary Table 6 Characteristics of identified InDels and SNPs in Rosaceae plastomes…………………11
Supplementary Table 7 Nucleotide changes and transition / transversion ratio in Rosaceae plastomes…………12
Supplementary Table 8 Characteristics of detected SSRs in 124 plastomes from Rosaceae and outgroups……14
Supplementary Table 9 Identified genes with high InDel and SNP densities at Rosaceae family level…………20
Supplementary Table 12 Annotation results from SnpEffect analysis in Rosaceae plastomes…………………...20
Supplementary Table 14 Estimation of positive selection of plastid protein-coding genes in Rosaceae and *Cerasus* (true cherry)……………………………………………………………………………………………21
Supplementary Table 15 Test for the positive selection of protein-coding genes across Rosaceae plastomes……24
Supplementary Table 16 Test for the positive selection of protein-coding genes in true cherries (*Cerasus*)……28
Supplementary Table 17 Characteristics of plastome sequences and best-fit models of nucleotide substitution for 12 datasets…………………………………………………………………………………………………28
Supplementary Table 18 Proportion of mutation events within and among tribe Amygdaleae and tribe Maleae of the subfamily Amygdaloideae……………………………………………………………………………29
Supplementary Table 19 Genetic distances and genetic differentiation (F_{st}) among *Cerasus*, *Microcerasus* and their close relatives………………………………………………..……………………………………30
Supplementary Table 20 The number of shared InDel and SNP mutations among *Cerasus*, *Microcerasus* and their close relatives……………………………………………………………………………………………30
Supplementary Table 21 The mean values of proportion of mutation events and similarity coefficients among *Cerasus*, *Microcerasus* and their close relatives…………………………………………………………30
Supplementary Table 22 Genetic distances and genetic differentiation (F_{st}) among fruiting cherry species……31
Supplementary Table 23 The mean values of proportion of mutation events and similarity coefficients among fruiting cherry species……………………………………………………...…………………………31
Supplementary Table 24 Protein-coding genes with unique InDels and SNPs within *Prunus pseudocerasus* and within *P. avium*…………………………………………………………….…………………………32
Supplementary Table 25 The number of shared InDel and SNP mutations among fruiting cherry species………32
Supplementary Table 26 Comparison of morphological characteristics of true cherry, dwarf cherry, and close relatives……...……………………………………………………………………………………………33
Supplementary Figure 1 The distribution and length of LSC, SSC, IRs and their neighboring genes in 34 representative Rosaceae plastomes……………………………………………………………..…………34
Supplementary Figure 2 The characteristics of InDels and SSRs across the Rosaceae plastomes………………37
Supplementary Figure 3 GC contents within each inter- and intra-genic region among Rosaceae and outgroups…38
Supplementary Figure 4 GC contents within each inter- and intra-genic region between subfamilies Amygdaloideae and Rosoideae, and among tribes of subfamily Amygdaloideae……………………………39
Supplementary Figure 5 GC contents within each inter- and intra-genic region among *Cerasus* and its relatives…40
Supplementary Figure 6 Detected SNP and InDel among true cherry……………………………………………41

Supplementary Figure 7 The summary of different major clades generated by different data and methods in this study…….………………………………………………………………………………………..42
Supplementary Figure 8 Maximum-likelihood phylogenetic trees constructed with WCGD (A) and PCGD (B) datasets……………………………………………………………………………………………43
Supplementary Figure 9 Maximum-likelihood phylogenetic trees constructed with WOID (A) and POID (B) datasets………..……………………………………………………………………………………44
Supplementary Figure 10 Maximum-likelihood phylogenetic trees constructed with VSWD (A) and VSPD (B) datasets……....……………………………………………………………………………………45
Supplementary Figure 11 Maximum-likelihood phylogenetic trees constructed with WGSD (A) and PGSD (B) datasets……………………………………………………………………………………………46
Supplementary Figure 12 Maximum-likelihood phylogenetic trees constructed with PCWD (A) and PCPD (B) datasets……..……………………………………………………………………………………47
Supplementary Figure 13 Maximum-likelihood phylogenetic tree constructed with PPGD dataset……………48
Supplementary Figure 14 The main morphological differences among true cherry (A-D), dwarf cherry (E-F) and close relatives (G)………………………………………………………………………..……………….49
References……………………………………………………………………..…………...…………….50

Supplementary Table 1 Taxonomic treatments of true cherry (*Cerasus*) and dwarf cherry (*Microcerasus*)

**Genus/Section or Genus/Subgenus/Grex or Section**		**Markers**	**References**
**True cherry**	**Dwarf cherry**		
**Gen. *Prunus*** L. *sensu lato* (*s.l.*)	**Gen. *Prunus*** L. *sensu lato* (*s.l.*)		
Sect. *Cerasus*	Sect. *Cerasoides*	Morphology	Bentham & Hooker, 1865
Subg. *Cerasus*	Subg. *Microcerasus*	Morphology	Focke, 1894; Hutchinson, 1964
Subg. *Cerasus*	Unmentioned[a]	4 chloroplast and 6 nuclear regions	Potter et al., 2007
Subg. *Cerasus* Sects. *Pseudocerasus*, *Eucerasus*, *Mahaleb*	Subg. *Cerasus* Sect. *Microcerasus*	Morphology	Schneider, 1905
Subg. *Cerasus* Grex. *Typocerasus* (2 Sections, 13 Subsections, 11 Series)	Subg. *Cerasus* Grex. *Microcerasus*	Morphology	Koehne, 1911
Subg. *Cerasus* Sects. *Pseudocerasus*, *Lobopetalum*, *Eucerasus*, *Mahaleb*, *Phyllocerasus*, *Phyllomahaleb*	Subg. *Cerasus* Sect. *Microcerasus*	Morphology	Rehder, 1940; Ghora & Panigrahi, 1995
Subg. *Cerasus*	Subg. *Lithocerasus*	Morphology	Ingram, 1948; Krüssmann, 1978
Subg. *Cerasus*	Subg. *Prunus* Sect. *Lithocerasus*	Isozyme	Mowrey & Werner, 1990
Subg. *Cerasus*	Subg. *Prunus* Sect. *Microcerasus*	ITS	Lee & Wen, 2001
		trnL-trnF; ITS, *s*$_6$*pdh*	Bortiri et al., 2001, 2002
		Morphology; *trnL-trnF*, *trnS-trnG*; ITS	Bortiri et al., 2006
		12 chloroplast regions and 3 nuclear genes	Shi et al., 2013
		rbcL, *matK*, *trnL-L-F*, *trnS-S-G*; ITS	Chin et al., 2014
**Gen. *Cerasus***	**Gen. *Cerasus***		
Gen. *Cerasus*	Gen. *Cerasus*	Morphology	de Tournefort, 1700; Linnaeus, 1754; Mill, 1754; Komarov, 1971; Takhtajan, 1997
Subg. *Typocerasus*	Subg. *Microcerasus*	Morphology	Shishkin & Yuzepchuk, 1971
**Subg. *Cerasus***	**Subg. *Microcerasus***	**Morphology; Phenolic constituents**	**Bate-Smith, 1961; Yü et al., 1986**
**Gen. *Cerasus***	**Not classified into gen. *Cerasus***	**Chloroplast genomes**	**This study**

Note: In this study, we followed the Potter's taxonomy system (Potter et al., 2007). [a]: as for the classification of dwarf cherry (*Microcerasus*), because there were no further detailed information in Potter's taxonomy, we here followed the taxonomy system of Rehder (Rehder,1940) according to recent molecular study (Chin et al., 2014).

Supplementary Table 2 List of source, locality and GenBank ID of Rosaceae and outgroups in this study

Rank	Sample Size	Code	Source	Locality			GenBank ID
				Latitude /N	Longitude /E	Altitude /m	
**Family Rosaceae**	**121**						
**Subfamily Amygdaloideae**	**118**						
**Ⅰ. Tribe Amygdaleae**	**106**						
**Genus/Subgenus**[a]							
**1. Gen. *Prunus*** L. *sensu lato* (*s.l.*)							
**1.1 Subg. *Cerasus*** Pers. (28 taxa)	**92**						
**True cherry**	**92**						
P. pseudocerasus Lindl.	**35**						
**Cultivated** (11 Landraces)	**11**						
		CBJ3	Bijie, Guizhou, China	27°18.761’	105°19.492’	1500	MT576869
		CMZ5	Mengzi, Yunnan, China	23°26.856’	103°38.618’	1834	MT576907
		CQX14	Guangyuan, Sichuan, China	32°27.335’	104°48.420’	1264	MT576871
		CPZ2	Pengzhou, Sichuan, China	31°11.988’	103°50.010’	1011	MT576884
		CSM139	Shimian, Sichuan, China	28°57.267’	102°27.707’	1931	MT576885
		CXC1	Xichang, Sichuan, China	27°58.298’	102°06.927’	1762	MT576932
		CYA	Ya’an, Sichuan, China	30°34.334’	102°52.982’	1560	MT576903
		CHaZ310	Hanzhong, Shaanxi, China	33°28.023’	107°58.427’	767	MT576933
		CzaZ1	Zaozhuang, Shandong, China	34°52.648’	117°31.539’	83	MT576851
		CluY1	Luoyang, He’nan, China	34°43.331’	112°18.155’	251	MT576881
		CAQ384	Anqu, Shandong, China	26°13.294’	119°04.292’	127	MT576849
**Wild** (8 populations, 24 individuals)	**24**						
		WBZ3					MT576925
		WBZ4	Bazhong, Sichuan, China	31°55.516’	106°29.873’	854	MT576872
		WBZ5					MT576913
		WGX12	Beichuan, Sichuan, China	32°00.124’	104°38.553’	1404	MT576882
		WGX18					MT576857
		WQX6					MT576924
		WQX9	Guangyuan, Sichuan, China	32°24.099’	104°50.035’	1285	MT576917
		WQX10					MT576893
		WSM131					MT576863
		WSM137	Shimian, Sichuan, China	28°57.267’	102°27.707’	1931	MT576848
		WSM140					MT576862
		WTL2					MT576850
		WTL4					MT576928
		WTL5	Beichuan, Sichuan, China	31°59.304’	104°07.286’	1479	MT576875
		WTL7					MT576853
		WTL9					MT576867
		WZGM8					MT576879
		WZGM20	Mt. Zhougong, Sichuan, China	30°09.114’	102°98.176’	895	MT576864
		WZGM23					MT576921
		WML21	Mt. Kangwu, Sichuan, China	27°56.254’	108°29.315’	3300	MT576874

(Continued)

		WNG2					MT576866
		WNG9	Mt. Nangong, Shaanxi, China	32°13.113’	109°00.535’	879	MT576912
		WNG10					MT576870
		—	NCBI				NC030599.1
P. avium L.	**3**						
**Cultivated**	**2**	‘Black Tartarian’	Zhengzhou Fruit Research Institute, Chinese Academy of Agricultural Sciences, ZFI, CAAS	—	—	—	MT576886
		‘Van’					MT576858
**Wild**	**1**	Mazzard	ZFI, CAAS	—	—	—	MT576923
P. fruticosa Pall.	**1**	ZFI3	ZFI, CAAS	—	—	—	MT576873
P. cerasus L. × *P. canescens*[#]	**1**	‘Gisela5’	ZFI, CAAS	—	—	—	MT576894
P. mahaleb L.	**1**	ZFI4	ZFI, CAAS	—	—	—	MT576896
P. pusilliflora Card.	**1**	YAU	Yunnan Agricultural University	—	—	—	MT576865
P. serrulata Lindl.	**5**						
		KM2	Minzuyuan, Kunming, China	—	—	—	MT576902
		ZY4					MT576900
		ZY4-1	Zunyi, Guizhou, China	27°43.111’	106°43.663’	1015	MT576856
		ZY5					MT576906
		YYT1	Yuyuantan Park, Beijing, China	—	—	—	MT576891
P. serrulata L. var. *spontanea* Max. Wils.	**1**	—	NCBI				KP760073.1
P. serrulata var. *lannesiana* (Carr.) Makino	**1**	YYT2	Yuyuantan Park, Beijing, China	—	—	—	MT576877
P. conradinae Koehne	**15**						
		LD2					MT576854
		LD7	Liangdang, Gansu, China	33°55.256’	106°22.081’	1411	MT576845
		LD9					MT576915
		LD11					MT576887
		HaZ309	Hanzhong, Shaanxi, China	33°28.023’	107°58.427’	767	MT576905
		HaZ314					MT576888
		QLM2	Mt. Qinling, Shaanxi, China	33°43.789’	107°58.373’	1800	MT576908
		QLM10					MT576890
		BHH294	Baihuahu, Guizhou, China	26°39.463’	106°31.460’	1200	MT576899
		LGS4	Mt. Leigong, Guizhou, China	26°22.333’	108°11.526’	1633	MT576876
		KIB1	Kunming Institute of Botany, Chinese Academy of Sciences, KIB, CAS	—	—	—	MT576926
		KM1	Minzuyuan, Kunming, China	—	—	—	MT576931
		PD1-1	Puding, Guizhou, China	26°21.351’	105°54.603’	1451	MT576911
		PD2					MT576922
		SP6	Shiping, Yunnan, China	23°51.995’	102°34.442’	1950	MT576910
P. cerasoides D. Don	**4**						
		BHH4	Baihuahu, Guizhou, China	—	—	—	MT576868

(Continued)

		KIB2	KIB, CAS	—	—	—	MT576895
		KIB3					MT576916
		—	NCBI				NC_035891.1
P. cerasoides D. Don var. *rubea* C. Ingram	**1**	KIB4	KIB, CAS	—	—	—	MT576927
P. campanulata Maxim.	**1**	KIB5	KIB, CAS	—	—	—	MT576846
P. clarofolia Schneid.	**3**						
		ML25	Muli, Sichuan, China	28°12.272’	101°07.997’	2180	MT576883
		ML26					MT576920
		KIB6	KIB, CAS	—	—	—	MT576878
P. szechuanica Batal.	**3**						
		EMM10	Mt. Emei, Sichuan, China	29°34.698’	103°27.495’	614	MT576934
		JJM7	Mt. Jiajin, Sichuan, China	30°48.252’	102°44.183’	3031	MT576901
		QLM12	Mt. Qinling, Shaanxi, China	33°43.789’	107°58.373’	1800	MT576855
P. duclouxii Koehne	**1**	QLM8	Mt. Qinling, Shaanxi, China	33°43.789’	107°58.373’	1800	MT576918
P. pleiocerasus Koehne	**1**	ML15	Muli, Sichuan, China	28°12.272’	101°07.997’	2180	MT576847
P. trichostoma Koehne	**1**	ML5	Muli, Sichuan, China	28°12.272’	101°07.997’	2180	MT576852
P. scopulorum Koehne	**1**	ML106	Muli, Sichuan, China	28°12.272’	101°07.997’	2180	MT576889
P. crataegifolia Hand.-Mazz.	**1**	JJM1	Mt. Jiajin, Sichuan, China	30°48.252’	102°44.183’	3031	MT576880
P. maximowiczii Rupr.	**1**	—	NCBI				KP760071.1
P. tatsienensis Batal.	**1**	KD6	Kangding, Sichuan, China	30°06.245’	102°02.590’	2147	MT576898
P. subhirtella Miquel	**1**	—	NCBI				KP760075.1
P. yedoensis Matsum.	**2**						
		YYT3	Yuyuantan Park, Beijing, China	—	—	—	MT576861
		—	NCBI				KU985054.1
P. discoidea Yü et Li	**3**						
(Not assigned by Potter)							
		EMM1	Mt. Emei, Sichuan, China	29°34.698’	103°27.495’	614	MT576860
		EMM2					MT576909
		EMM4					MT576859
P. dolichadenia Card.	**1**	JJM2	Mt. Jiajin, Sichuan, China	30°48.252’	102°44.183’	3031	MT576929
Prunus sp. 1*	**1**	ZGM17	Mt. Zhougong, Sichuan, China	30°09.114’	102°98.176’	895	MT576897
Prunus sp. 2*	**1**	QLM13	Mt. Qinling, Shaanxi, China	33°43.789’	107°58.373’	1800	MT576904
**1.2.Subg. *Prunus***	**7**						
**Dwarf cherry**	**6**						
Microcerasus (4 species)	**6**						
P. humilis Bge.	**1**	—	NCBI				NC_035880.1
P. tomentosa Thunb.	**4**						
		SMG211	Shuimogou, Xinjiang, China	43°49.450’	87°39.358’	851	MT576919
		LD501	Liangdang, Gansu, China	33°55.256’	106°22.081’	1411	MT576914
		ZFI207	ZFI, CAAS	—	—	—	MT576930
		—	NCBI				MF624726.1
P. tianshanica (Pojarkov) S. Shi[b]	**1**	TSM3	Mt. Tianshan, Xinjiang, China	44°23.307’	80°51.127’	2342	MT576892
P. cerasifera subsp. *myrobalana* Schneid.	**1**		NCBI				SRR4036106

(Continued)

**1.3. Subg. *Armeniaca***	**1**		
P. mume Siebold & Zucc.		NCBI	KF765450.1
**1.4. Subg. *Amygdalus*** (L.) Focke	**3**		
P. kansuensis Rehd.		NCBI	NC_023956.1
P. persica (L.) Batsch		NCBI	HQ336405
P. dulcis		NCBI	NC_034696.1
**1.5. Subg. *Padus*** (Moench) Koehne	**2**		
P. padus L.		NCBI	KP760072.1
P. serotina Ehrhart		NCBI	NC_036133.1
**1.6. Subg. *Maddenia***	**1**		
P. hypoleuca (Koehne) J. Wen[c]		NCBI	KT766059.1
**Ⅱ. Tribe Exochordeae[d]**			
**2. Gen. *Prinsepia*** Royle	**1**		
Prinsepia utilis Royle		NCBI	KC571835.1
**Ⅲ. Tribe Spiraeeae**	**1**		
**3. *Pentactina*** *rupicola* Nakai[d]		NCBI	JQ041763.1
**Ⅳ. Tribe Maleae**	**10**		
**4. *Chaenomeles*** *japonica* (Thunb.) Lindl. Ex Spach	1	NCBI	KT932966.1
**5. *Docynia*** *delavayi* (Franch.) Schneid.	1	NCBI	KX499860.1
**6. *Cydonia*** *oblonga* Mill.	1	NCBI	KX499857.1
**7. *Sorbus*** *torminalis* L.	1	NCBI	NC_033975.1
**8. *Malus*** *baccata* (L.) Borkh.	1	NCBI	KX499859.1
**8. *Malus*** *domestica* Mill.	1	NCBI	KY818915.1
**9. *Pyrus*** *bretschneideri* Rehd.	1	NCBI	KX450881.1
**9. *Pyrus*** *pyrifolia* (Burm. F.) Nakai	1	NCBI	AP012207.1
**9. *Pyrus*** *communis* L.	1	NCBI	KX450879.1
**10. *Eriobotrya*** *japonica* (Thunb.) Lindl.	1	NCBI	NC_034639.1
**Subfamily Rosoideae**	**3**		
**11. *Fragaria*** *vesca* subsp. *vesca* L.	1	NCBI	JF345175.1
**12. *Potentilla*** *micrantha* D. Don	1	NCBI	HG931056.1
**13. *Rosa*** *roxburghii* Tratt.	1	NCBI	NC_032038.1
**Outgroups**	**3**		
**Family Moraceae**			
**14. *Morus*** *mongolica* (Bur.) Schneid.	1	NCBI	KM491711.2
**Family Rhamnaceae**			
**15. *Ziziphus*** *jujuba* Mill.	1	NCBI	NC_030299.1
**Family Elaeagnaceae**			
**16. *Elaeagnus*** *macrophylla* Thunb.	1	NCBI	KP211788.1
**Total**	**124**		

Note: [a]: Potter et al. (2007); [b]: Shi et al. (2013); [c]: Chin et al. (2014); [d]: Takhtajan et al. (1997); * represented two accessions that demonstrated the morphologically intermediate forms of over two *Prunus* taxa; # *P. cerasus* was considered to be the hybrid originated by hybridization of *P. fruticosa* and *P. avium*, and *P. cerasus* × *P. canescens* Gisela 5 derived from a cross between *P. cerasus* and *P. canescens* in the 1960s.

Supplementary Table 3 The detailed information of the 91 newly assembled plastomes

No.	Species	Code	Number of mapped chloroplast reads (bp)	Number of contigs	Number of scaffolds	Assembled length of chloroplast genome (bp)	Coverage (×)
1	*Prunus pseudocerasus*	CBJ3	298,205,820	2	2	157,859	1,889
2		CMZ5	122,388,480	5	5	157,932	775
3		CQX14	62,450,100	6	5	157,936	395
4		CPZ2	210,654,540	3	3	157,719	1,336
5		CSM139	26,940,420	12	10	157,945	171
6		CXC1	138,550,680	4	4	157,741	878
7		CYA	62,743,680	5	5	157,958	397
8		ChaZ310	63,019,440	5	5	157,942	399
9		CzaZ1	236,838,780	4	4	157,857	1,500
10		CluY1	35,705,340	4	4	157,934	226
11		CAQ384	198,997,200	2	2	157,859	1,261
12		WBZ3	74,685,240	6	5	158,006	473
13		WBZ4	279,359,640	5	5	158,060	1,767
14		WBZ5	70,787,520	7	6	157,977	448
15		WGX12	32,337,360	7	6	157,925	205
16		WGX18	52,625,700	8	6	157,917	333
17		WQX6	63,096,660	6	6	157,955	399
18		WQX9	88,108,920	6	5	157,929	558
19		WQX10	69,575,940	3	3	157,871	441
20		WSM131	47,658,600	8	7	157,915	302
21		WSM137	165,462,840	3	3	157,883	1,048
22		WSM140	67,354,020	4	3	157,906	427
23		WTL2	39,277,980	5	5	157,933	249
24		WTL4	58,811,220	6	6	157,962	372
25		WTL5	64,744,200	6	6	158,024	410
26		WTL7	152,154,900	4	3	157,812	964
27		WTL9	89,103,060	5	4	157,911	564
28		WZGM8	65,869,740	5	4	157,950	417
29		WZGM20	148,744,260	6	5	158,002	941
30		WZGM23	113,802,120	6	6	158,015	720
31		WML21	56,561,760	6	6	157,924	358
32		WNG2	167,723,100	3	3	157,882	1,062
33		WNG9	79,866,360	5	5	158,042	505
34		WNG10	181,319,940	6	6	157,940	1,148
35	*Prunus avium*	‘Black Tartarian’	327,237,660	2	2	157,829	2,073
36		‘Van’	227,687,940	2	2	157,889	1,442
37		Mazzard	378,852,120	2	2	157,833	2,400

(Continued)

38	*Prunus fruticosa*	ZFI3	83,776,860	7	7	158,141	530
39	*Prunus cerasus* × *Prunus canescens*	‘Gisela5’	274,449,420	2	2	158,202	1,735
40	*Prunus mahaleb*	ZFI4	546,613,380	4	3	157,579	3,469
41	*Prunus pusilliflora*	YAU	160,007,580	15	9	157,685	1,015
42	*Prunus serrulata*	KM2	43,805,520	5	5	157,892	277
43		ZY4	64,034,640	5	5	157,927	405
44		ZY4-1	49,863,600	6	6	157,898	316
45		ZY5	113,104,800	2	2	157,963	716
46		YYT1	209,473,200	2	2	157,902	1,327
47	*Prunus serrulata* var. *lannesiana*	YYT2	288,302,760	2	2	157,866	1,826
48	*Prunus conradinae*	LD2	79,852,860	6	6	158,014	505
49		LD7	278,188,020	3	3	157,901	1,762
50		LD9	159,555,060	4	4	157,852	1,011
51		LD11	125,944,200	6	6	158167	796
52		HaZ309	95,707,080	4	3	157,923	606
53		HaZ314	67,553,820	2	2	157,837	428
54		QLM2	135,721,260	3	3	157,955	859
55		QLM10	99,266,760	9	8	157,991	628
56		BHH294	77,615,280	4	4	157,992	491
57		LGS4	46,215,000	6	5	158,083	292
58		KIB1	85,215,420	2	2	157,881	540
59		KM1	113,798,880	3	3	157,864	721
60		PD1-1	68,111,280	4	4	157,868	431
61		PD2	95,420,340	4	4	157,405	606
62		SP6	172,020,060	3	3	157,932	1,089
63	*Prunus cerasoides*	BHH4	136,541,880	2	2	157,878	865
64		KIB2	154,377,900	2	2	157,865	978
65		KIB3	188,733,240	5	4	157,812	1,196
66	*Prunus cerasoides* var. *rubea*	KIB4	113,348,340	2	2	157,879	718
67	*Prunus campanulata*	KIB5	182,910,240	5	3	157,708	1,160
68	*Prunus clarofolia*	ML25	42,243,840	6	6	157,939	267
69		ML26	81,193,680	5	5	157,932	514
70		KIB6	195,901,020	4	3	157,884	1,241
71	*Prunus szechuanica*	EMM10	560,242,800	3	2	157,865	3,549
72		JJM7	234,849,960	4	3	157,859	1,488
73		QLM12	148,709,520	4	4	157,966	941
74	*Prunus duclouxii*	QLM8	242,913,960	3	2	157,861	1,539
75	*Prunus pleiocerasus*	ML15	341,882,820	3	2	157,840	2,166

(Continued)

76	*Prunus trichostoma*	ML5	253,301,580	4	3	157,829	1,605
77	*Prunus scopulorum*	ML106	112,118,580	6	6	157,911	710
78	*Prunus crataegifolia*	JJM1	98,416,080	4	3	157,778	624
79	*Prunus tatsienensis*	KD6	295,026,120	4	3	157,807	1,870
80	*Prunus yedoensis*	YYT3	69,302,340	6	5	157,868	439
81	*Prunus discoidea*	EMM1	146,092,140	4	4	157,913	925
82		EMM2	139,651,920	4	4	157,978	884
83		EMM4	70,558,200	3	3	157,894	447
84	*Prunus dolichadenia*	JJM2	217,363,140	3	2	157,874	1,377
85	*Prunus* sp. 1	ZGM17	91,171,260	4	3	157,993	577
86	*Prunus* sp. 2	QLM13	221,861,700	2	2	157,974	1,404
87	*Prunus tomentosa*	SMG211	234,643,140	4	3	157,336	1,491
88		LD501	447,236,100	4	2	157,107	2,847
89		ZFI207	164,126,520	4	2	157,170	1,044
90	*Prunus tianshanica*	TSM3	302,418,360	4	2	157,663	1,918
91	*Prunus cerasifera*	SRR4036106	1,431,370,800	4	2	157,904	9,065
	Total	-	15,071,427,540	-	-	-	-

Note: Raw sequence data of these accession have been deposited into CNGB Sequence Archive (CNSA) of China National GeneBank DataBase (CNGBdb) (https://db.cngb.org/cnsa/home/) with accession number CNP0002213.

Supplementary Table 5 Summary of genomic features within 34 complete plastomes

Species	Genome size (bp)	LSC length (bp)	SSC length (bp)	IR length (bp)
**Subfamily Amygdaloideae**				
**Tribe Amygdaleae**				
***Prunus* subg. *Cerasus* (True cherry)**				
Prunus yedoensis	157,792	85,914	19,120	26,379
Prunus yedoensis YYT3	157,868	86,044	19,042	26,391
Prunus subhirtella	157,833	85,951	19,120	26,381
Prunus serrulata var. *spontanea*	157,882	85,968	19,120	26,397
Prunus pseudocerasus	157,834	85,964	19,084	26,393
Prunus pseudocerasus WNG9	158,042	86,110	19,072	26,430
Prunus pseudocerasus WTL4	157,962	86,081	19,067	26,407
Prunus conradinae LD11	158,059	86,078	19,077	26,452
Prunus cerasoides	157,685	85,792	19,061	26,416
Prunus maximowiczii	157,852	85,847	19,133	26,436
***Prunus* subg. *Prunus* (*Microcerasus*, Dwarf cherry)**				
Prunus tomentosa	158,356	86,630	19,010	26,358
Prunus humilis	158,012	86,133	19,103	26,388
Prunus* subg. *Armeniaca				
Prunus mume	157,712	85,860	19,062	26,395
Prunus* subg. *Amygdalus				
Prunus dulcis	157,723	85,969	18,952	26,401
Prunus kansuensis	157,736	85,840	19,122	26,387
Prunus persica	157,790	85,968	19,060	26,381
Prunus* subg. *Padus				
Prunus serotina	158,778	87,280	18,908	26,295
Prunus padus	158,955	87,666	18,871	26,209
Prunus* subg. *Maddenia				
Prunus hypoleuca	158,084	86,713	18,879	26,246
**Tribe Exochordeae**				
Prinsepia utilis	156,328	85,239	18,480	26,302 (26,307)
**Tribe Spiraeeae**				
Pentactina rupicola	156,612	84,970	18,940	26,351
**Tribe Maleae**				
Pyrus communis	159,901	87,880	19,233	26,394
Pyrus pyrifolia	159,922	87,901	19,237	26,392
Pyrus bretschneideri	159,922	87,901	19,237	26,392
Malus baccata	160,163	88,267	19,188	26,354
Malus domestica	160,068	88,184	19,180	26,352
Chaenomeles japonica	160,088	87,926	19,404	26,379
Cydonia oblonga	159,609	87,823	19,280	26,253
Docynia delavayi	159,428	87,471	19,243	26,357

(Continued)

Eriobotrya japonica	159,137	87,202	19,283	26,326
Sorbus torminalis	160,390	88,250	19,308	26,416
**Subfamily Rosoideae**				
Rosa roxburghii	156,749	85,852	18,791	26,053
Fragaria vesca	155,691	85,606	18,173	25,956
Potentilla micrantha	154,959	84,130	18,761	26,034

Note: LSC: large single-copy region; SSC: one short single-copy region; IR: inverted repeat region.

Supplementary Table 6 Characteristics of identified InDels and SNPs in Rosaceae plastomes

Strategy	Family Rosaceae (n=121)	Subfamily Rosoideae (n=3)	Subfamily Amygdaloideae (n=118)	Tribe Amygdaleae (n=106)	Tribe Exochordeae (n=1)	Tribe Spiraeeae (n=1)	Tribe Maleae (n=10)
				**InDel**			
Number of loci (mutations)	6745 (8976)	2235 (2455)	5149 (6820)	2144 (2781)	1041 (1041)	941 (941)	2134 (2611)
Number of Insertion	5286	1410	3992	1647	488	501	1591
Number of Deletion	3690	1045	2828	1134	533	404	1020
Density of loci (/kb)	42.73	14.16	32.62	13.57	6.60	5.96	13.52
Polymorphic loci	1439	201	1065	419	0	0	322
Region							
LSC	5248	1732	4016	1685	804	712	1700
SSC	968	304	749	319	128	143	293
IRs	529	199	384	140	109	86	141
exon	552	249	360	139	100	78	115
intergenic	5181	1547	4092	1725	791	723	1700
intron	1058	423	736	304	157	147	325
Gene							
protein-coding gene	1389	572	948	387	222	185	386
rRNA	6	4	2	0	2	0	0
tRNA	225	103	151	57	35	41	56
				**SNP**			
Number of loci (mutations)	20817 (23447)	9935 (10327)	14780 (16196)	4846 (5033)	5644 (5644)	4481 (4481)	6228 (6422)
Density of loci (/kb)	131.89	62.95	93.64	30.70	35.76	28.39	39.46
Polymorphic loci							
biallelic position	2298	378	1276	173	0	0	184
triallelic position	166	7	70	7	0	0	5
Region							
LSC	14692	6817	10577	3498	3990	3056	4519
SSC	4249	2094	3134	1043	1219	1099	1315
IRs	1876	1024	1069	305	435	326	394
exon	8513	4668	5703	1777	2399	1915	2313
intergenic	9811	4017	7357	2504	2557	2053	3153
intron	2706	1374	1868	614	771	564	838
Gene							
Protein-coding gene	10268	5517	6930	2167	2890	2276	2872
rRNA	81	40	48	13	8	20	15
tRNA	991	544	672	235	290	211	295

Note: Taking *Prunus pseudocerasus* (NC030599.1) as the referencing genome. The numbers of InDel and SNP loci in one intron (129477 bp: 99835-71477) of *rps12* were not included due to the special gene structure.

Supplementary Table 7 Nucleotide changes and transition / transversion ratio in Rosaceae plastomes

Substitution	Family Rosaceae (n=121)		Subfamily Rosoideae (n=3)		Subfamily Amygdaloideae (n=118)		Tribe Amygdaleae (n=106)		Tribe Exochordeae (n=1)		Tribe Spiraeeae (n=1)		Tribe Maleae (n=10)	
	Number	Ratio	Number	Ratio	Number	Ratio	Number	Ratio	Number	Ratio	Number	Ratio	Number	Ratio
A→C	2004	0.09	966	0.09	1284	0.08	303	0.06	392	0.07	409	0.09	630	0.10
A→G	2796	0.12	1341	0.13	1848	0.11	569	0.11	762	0.14	485	0.11	675	0.11
A→T	1439	0.06	481	0.05	1088	0.07	357	0.07	325	0.06	223	0.05	437	0.07
C→A	1643	0.07	516	0.05	1309	0.08	553	0.11	443	0.08	315	0.07	412	0.06
C→G	620	0.03	309	0.03	379	0.02	80	0.02	138	0.02	124	0.03	156	0.02
C→T	**3028**	**0.13**	**1479**	**0.14**	**2035**	**0.13**	614	0.12	740	0.12	**655**	**0.15**	**829**	**0.13**
G→A	2867	0.12	1438	0.14	1939	0.12	577	0.11	695	0.12	621	0.14	782	0.12
G→C	656	0.03	316	0.03	407	0.03	95	0.02	148	0.03	121	0.03	160	0.02
G→T	1714	0.07	543	0.05	1369	0.08	596	0.12	442	0.08	310	0.07	426	0.07
T→A	1519	0.06	548	0.05	1098	0.07	354	0.07	318	0.06	219	0.05	475	0.07
T→C	2941	0.13	1367	0.13	1996	0.12	**632**	**0.13**	**815**	**0.14**	536	0.12	722	0.11
T→G	2221	0.09	1023	0.10	1445	0.09	303	0.06	425	0.08	463	0.10	718	0.11
Ts/Tv ratio	0.98		1.20		0.93		0.91		1.14		1.05		0.88	

Substitution	*Fragaria vesca*		*Potentilla micrantha*		*Rosa roxburghii*		*Malus* (n=2)		*Pyrus* (n=3)		*Eriobotrya japonica*		*Sorbus torminalis*		*Cydonia oblonga*		*Docynia delavayi*		*Chaenomeles japonica*	
	Number	Ratio	Number	Ratio	Number	Ratio	Number	Ratio	Number	Ratio	Number	Ratio	Number	Ratio	Number	Ratio	Number	Ratio	Number	Ratio
A→C	604	0.09	619	0.09	589	0.09	404	0.09	398	0.09	395	0.09	385	0.09	390	0.09	402	0.09	413	0.09
A→G	914	0.14	860	0.13	861	0.14	525	0.12	516	0.12	516	0.12	508	0.12	503	0.12	501	0.12	500	0.11
A→T	274	0.04	265	0.04	274	0.04	249	0.06	225	0.05	224	0.05	223	0.05	212	0.05	216	0.05	232	0.05
C→A	346	0.05	312	0.05	297	0.05	306	0.07	299	0.07	292	0.07	277	0.07	291	0.07	289	0.07	301	0.07
C→G	183	0.03	216	0.03	186	0.03	111	0.02	107	0.02	116	0.03	106	0.03	107	0.03	108	0.03	110	0.02
C→T	**989**	**0.15**	**984**	**0.15**	**927**	**0.15**	**623**	**0.14**	**612**	**0.14**	**600**	**0.14**	**604**	**0.14**	**598**	**0.14**	**607**	**0.14**	**622**	**0.14**
G→A	984	0.15	973	0.15	878	0.14	576	0.13	572	0.13	572	0.13	551	0.13	551	0.13	556	0.13	578	0.13
G→C	176	0.03	200	0.03	171	0.03	111	0.02	107	0.02	103	0.02	104	0.02	107	0.03	99	0.02	104	0.02
G→T	348	0.05	348	0.05	300	0.05	298	0.07	285	0.07	279	0.06	283	0.07	267	0.06	276	0.06	289	0.07
T→A	326	0.05	297	0.05	309	0.05	251	0.06	247	0.06	244	0.06	219	0.05	212	0.05	241	0.06	249	0.06
T→C	943	0.14	871	0.13	921	0.15	547	0.12	551	0.13	542	0.13	550	0.13	524	0.13	545	0.13	542	0.12
T→G	650	0.1	648	0.1	622	0.1	454	0.1	457	0.1	442	0.1	430	0.1	428	0.1	428	0.1	461	0.1
Ts/Tv ratio	1.32		1.27		1.31		1.04		1.06		1.06		1.09		1.08		1.07		1.04	

(Continued)

Substitution	Subg. *Cerasus* (true cherry, n=92)		*Microcerasus* (dwarf cherry, n=6)		Subg. *Armeniaca* (*Prunus Mume*)		Subg. *Amygdalus* (n=3)		Subg. *Prunus* (*Prunus cerasifera*)		Subg. *Padus* (n=2)		Subg. *Maddenia* (*Prunus hypoleuca*)	
	Number	Ratio	Number	Ratio	Number	Ratio	Number	Ratio	Number	Ratio	Number	Ratio	Number	Ratio
A→C	102	0.05	94	0.09	75	0.08	78	0.07	64	0.08	143	0.09	121	0.09
A→G	136	0.07	114	0.11	112	0.13	152	0.13	89	0.12	198	0.12	167	0.12
A→T	152	0.08	82	0.08	52	0.06	80	0.07	28	0.04	116	0.07	85	0.06
C→A	284	0.15	75	0.07	63	0.07	90	0.08	62	0.08	128	0.08	111	0.08
C→G	22	0.01	19	0.02	13	0.01	18	0.02	8	0.01	34	0.02	26	0.02
C→T	243	0.13	130	0.12	125	0.14	141	0.12	100	0.13	202	0.12	176	0.13
G→A	210	0.11	119	0.11	98	0.11	124	0.11	101	0.13	**210**	**0.13**	**182**	**0.13**
G→C	31	0.02	25	0.02	20	0.02	26	0.02	18	0.02	39	0.02	28	0.02
G→T	**286**	**0.15**	103	0.10	75	0.08	116	0.10	70	0.09	131	0.08	122	0.09
T→A	148	0.08	75	0.07	64	0.07	80	0.07	46	0.06	111	0.07	82	0.06
T→C	163	0.09	**169**	**0.16**	**126**	**0.14**	**190**	**0.16**	**116**	**0.15**	187	0.11	162	0.12
T→G	117	0.06	77	0.07	72	0.08	80	0.07	65	0.08	133	0.08	109	0.08
Ts/Tv ratio	0.66		0.97		1.06		1.07		1.12		0.95		1.00	

Note: the richest nucleotide alternation within each taxonomic level were bold and marked with red color. Taking *Prunus pseudocerasus* (NC030599.1) as the referencing genome. Ts/Tv: Transition / Transversion.

Supplementary Table 8 Characteristics of detected SSRs in 124 plastomes from Rosaceae and outgroups

No.	Species	Code	Total number	SSR density (/kb)	Number of SSRs in genomic regions				Mono-nucleotide repeats					Di-nucleotide repeats		Tri-nucleotide repeats	
					LSC & SSC	IRs	Inter-genic	Intra-genic	Total number	A	T	G	C	Total number	Repeat motif	Total number	Repeat motif
1	*Prunus pseudocerasus*	CBJ3	64	0.41	61	3	44	20	60	21	38	1	0	4	AT, TA	0	-
2		CMZ5	63	0.40	61	2	43	20	60	21	38	1	0	3	AT	0	-
3		CQX14	62	0.39	59	3	41	21	59	21	37	1	0	3	AT	0	-
4		CPZ2	64	0.41	62	2	44	20	60	21	38	1	0	4	AT, TA	0	-
5		CSM139	62	0.39	59	3	41	21	60	21	38	1	0	2	AT	0	-
6		CXC1	63	0.40	61	2	43	20	60	21	38	1	0	3	AT	0	-
7		CYA	64	0.41	61	3	43	21	61	22	38	1	0	3	AT	0	-
8		ChaZ310	64	0.41	61	3	43	21	60	22	37	1	0	4	AT, TA	0	-
9		CzaZ1	64	0.41	62	2	44	20	60	21	38	1	0	4	AT, TA	0	-
10		CluY1	63	0.40	61	2	44	19	60	21	38	1	0	3	AT	0	-
11		CAQ384	64	0.41	61	3	44	20	60	21	38	1	0	4	AT, TA	0	-
12		WBZ3	63	0.40	60	3	39	24	60	22	38	0	0	3	AT, TA	0	-
13		WBZ4	63	0.40	60	3	39	24	61	23	38	0	0	2	AT	0	-
14		WBZ5	63	0.40	60	3	39	24	59	22	37	0	0	4	AT, TA	0	-
15		WGX12	64	0.41	61	3	43	21	60	22	37	1	0	4	AT, TA	0	-
16		WGX18	62	0.39	57	5	39	23	59	21	37	0	1	3	AT, TA	0	-
17		WQX6	66	0.42	61	5	41	25	62	24	37	0	1	4	AT, TA	0	-
18		WQX9	63	0.40	60	3	42	21	59	21	37	1	0	4	AT, TA	0	-
19		WQX10	64	0.41	61	3	43	21	60	22	37	1	0	4	AT, TA	0	-
20		WSM131	64	0.41	61	3	43	21	60	22	37	1	0	4	AT, TA	0	-
21		WSM137	66	0.42	63	3	43	23	62	22	39	1	0	4	AT, TA	0	-
22		WSM140	64	0.41	61	3	43	21	61	22	38	1	0	3	AT	0	-

(Continued)

23		WTL2	70	0.44	67	3	42	28	67	25	41	1	0	3	AT	0	-
24		WTL4	62	0.39	59	3	41	21	59	21	37	1	0	3	AT	0	-
25		WTL5	68	0.43	65	3	43	25	65	23	40	1	1	3	AT, TA	0	-
26		WTL7	63	0.40	61	2	39	24	59	21	37	0	1	4	AT, TA	0	-
27		WTL9	64	0.41	61	3	43	21	61	22	38	1	0	3	AT	0	-
28		WZGM20	65	0.41	62	3	40	25	61	22	38	0	1	4	AT, TA	0	-
29		WZGM23	64	0.41	61	3	39	25	61	22	38	0	1	3	AT	0	-
30		WZGM8	64	0.41	61	3	43	21	61	22	38	1	0	3	AT	0	-
31		WML21	64	0.41	61	3	43	21	60	22	37	1	0	4	AT, TA	0	-
32		WNG2	65	0.41	62	3	44	21	61	22	38	1	0	4	AT, TA	0	-
33		WNG9	70	0.44	68	2	46	24	67	24	42	1	0	3	AT	0	-
34		WNG10	66	0.42	61	5	41	25	62	24	37	0	1	4	AT, TA	0	-
35		NC_030599.1	64	0.41	62	2	44	20	60	21	38	1	0	4	AT, TA	0	-
36	*Prunus avium*	‘Black Tartarian’	61	0.39	59	2	40	21	56	20	35	1	0	5	AT, TA	0	-
37		‘Van’	59	0.37	57	2	37	22	54	20	33	1	0	5	AT, TA	0	-
38		Mazzard	61	0.39	59	2	40	21	56	20	35	1	0	5	AT, TA	0	-
39	*Prunus fruticosa*	ZFI3	61	0.39	59	2	39	22	56	21	34	1	0	4	AT	1	$(ATA)_5$
40	*Prunus cerasus* × *Prunus canescens*	‘Gisela5’	65	0.41	63	2	44	21	56	22	33	1	0	8	AT, TA	1	$(ATA)_5$
41	*Prunus mahaleb*	ZFI4	66	0.42	63	3	40	26	63	24	38	1	0	3	AT	0	-
42	*Prunus pusilliflora*	YAU	57	0.36	55	2	38	19	53	18	35	0	0	4	AT, TA	0	-
43	*Prunus serrulata*	KM2	67	0.42	64	3	44	23	64	23	40	1	0	3	AT	0	-
44		ZY4	63	0.40	60	3	42	21	60	22	37	1	0	3	AT	0	-
45		ZY4-1	61	0.39	58	3	40	21	58	22	35	1	0	3	AT	0	-
46		ZY5	70	0.44	67	3	46	24	66	24	41	1	0	4	AT, TA	0	-

(Continued)

47		YYT1	65	0.41	62	3	41	24	61	22	39	0	0	4	AT, TA	0	-
48	*Prunus serrulata* var. *spontanea*	KP760073.1	69	0.44	66	3	46	23	65	24	40	1	0	4	AT, TA	0	-
49	*Prunus serrulata* var. *lannesiana*	YYT2	69	0.44	67	2	43	26	65	23	41	1	0	4	AT, TA	0	-
50	*Prunus conradinae*	LD2	65	0.41	62	3	42	23	63	20	41	1	1	2	AT, TA	0	-
51		LD7	67	0.42	64	3	44	23	64	23	40	1	0	3	AT, TA	0	-
52		LD9	63	0.40	60	3	40	23	59	23	35	1	0	4	AT, TA	0	-
53		LD11	65	0.41	63	2	41	24	62	21	39	1	1	3	AT, TA	0	-
54		HaZ309	66	0.42	63	3	44	22	62	21	40	1	0	4	AT, TA	0	-
55		HaZ314	70	0.44	68	2	45	25	65	23	40	1	1	5	AT, TA	0	-
56		QLM2	66	0.42	63	3	43	23	63	21	41	1	0	3	AT, TA	0	-
57		QLM10	60	0.38	57	3	36	24	57	22	34	0	1	3	AT	0	-
58		BHH294	70	0.44	68	2	46	24	67	24	42	1	0	3	AT	0	-
59		LGS4	65	0.41	63	2	41	24	62	22	38	1	1	3	AT, TA	0	-
60		KIB1	64	0.41	61	3	44	20	60	21	38	1	0	4	AT, TA	0	-
61		KM1	64	0.41	61	3	44	20	60	21	38	1	0	4	AT, TA	0	-
62		PD1-1	64	0.41	61	3	43	21	60	22	37	1	0	4	AT, TA	0	-
63		PD2	71	0.45	68	3	44	27	67	24	41	1	1	4	AT	0	-
64		SP6	66	0.42	64	2	41	25	62	22	38	1	1	4	AT, TA	0	-
65	*Prunus cerasoides*	BHH4	69	0.44	66	3	45	24	65	23	41	1	0	4	AT, TA	0	-
66		KIB2	64	0.41	61	3	44	20	60	21	38	1	0	4	AT, TA	0	-
67		KIB3	64	0.41	61	3	39	25	60	23	36	0	1	4	AT, TA	0	-
68		NC_035891.1	63	0.40	60	3	39	24	58	22	35	1	0	5	AT, TA	0	-
69	*Prunus cerasoides* var. *rubea*	KIB4	64	0.41	61	3	44	20	60	21	38	1	0	4	AT, TA	0	-
70	*Prunus campanulata*	KIB5	59	0.37	57	2	34	25	55	20	34	0	1	4	AT, TA	0	-
71	*Prunus clarofolia*	ML25	63	0.40	60	3	42	21	60	22	37	1	0	3	AT	0	-
72		ML26	63	0.40	60	3	42	21	60	22	37	1	0	3	AT	0	-

(Continued)

73		KIB6	64	0.41	61	3	44	20	60	21	38	1	0	4	AT, TA	0	-
74	*Prunus szechuanica*	EMM10	64	0.41	61	3	40	24	60	21	37	1	1	4	AT, TA	0	-
75		JJM7	70	0.44	67	3	45	25	66	23	41	1	1	4	AT, TA	0	-
76		QLM12	63	0.40	60	3	39	24	59	22	36	0	1	4	AT, TA	0	-
77	*Prunus duclouxii*	QLM8	69	0.44	66	3	44	25	65	23	40	1	1	4	AT, TA	0	-
78	*Prunus pleiocerasus*	ML15	62	0.39	60	2	38	24	58	20	37	0	1	4	AT, TA	0	-
79	*Prunus trichostoma*	ML5	64	0.41	62	2	40	24	60	21	38	0	1	4	AT, TA	0	-
80	*Prunus scopulorum*	ML106	64	0.41	61	3	43	21	60	22	37	1	0	4	AT, TA	0	-
81	*Prunus crataegifolia*	JJM1	63	0.40	61	2	40	23	60	22	38	0	0	3	AT, TA	0	-
82	*Prunus maximowiczii*	KP760071.1	63	0.40	61	2	41	22	60	20	39	1	0	3	AT	0	-
83	*Prunus tatsienensis*	KD6	66	0.42	63	3	42	24	61	21	39	0	1	4	AT, TA	1	$(ATA)_5$
84	*Prunus subhirtella*	KP760075.1	60	0.38	58	2	35	25	56	20	35	0	1	4	AT, TA	0	-
85	*Prunus yedoensis*	YYT3	59	0.37	57	2	34	25	55	20	34	0	1	4	AT, TA	0	-
86		KU985054.1	47	0.30	45	2	28	19	43	17	25	0	1	4	AT, TA	0	-
87	*Prunus discoidea*	EMM1	64	0.41	61	3	39	25	60	22	37	0	1	4	AT, TA	0	-
88		EMM2	65	0.41	62	3	40	25	61	22	37	1	1	4	AT, TA	0	-
89		EMM4	64	0.41	61	3	41	23	61	23	38	0	0	3	AT, TA	0	-
90	*Prunus dolichadenia*	JJM2	62	0.39	59	3	39	23	58	21	36	0	1	4	AT, TA	0	-
91	*Prunus* sp. 1	ZGM17	62	0.39	60	2	39	23	60	22	38	0	0	2	AT	0	-
92	*Prunus* sp. 2	QLM13	63	0.40	60	3	39	24	59	22	36	0	1	4	AT, TA	0	-
93	*Prunus humilis*	NC_035880.1	48	0.30	46	2	33	15	47	14	30	1	2	1	AT, TA	0	-
94	*Prunus tomentosa*	SMG211	54	0.34	52	2	31	23	52	21	28	2	1	2	AT	0	-
95		LD501	56	0.36	54	2	34	22	52	21	28	2	1	4	AT, TA	0	-
96		ZFI207	54	0.34	52	2	31	23	52	21	28	2	1	2	AT	0	-
97		MF624726.1	56	0.35	54	2	34	22	52	21	28	2	1	4	AT, TA	0	-
98	*Prunus tianshanica*	TSM3	58	0.37	56	2	39	19	52	19	29	2	2	6	AT, TA	0	-

(Continued)

99	*Prunus mume*	KF765450.1	57	0.36	55	2	35	22	53	20	29	2	2	4	AT, TA	0	-
100	*Prunus cerasifera*	SRR4036106	54	0.34	52	2	36	18	51	20	28	2	1	3	AT, TA	0	-
101	*Prunus kansuensis*	NC_023956.1	55	0.35	52	3	34	21	49	18	27	1	3	6	AT, TA	0	-
102	*Prunus persica*	HQ336405	60	0.38	57	3	38	22	56	21	29	2	4	4	AT, TA	0	-
103	*Prunus dulcis*	NC_034696.1	52	0.33	50	2	35	17	47	14	29	2	2	5	AT, TA	0	-
104	*Prunus padus*	KP760072.1	63	0.40	63	0	41	22	56	22	34	0	0	6	AT, TA	1	$(ATA)_5$
105	*Prunus serotina*	NC_036133.1	70	0.44	70	0	45	25	67	22	44	0	1	3	AT, TA	0	-
106	*Prunus hypoleuca*	KT766059.1	64	0.40	61	3	43	21	57	19	38	0	0	7	AT, TA	0	-
107	*Prinsepia utilis*	KC571835.1	61	0.39	54	7	37	24	59	17	41	0	1	2	AT, TA	0	-
108	*Pentactina rupicola*	JQ041763.1	54	0.34	52	2	40	14	52	22	28	1	1	2	AT, TA	0	-
109	*Chaenomeles japonica*	KT932966.1	76	0.47	74	2	57	19	73	34	37	1	1	3	AT, TA	0	-
110	*Docynia delavayi*	KX499860.1	75	0.47	73	2	55	20	72	26	41	1	4	3	AT, TA	0	-
111	*Cydonia oblonga*	KX499857.1	73	0.46	71	2	57	16	72	25	45	0	2	1	AT	0	-
112	*Sorbus torminalis*	NC_033975.1	71	0.44	69	2	55	16	68	26	39	1	2	3	AT, TA	0	-
113	*Malus baccata*	KX499859.1	74	0.46	70	4	54	20	71	28	40	0	3	3	AT	0	-
114	*Malus domestica*	KY818915.1	67	0.42	63	4	50	17	65	28	34	0	3	2	AT	0	-
115	*Pyrus bretschneideri*	KX450881.1	70	0.44	68	2	50	20	67	31	34	0	2	3	AT, TA	0	-
116	*Pyrus pyrifolia*	AP012207.1	70	0.44	68	2	50	20	67	31	34	0	2	3	AT, TA	0	-
117	*Pyrus communis*	KX450879.1	73	0.46	71	2	52	21	70	32	36	0	2	3	AT, TA	0	-
118	*Eriobotrya japonica*	NC_034639.1	72	0.45	68	4	53	19	70	27	41	0	2	2	AT, TA	0	-
119	*Fragaria vesca*	JF345175.1	46	0.30	39	7	34	12	41	17	21	1	2	4	AT, TA	1	$(TTA)_5$
120	*Potentilla micrantha*	HG931056.1	62	0.40	56	6	39	23	54	23	28	1	2	8	AT, TA	0	-
121	*Rosa roxburghii*	NC_032038.1	53	0.34	49	4	34	19	50	21	27	0	2	3	AT	0	-
	Overall	Mean value	63.72	0.40	60.83	2.74	41.64	21.93	59.89	22	36.46	0.75	0.68	3.64	-	0.04	-
**Outgroups**																	-
122	*Morus mongolica*	KM491711.2	62	0.39	60	0	56	6	58	18	40	0	0	4	AT, TA	0	-

123	*Ziziphus jujuba*	NC_030299.1	62	0.38	60	2	50	12	57	21	36	0	0	4	AT	1	$(ATT)_5$
124	*Elaeagnus macrophylla*	KP211788.1	48	0.32	45	3	38	10	43	17	26	0	0	5	AT, TA	0	-

Note: -: not detected.

Supplementary Table 9 Identified genes with high InDel and SNP densities at Rosaceae family level

Type of variation (Number of identified genes)	Genes
INDEL (n=29)	*atpF, clpP, infA, matK, ndhA, ndhF, ndhI, petB, petD, psbK, psbL, psbT, rpl16, rpl22, rpoC1, rps16, rps18, rps19*-fragment, *ycf1, ycf1*-fragment, *ycf3, trnE-UCC, trnG-GCC, trnG-UCC, trnK-UUU, trnL-UAA, trnM-CAU, trnV-UAC, trnW-CCA*
SNP (n=29)	*accD, ccsA, clpP, infA, matK, ndhA, ndhD, ndhF, ndhG, ndhI, petB, psaI, psbK, rpl16, rpl22, rpl32, rpl33, rpoA, rpoC2, rps11, rps15, rps16, rps19, rps3, rps8, ycf1, trnG-UCC, trnK-UUU, trnL-UAA*

Note: The quartile values were estimated by the summary function in Rstudio. Here, if the InDel/SNP density of a gene was larger than the quartile values, we considered it containing high-density InDel/SNP. The numbers of genes with high InDel and SNP densities were 29 and 29, respectively.

Supplementary Table 12 Annotation results from snpEffect analysis in Rosaceae plastomes

Strategy	Family Rosaceae	Subfamily Rosoideae	Subfamily Amygdaloideae	Tribe Amygdaleae	Tribe Exochordeae	Tribe Spiraeeae	Tribe Maleae
				**InDel**			
Number of loci with considerable effects							
Total number	635	255	402	161	94	72	115
Number of high level	338	151	196	78	48	31	54
Number of low level	1	0	1	0	0	1	0
Number of moderate level	296	104	205	83	46	40	61
				**SNP**			
Number of loci with considerable effects							
Total number	9290	4737	6057	1806	2378	1890	2328
Number of high level	59	31	35	8	12	8	13
Number of low level	5418	2882	3464	1003	1418	1093	1334
Number of moderate level	3813	1824	2558	795	948	789	981

Note: Taking *Prunus pseudocerasus* (NC030599.1) as the referencing genome.

Supplementary Table 14 Estimation of positive selection of plastid protein-coding genes in Rosaceae and *Cerasus* (true cherry)

No.	Genes	M1a vs M2a in Rosaceae			M1a vs M2a in *Cerasus*		
		Site number with ω>1	P values in LRT	P values in BEB (site number)	Site number with ω>1	P values in LRT	P values in BEB (site number)
1	***accD***	**7**	**0.0001**	**>0.950 (3)**<br><0.950 (4)	1	NS	0.693 (1)
2	*atpA*	12	NS	<0.950 (12)	3	NS	<0.950 (3)
3	*atpB*	3	NS	<0.950 (3)	2	NS	<0.950 (2)
4	*atpE*	0	-	-	1	NS	0.738 (1)
5	*atpF*	1	NS	0.552 (1)	0	-	-
6	*atpH*	0	-	-	1	NS	0.798 (1)
7	*atpI*	0	-	-	0	-	-
8	*ccsA*	2	NS	<0.950 (2)	0	-	-
9	*cemA*	3	NS	<0.950 (3)	1	NS	0.786 (1)
10	*clpP*	4	NS	<0.950 (4)	0	-	-
11	***matK***	27	NS	<0.950 (27)	**7**	**0.0000**	**>0.950 (2)**<br><0.950 (5)
12	*ndhA*	1	NS	0.517 (1)	0	-	-
13	*ndhB*	5	NS	<0.950 (5)	ND	-	-
14	*ndhC*	2	NS	<0.950 (2)	ND	-	-
15	***ndhD***	**9**	**0.0049**	**>0.950 (1)**<br><0.950 (8)	ND	-	-
16	*ndhE*	1	NS	0.784 (1)	ND	-	-
17	***ndhF***	**16**	**0.0000**	**>0.950 (2)**<br><0.950 (14)	1	NS	0.728 (1)
18	*ndhG*	0	-	-	ND	-	-
19	*ndhH*	3	NS	<0.950 (3)	0	-	-
20	*ndhI*	1	NS	0.682 (1)	ND	-	-
21	*ndhJ*	1	NS	0.757 (1)	0	-	-
22	*ndhK*	2	NS	<0.950 (2)	0	-	-
23	*petA*	4	NS	<0.950 (4)	0	-	-
24	*petB*	1	NS	0.938(1)	1	0.0246	0.873 (1)
25	*petD*	0	-	-	0	-	-
26	*petG*	0	-	-	ND	-	-
27	*petL*	0	-	-	ND	-	-
28	*petN*	0	-	-	ND	-	-
29	***psaA***	**5**	**0.0008**	**>0.950 (2)**<br><0.950 (3)	1	0.0039	0.916 (1)
30	*psaB*	3	NS	<0.950 (3)	0	-	-
31	*psaC*	0	-	-	ND	-	-
32	*psaI*	1	NS	0.552 (1)	ND	-	-
33	*psaJ*	0	-	-	0	-	-

(Continued)

34	*psbA*	0	-	-	ND	-	-
35	*psbB*	4	NS	<0.950 (4)	ND	-	-
36	*psbC*	2	NS	<0.950 (2)	0	-	-
37	*psbD*	0	-	-	ND	-	-
38	*psbE*	0	-	-	ND	-	-
39	*psbF*	1	NS	0.709(1)	ND	-	-
40	*psbH*	0	-	-	ND	-	-
41	*psbI*	0	-	-	ND	-	-
42	*psbJ*	0	-	-	0	-	-
43	*psbK*	0	-	-	ND	-	-
44	***psbL***	**1**	**0.0313**	**0.956 (1)**	ND	-	-
45	*psbM*	0	-	-	ND	-	-
46	*psbN*	0	-	-	ND	-	-
47	*psbT*	0	-	-	ND	-	-
48	*psbZ*	0	-	-	ND	-	-
49	***rbcL***	**18**	**0.0000**	**>0.950 (6)**	ND	-	-
				<0.950 (12)			
50	*rpl2*	0	-	-	0	-	-
51	*rpl14*	0	-	-	0	-	-
52	*rpl16*	0	-	-	ND	-	-
53	*rpl20*	5	NS	<0.950 (5)	0	-	-
54	*rpl22*	8	NS	<0.950 (8)	0	-	-
55	*rpl23*	0	-	-	ND	-	-
56	*rpl32*	0	-	-	ND	-	-
57	*rpl33*	1	NS	0.594 (1)	ND	-	-
58	*rpl36*	0	-	-	ND	-	-
59	***rpoA***	**4**	**0.0493**	**>0.950 (1)**	0	-	-
				<0.950 (3)			
60	*rpoB*	7	NS	<0.950 (7)	0	-	-
61	*rpoC1*	5	NS	<0.950 (5)	2	NS	<0.950 (2)
62	*rpoC2*	5	NS	<0.950 (5)	1	NS	0.687 (1)
63	*rps2*	1	NS	0.550 (1)	0	-	-
64	*rps3*	4	NS	<0.950 (4)	ND	-	-
65	*rps4*	3	NS	<0.950 (3)	ND	-	-
66	*rps7*	0	-	-	ND	-	-
67	*rps8*	2	NS	<0.950 (2)	ND	-	-
68	*rps11*	0	-	-	ND	-	-
69	*rps14*	0	-	-	ND	-	-
70	*rps15*	0	-	-	0	-	-
71	***rps16***	2	**0.0470**	**>0.950 (1)**	ND	-	-
				<0.950 (1)			

(Continued)

72	***rps18***	**5**	**0.0000**	**>0.950 (3)**	ND	-	-
				<0.950 (2)			
73	*rps19*	1	NS	0.690 (1)	ND	-	-
74	***ycf1***	**35**	**0.0000**	**>0.950 (3)**	**22**	**0.0000**	**>0.950 (3)**
				<0.950 (32)			<0.950 (19)
75	*ycf1-fragment*	5	NS	>0.950 (1)	ND	-	-
				<0.950 (4)			
76	***ycf2***	**17**	**0.0001**	**>0.950 (1)**	ND	-	-
				<0.950 (16)			
77	*ycf3*	0	-	-	ND	-	-
78	*ycf4*	1	NS	0.591 (1)	ND	-	-

Note: NS: not significant (P>0.05); ND: not analyzed in true cherries due to no or low variations within gene.

LRT: Likelihood Ratio Test; BEB: Bayes empirical Bayes.

Supplementary Table 15 Test for the positive selection of protein-coding genes across Rosaceae plastomes

Genes	P values in LRT	ϖ from BEB	Amino acids	Taxonomic groups with positively selected genes				
				Subfamily Amygdaloideae				Subfamily Rosoideae
				Tribe Amygdaleae	Tribe Exochordeae	Tribe Spiraeeae	Tribe Maleae	
accD	0.0001	5.443±1.768	W	*Cer* (92), *Mic* (6), *Arm*, *Amy* (3), *Pad* (1), *Mad*	—	*Pen*	*Cha*, *Doc*, *Cyd*, *Sor*, *Mal* (2), *Pyr* (3), *Eri*	*Pot*, *Ros*
			R	*Pru*	—	—	—	—
			C	*Pad* (1)	—	—	—	—
			S	—	*Pri*	—	—	—
			Q	—	—	—	—	*Fra*
		5.394±1.822	L	*Cer* (90), *Mic* (5), *Arm*, *Pru*, *Amy* (3), *Pad* (1)	*Pri*	*Pen*	*Doc*, *Cyd*, *Sor*, *Pyr* (1), *Eri*	*Fra*, *Ros*
			F	*Cer* (2), *Mic* (1), *Pad* (1)	—	—	*Mal* (2), *Pyr* (1)	*Pot*
			S	*Mad*	—	—	*Cha*, *Pyr* (1)	—
		5.458±1.746	V	*Cer* (89), *Mic* (5), *Arm*, *Pru*, *Amy* (3), *Pad* (1), *Mad*	*Pri*	*Pen*	*Doc*, *Sor*, *Eri*	*Fra*
			M	*Cer* (3), *Mic* (1), *Pad* (1)	—	—	*Cyd*, *Mal* (2), *Pyr* (3)	*Pot*
			P	—	—	—	*Cha*	—
			A	—	—	—	—	*Ros*
*ndhD**	0.0049	3.086±0.974	I	*Cer* (92), *Mic* (6), *Pru*, *Amy* (3), *Pad* (2)	—	—	*Cyd*, *Sor*, *Mal* (2), *Pyr* (3), *Eri*	*Fra*
			T	*Arm*	—	—	*Cha*	*Ros*
			M	*Mad*	—	—	—	—
			V	—	*Pri*	*Pen*	—	—
			L	—	—	—	*Doc*	—
ndhF	0.0313	3.681±0.706	H	*Cer* (89), *Pad* (2), *Mad*	—	—	—	—
			N	*Cer* (3), *Mic* (6), *Arm*, *Pru*, *Amy* (3)	*Pri*	—	*Cha*, *Cyd*, *Sor*, *Mal* (2), *Pyr* (3)	*Fra*, *Pot*, *Ros*
			K	—	—	*Pen*	*Doc*, *Eri*	—
		3.630±0.799	N	*Cer* (91), *Mic* (6), *Arm*, *Pru*, *Amy* (1), *Pad* (2), *Mad*	—	—	—	—
			K	*Cer* (1), *Amy* (2)	*Pri*	*Pen*	—	*Fra*, *Pot*, *Ros*

(Continued)

			S	—	—	—	*Cha*, *Doc*, *Cyd*, *Sor*, *Mal* (2), *Pyr* (3), *Eri*	—
*psaA**	0.0008	3.535±1.290	N	*Cer* (61)	—	—	*Cyd*, *Sor*, *Pyr* (1)	*Fra*, *Ros*
			S	*Cer* (31), *Mic* (6), *Arm*, *Pru*, *Amy* (3), *Pad* (2), *Mad*	*Pri*,	*Pen*	*Cha*, *Doc*, *Mal* (2), *Pyr* (2), *Eri*	—
		3.540±1.285	G	—	—	—	*Cyd*, *Pyr* (3), *Sor*	*Fra*, *Ros*
			S	*Cer* (92), *Mic* (6), *Arm*, *Pru*, *Amy* (3), *Pad* (2), *Mad*	*Pri*	*Pen*	*Cha*, *Doc*, *Mal* (2), *Eri*	—
psbL	0.0313	7.125±2.745	T	*Cer* (92), *Mic* (6), *Pru*, *Amy* (3), *Pad* (2), *Mad*	*Pri*	—	*Cha*, *Doc*, *Cyd*, *Sor*, *Mal* (2), *Pyr* (3), *Eri*	—
			M	*Arm*	—	*Pen*	—	*Fra*, *Ros*, *Pot*
rbcL	0.0000	4.183±0.775	D	*Cer* (90), *Mic* (6), *Arm*, *Amy* (3), *Pad* (2), *Mad*	—	*Pen*	*Doc*, *Cyd*, *Sor*, *Mal* (2), *Pyr* (3), *Eri*	*Fra*, *Pot*
			E	*Cer* (2), *Pru*	*Pri*	—	*Cha*	*Ros*
		4.217±0.706	V	*Cer* (92)	—	—	*Pyr* (1)	—
			C	*Mic* (6), *Arm*, *Pru*, *Amy* (3), *Pad* (2), *Mad*	*Pri*	*Pen*	*Cha*, *Doc*, *Cyd*, *Sor*, *Mal* (2), *Pyr* (2), *Eri*	*Fra*, *Pot*, *Ros*
		4.200±0.740	E	*Cer* (92), *Mic* (2), *Arm*, *Pru*, *Amy* (3), *Pad* (2), *Mad*	*Pri*	*Pen*	*Doc*, *Eri*	*Pot*
			D	*Mic* (4)	—	—	*Cha*, *Cyd*, *Sor*, *Mal* (2), *Pyr* (3)	*Fra*, *Ros*
		4.166±0.807	A	*Cer* (92), *Pru*	—	—	—	—
			V	*Mic* (4), *Amy* (3), *Pad* (2), *Mad*	*Pri*	*Pen*	*Cha*, *Sor*, *Mal* (2), *Pyr* (3), *Eri*	*Fra*, *Pot*, *Ros*
			I	*Mic* (2), *Arm*	—	—	*Doc*, *Cyd*	—
		4.239±0.653	T	*Cer* (92), *Pru*	—	—	*Cha*, *Cyd*, *Sor*, *Mal* (2), *Pyr* (3)	*Fra*, *Ros*
			S	*Mic* (6), *Arm*, *Amy* (3), *Pad* (2), *Mad*	*Pri*	*Pen*	*Doc*, *Eri*	*Pot*
		4.199±0.747	M	*Cer* (92), *Mic* (5), *Pru*, *Amy* (1)	—	—	—	—
			L	*Mic* (1), *Arm*, *Amy* (2), *Pad* (2), *Mad*	*Pri*	*Pen*	*Cha*, *Doc*, *Cyd*, *Sor*, *Mal* (2), *Pyr* (3), *Eri*	*Fra*, *Pot*, *Ros*
rpoA	0.0493	4.127±2.108	F	*Cer* (92), *Mic* (6), *Arm*, *Pru*, *Amy* (3)	—	—	—	—
			L	*Pad* (2), *Mad*	—	*Pen*	*Cha*, *Doc*, *Cyd*, *Sor*, *Mal* (2), *Pyr* (3), *Eri*	*Pot*
			P	—	*Pri*	—	—	*Fra*, *Ros*
rps16	0.0470	6.453±2.445	R	*Cer* (92), *Mic* (6), *Pru*, *Amy* (3), *Pad* (2), *Mad*	—	*Pen*	*Cha*, *Doc*, *Cyd*, *Sor*, *Mal* (2), *Pyr* (3), *Eri*	*Ros*, *Pot*
			H	*Arm*	—	—	—	*Fra*
			N	—	*Pri*	—	—	—

(Continued)

rps18	0.0000	9.082±1.460	R	*Cer* (92), *Mic* (6), *Arm*, *Pru*, *Amy* (3)	*Pri*	—	—	—
			A	*Pad* (2), *Mad*	—	*Pen*	*Cha*, *Doc*, *Cyd*, *Sor*, *Mal* (2), *Pyr* (3), *Eri*	*Fra*, *Ros*
			T	—	—	—	—	*Pot*
		8.982±1.703	K	*Cer* (92), *Mic* (6), *Arm*, *Pru*, *Amy* (3)	*Pri*	—	—	—
			T	*Pad* (2), *Mad*	—	*Pen*	—	*Fra*, *Pot*, *Ros*
			A	—	—	—	*Cha*, *Doc*, *Cyd*, *Sor*, *Mal* (2), *Pyr* (3), *Eri*	—
		9.087±1.446	K	*Cer* (92), *Mic* (6), *Arm*, *Pru*, *Amy* (3)	—	—	—	—
			A	*Pad* (2), *Mad*	—	*Pen*	*Cha*, *Doc*, *Cyd*, *Sor*, *Mal* (2), *Pyr* (3), *Eri*	—
			E	—	*Pri*	—	—	—
			P	—	—	—	—	*Fra*, *Pot*
			T	—	—	—	—	*Ros*
*ycf1**	0.0000	3.416±0.493	L	*Cer* (89), *Mic* (5), *Arm*, *Pru*, *Amy* (3), *Pad* (1)	*Pri*	—	*Doc*, *Mal* (2), *Pyr* (2), *Eri*	—
			F	*Cer* (2), *Pad* (1), *Mad*	—	*Pen*	—	*Fra*, *Pot*, *Ros*
			I	—	—	—	*Sor*	—
			V	—	—	—	*Pyr* (1)	—
		3.391±0.548	Q	*Cer* (91), *Mic* (5), *Arm*, *Pru*, *Amy* (3), *Pad* (2), *Mad*	—	—	*Doc*, *Sor*, *Mal* (2), *Pyr* (3), *Eri*	*Fra*
			G	—	*Pri*	—	—	—
			R	—	—	*Pen*	—	*Ros*
			E	—	—	—	—	*Pot*
		3.463±0.359	T	*Cer* (87), *Mic* (5), *Arm*, *Pru*, *Amy* (3)	—	—	—	—
			G	*Cer* (1), *Pad* (2), *Mad*	—	—	—	—
			N	*Cer* (3)	—	—	—	—
			I	—	*Pri*	—	—	—
			R	—	—	*Pen*	—	*Ros*
			P	—	—	—	*Doc*, *Sor*, *Mal* (2), *Pyr* (3), *Eri*	—
			S	—	—	—	—	*Fra*

(Continued)

			K	—	—	—	—	*Pot*
*ycf2**	0.0063	5.033±1.798	D	*Cer* (92), *Mic* (6), *Arm*, *Pru*, *Amy* (3), *Pad* (2)	*Pri*	*Pen*	*Cha*, *Doc*, *Cyd*, *Sor*, *Mal* (2), *Pyr* (3), *Eri*	*Pot*
			G	—	—	—	—	*Fra*
			I	—	—	—	—	*Ros*

Note: *Cer*: *Cerasus* (true cherry); *Mic*: *Microcerasus* (dwarf cherry); *Arm*: *Armeniaca*; *Pru*: *Prunus* (*Prunus cerasifera*); *Amy*: *Amygdalus*; *Pad*: *Padus*; *Mad*: *Maddenia*; *Pri*: *Prinsepia*; *Pen*: *Pentactina*; *Cha*: *Chaenomeles*; *Doc*: *Docynia*; *Cyd*: *Cydonia*; *Sor*: *Sorbus*; *Mal*: *Malus*; *Pyr*: *Pyrus*; *Eri*: *Eriobotrya*; *Fra*: *Fragaria*; *Pot*: *Potentilla*; *Ros*: *Rosa*. Numeral in bracket represented sample number. *: We removed *Potentilla micrantha* for *ndhD* and *psaA*, two *Cerasus* taxa and two tribe Maleae taxa for *ycf1* and *Prunus hypoleuca* for *ycf2* due to the remarkably short gene lengths in these taxa. LRT: Likelihood Ratio Test; BEB: Bayes empirical Bayes. The unique positively selected sites in three subfamily Rosoideae species, *Prinsepia utilis* and *Pentactina rupicola* were marked with light grey, and those in tribes Amygdaleae and Maleae taxa were marked with deep grey.

Supplementary Table 16 Test for the positive selection of protein-coding genes in true cherries (*Cerasus*)

Genes	P values in LRT	ϖ from BEB	Amino acids	Sample number of taxa (sample number)
matK	0.0000	9.220±1.393	Q	24 (57)
			K	1 (1)
			P	9 (34)
		9.215±1.407	K	24 (58)
			P	2 (2)
			Q	7 (32)
ycf1	0.0000	9.699±1.064	L	26 (87)
			F	3 (4)
		9.704±1.040	T	24 (87)
			G	1 (1)
			N	3 (3)
		9.694±1.080	F	24 (81)
			L	7 (10)

Note: LRT: Likelihood Ratio Test; BEB: Bayes empirical Bayes.

Supplementary Table 17 Characteristics of plastome sequences and best-fit models of nucleotide substitution for 12 datasets

Datasets	Sample size	Number of sites	Variable sites (%)	Parsimony-informative sites (%)	Best-fit models
WCGD	124	178338	37146 (20.83)	17432 (9.77)	GTR+I+G
PCGD	107	162019	9679 (5.97)	2864 (1.77)	GTR+I+G
WOID	124	151070	35404 (23.44)	16783 (11.11)	GTR+I+G
POID	107	135285	9307 (6.88)	2757 (2.04)	GTR+I+G
PWGD	124	126408	21459 (16.98)	10387 (8.22)	GTR+I+G
PPGD	107	147293	8303 (5.64)	2403 (1.63)	GTR+I+G
VSWD	124	23360	23360 (100)	11348 (48.58)	GTR
VSPD	107	8515	8515 (100)	2478 (29.10)	GTR
WGSD	124	73954	12749 (17.24)	6068 (8.21)	GTR+I+G
PGSD	107	71558	3416 (4.77)	1031 (1.44)	GTR+I+G
PCWD	124	52219	9164 (17.55)	4482 (8.58)	GTR+I+G
PCPD	107	51635	2520 (4.88)	763 (1.48)	GTR+I+G

Note: WCGD, whole plastomes dataset; PCGD, Amygdaleae plastomes dataset; WOID, whole one inverted-repeat dataset; POID, Amygdaleae one inverted-repeat dataset; PWGD, pruned whole plastomes dataset; PPGD, pruned Amygdaleae plastomes dataset; VSWD, variant sites of whole plastomes dataset; VSPD, variant sites of Amygdaleae plastomes dataset; WGSD, whole gene sequence dataset; PGSD, Amygdaleae gene sequence dataset; PCWD, protein-coding sequence of whole plastomes dataset; PCPD, protein-coding sequence of Amygdaleae plastomes dataset. The twelve datasets used to construct the phylogenetic trees and dataset used to estimate the divergence time were uploaded to Dryad (datadryad.org) (doi: 10.5061/dryad.jq2bvq88d).

Supplementary Table 18 Proportion of mutation events within and among tribe Amygdaleae and tribe Maleae of the subfamily Amygdaloideae

Groups	Mean values	Median values	Min values	Max
Within *Cerasus*	0.135	0.139	0.000	0.508
Within *Microcerasus*	0.176	0.233	0.004	0.317
Within *Amygdalus*	0.306	0.421	0.072	0.424
Within *Padus*	0.380	0.380	0.380	0.380
Between *Cerasus* and *Microcerasus*	0.611	0.605	0.529	0.851
Between *Cerasus* and *Prunus*	0.673	0.675	0.617	0.879
Between *Cerasus* and *Amygdalus*	0.720	0.717	0.622	0.953
Between *Cerasus* and *Armeniaca*	0.749	0.745	0.702	0.945
Between *Microcerasus* and *Prunus*	0.370	0.361	0.346	0.427
Between *Microcerasus* and *Amygdalus*	0.412	0.418	0.352	0.480
Between *Microcerasus* and *Armeniaca*	0.398	0.390	0.371	0.453
Between *Prunus* and *Amygdalus*	0.476	0.489	0.444	0.497
Between *Prunus* and *Armeniaca*	0.516	0.516	0.516	0.516
Between *Amygdalus* and *Armeniaca*	0.545	0.558	0.506	0.571
Between *Maddenia* and *Padus*[1]	0.444	0.444	0.433	0.455
Between *Cerasus* and *Maddenia*	1.129	1.121	1.083	1.344
Between *Microcerasus* and *Maddenia*	1.089	1.090	1.052	1.140
Between *Prunus* and *Maddenia*	1.171	1.171	1.171	1.171
Between *Amygdalus* and *Maddenia*	1.252	1.248	1.212	1.297
Between *Armeniaca* and *Maddenia*	1.287	1.287	1.287	1.287
Between *Cerasus* and *Padus*	1.133	1.125	1.041	1.396
Between *Microcerasus* and *Padus*	1.051	1.064	0.980	1.149
Between *Prunus* and *Padus*	1.168	1.168	1.143	1.194
Between *Amygdalus* and *Padus*	1.184	1.173	1.121	1.243
Between *Armeniaca* and *Padus*	1.192	1.192	1.132	1.251
Within *Pyrus*	0.097	0.145	0.001	0.146
Within *Malus*	0.193	0.193	0.193	0.193
Between *Pyrus* and *Malus*	0.572	0.575	0.546	0.606
Among different genera of the tribe Maleae	0.568	0.549	0.428	0.790

Note: [1]: *Padus* included *Prunus padus* and *Prunus serotina* in this study.

Supplementary Table 19 Genetic distances and genetic differentiation (F_{st}) among *Cerasus*, *Microcerasus* and their close relatives

Taxa	*Cerasus* (n=92)	*Microcerasus* (n=6)	*Amygdalus* (n=3)	*Prunus mume*	*Prunus cerasifera*
Cerasus (n=92)	-	0.005	0.006	0.006	0.005
Microcerasus (n=6)	0.786	-	0.003	0.003	0.003
Amygdalus (n=3)	0.729	0.436	-	0.004	0.004
Prunus mume	NA	NA	NA	-	0.004
Prunus cerasifera	NA	NA	NA	NA	-

Note: upper: genetic distance; lower: genetic differentiation; NA: not available. The values of genetic distance and differentiation between *Maddenia* and *Padus* were 0.004 and NA, and those between *Malus* and *Pyrus* were 0.003 and 0.790, respectively. n: sample number.

Supplementary Table 20 The number of shared InDel and SNP mutations among *Cerasus*, *Microcerasus* and their close relatives

Taxa	*Cerasus* (n=92)	*Microcerasus* (n=6)	*Amygdalus* (n=3)	*Prunus mume*	*Prunus cerasifera*
Cerasus (n=92)	-	129	107	72	61
Microcerasus (n=6)	241	-	197	159	162
Amygdalus (n=3)	250	614	-	144	136
Prunus mume	208	604	580	-	104
Prunus cerasifera	190	523	528	501	-

Note: upper: InDel; lower: SNP. n: sample number.

Supplementary Table 21 The mean values of similarity coefficients among *Cerasus*, *Microcerasus* and their close relatives

Taxa	*Cerasus* (n=92)	*Microcerasus* (n=6)	*Amygdalus* (n=3)	*Prunus mume*	*Prunus cerasifera*
Cerasus (n=92)	-				
Microcerasus (n=6)	0.985	-			
Amygdalus (n=3)	0.984	0.988	-		
Prunus mume	0.984	0.990	0.989	-	
Prunus cerasifera	0.985	0.990	0.988	0.988	-

Note: The mean values of similarity coefficients between *Maddenia* and *Padus* was 0.982, and that between *Malus* and *Pyrus* was 0.983, respectively. n: sample number.

Supplementary Table 22 Genetic distances and genetic differentiation (F_{st}) among fruiting cherry species

Species	***P. pseudocerasus* (n=35)**	***P. avium* (n=3)**	***P. cerasus* × *P. canescens* (n=1)**	***P. fruticosa* (n=1)**	***P. mahaleb* (n=1)**	***P. tomentosa* (n=5)**
Prunus pseudocerasus (n=35)	-	0.002	0.003	0.003	0.002	0.005
P. avium (n=3)	0.728	-	0.003	0.003	0.002	0.005
P. cerasus × *P. canescens* (n=1)	NA	NA	-	0.000	0.003	0.006
P. fruticosa (n=1)	NA	NA	NA	-	0.003	0.006
P. mahaleb (n=1)	NA	NA	NA	NA	-	0.005
P. tomentosa (n=5)	0.924	0.946	NA	NA	NA	NA

Note: upper: genetic distance; lower: genetic differentiation; NA: not available. n: sample number.

Supplementary Table 23 The mean values of proportion of mutation events and similarity coefficients among fruiting cherry species

Species	***P. pseudocerasus* (n=35)**	***P. avium* (n=3)**	***P. cerasus* × *P. canescens* (n=1)**	***P. fruticosa* (n=1)**	***P. mahaleb* (n=1)**	***P. tomentosa* (n=5)**
Prunus pseudocerasus (n=35)	-	0.234	0.383	0.362	0.278	0.590
P. avium (n=3)	0.995	-	0.476	0.433	0.350	0.645
P. cerasus × *P. canescens* (n=1)	0.991	0.989	-	**0.021**	0.508	0.784
P. fruticosa (n=1)	0.991	0.989	**0.999**	-	0.458	0.822
P. mahaleb (n=1)	0.993	0.992	0.988	0.988	-	0.615
P. tomentosa (n=5)	0.985	0.984	0.981	0.981	0.985	-

Note: upper: mean values of proportion of mutation events; lower: mean values of similarity coefficients. n: sample number.

Supplementary Table 24 Protein-coding genes with unique InDels and SNPs within *Prunus pseudocerasus* and within *P. avium*

Fruiting cherry	Genes with unique InDels	Genes with unique SNPs
Chinese cherry (*P. pseudocerasus*, n=35)	*atpF*, *clpP*, *ndhA*, ***ndhF***, ***ndhI***, *petB*, *petD*, ***psbL***, *rpl16*, *rpoC1*, ***rps15***, *rps16*, *trnK-UUU*, *trnG-UCC*, ***ycf1*-fragment**, *ycf3*	***accD***, ***atpA***, ***atpB***, ***atpE***, ***atpF***, ***atpH***, ***ccsA***, ***cemA***, ***clpP***, *infA*, ***ndhA***, ***ndhD***, ***ndhF***, ***ndhG***, ***ndhJ***, ***ndhK***, ***petA***, *petB*, ***petD***, ***psaB***, ***psbA***, ***psbC***, ***psbJ***, ***rpl14***, ***rpl16***, ***rpl20***, ***rpl22***, ***rpl36***, ***rpoB***, ***rpoC1***, ***rpoC2***, ***rps3***, ***rps11***, *rps16*, ***rps19*-fragment**, *rrn23*, *trnA-UGC*, *trnG-UCC*, *trnI-GAU*, *trnK-UUU*, *trnL-UAA*, ***ycf1***, ***ycf1*-fragment**, ***ycf2***, *ycf3*, ***ycf4***
European sweet cherry (*P. avium*, n=3)	*atpF*, *clpP*, *ndhA*, ***ndhF***, ***ndhI***, *petB*, ***psbL***, *rpl16*, *rpoC1*, *rps16*, *trnK-UUU*, ***ycf1*-fragment**, *ycf3*	***accD***, ***atpA***, ***atpB***, ***atpI***, ***ccsA***, ***cemA***, ***clpP***, ***matK***, ***ndhA***, ***ndhC***, ***ndhD***, ***ndhF***, ***ndhH***, ***ndhJ***, ***ndhK***, ***petA***, *petB*, ***psaA***, ***psbA***, ***psbB***, ***rpl14***, ***rpl33***, ***rpoA***, ***rpoB***, ***rpoC1***, ***rpoC2***, ***rps2***, ***rps3***, ***rps7***, ***rps15***, *rps16*, ***rps19*-fragment**, *rrn16*, *rrn23*, *trnA-UGC*, *trnG-UCC*, *trnH-GUG*, *trnK-UUU*, *trnL-UAA*, *trnV-UAC*, ***ycf1***, ***ycf2***, *ycf3*, ***ycf4***

Note: InDels and SNPs led to effective (high, moderate and low) impacts on 49 chloroplast protein-coding genes, which were bold above. n: sample number.

Supplementary Table 25 The number of shared InDel and SNP mutations among fruiting cherry species

**Species**	***P. pseudocerasus* (n=35)**	***P. avium* (n=3)**	***P. cerasus* × *P. canescens* (n=1)**	***P. fruticosa* (n=1)**	***P. mahaleb* (n=1)**	***P. tomentosa* (n=5)**
Prunus pseudocerasus (n=35)	-	54	28	23	42	38
P. avium (n=3)	105	-	24	19	30	21
P. cerasus × *P. canescens* (n=1)	102	101	-	**150**	17	20
P. fruticosa (n=1)	89	88	**389**	-	14	16
P. mahaleb (n=1)	125	98	103	92	-	33
P. tomentosa (n=5)	101	93	92	91	159	-

Note: upper: InDel; lower: SNP. n: sample number.

Supplementary Table 26 Comparison of morphological characteristics of true cherry, dwarf cherry, and close relatives

Morphological Features	True cherry (*Cerasus*)	Dwarf cherry (*Microcerasus*)	Close relatives		
			Subg. *Amygdalus*	Subg. *Armeniaca*	Subg. *Prunus*
**Growth habit**	**Trees**	**Shrubs or small trees**	Trees or shrubs	Trees, rarely shrubs	Trees or shrubs
**Bark**	**Many conspicuous lenticels**	**Rarely with lenticels**	Small lenticels	Sparse and pale lenticels	Inconspicuous lenticels
**Axillary winter buds**	**1 per leaf axil**	**3 per leaf axil with 2 lateral flower buds and 1 central leaf bud**	(2 or) 3, lateral ones flower buds, central one a leaf bud	Single	Single, ovoid
Terminal winter buds	Present	Present	Present	Absent	Absent
Leaf vernation	Conduplicate	Conduplicate	Conduplicate	Convolute	Convolute or conduplicate
Petiole	Long, 1-3 nectaries	Short, 2 nectaries	Short to long, 2 nectaries	Short to long, often 2 nectaries	Short to long, often 2 nectaries
**Inflorescence**	**Umbellate or corymbose-racemose**	**Solitary or 2 in a fascicle**	Axillary, 1(or 2)-flowered	Axillary, 1－3-flowered	Axillary, solitary or to 3-flowered in a fascicle
**Pedicel**	**Short to long**	**Sessile or short**	Nearly absent or short	Nearly absent to very short	Short
**Bract**	**A bract below each flower**	**No bract**	No bract	No bract	No bract
Fruit	Glabrous, not glaucous, without longitudinal groove	Glabrous, without longitudinal groove	Hairy, longitudinal groove	Hairy, longitudinal groove	Glabrous, glaucous, longitudinal groove
Endocarp	Smooth or rugose	Smooth	Furrowed	Smooth, scabrous, or reticulate	Smooth

Note: The morphological characteristics followed Flora of China (Yü et al. 1986).

Subfamily Amygdaloideae

Tribe Amygdaleae

Prunus subg. *Cerasus* (True cherry)

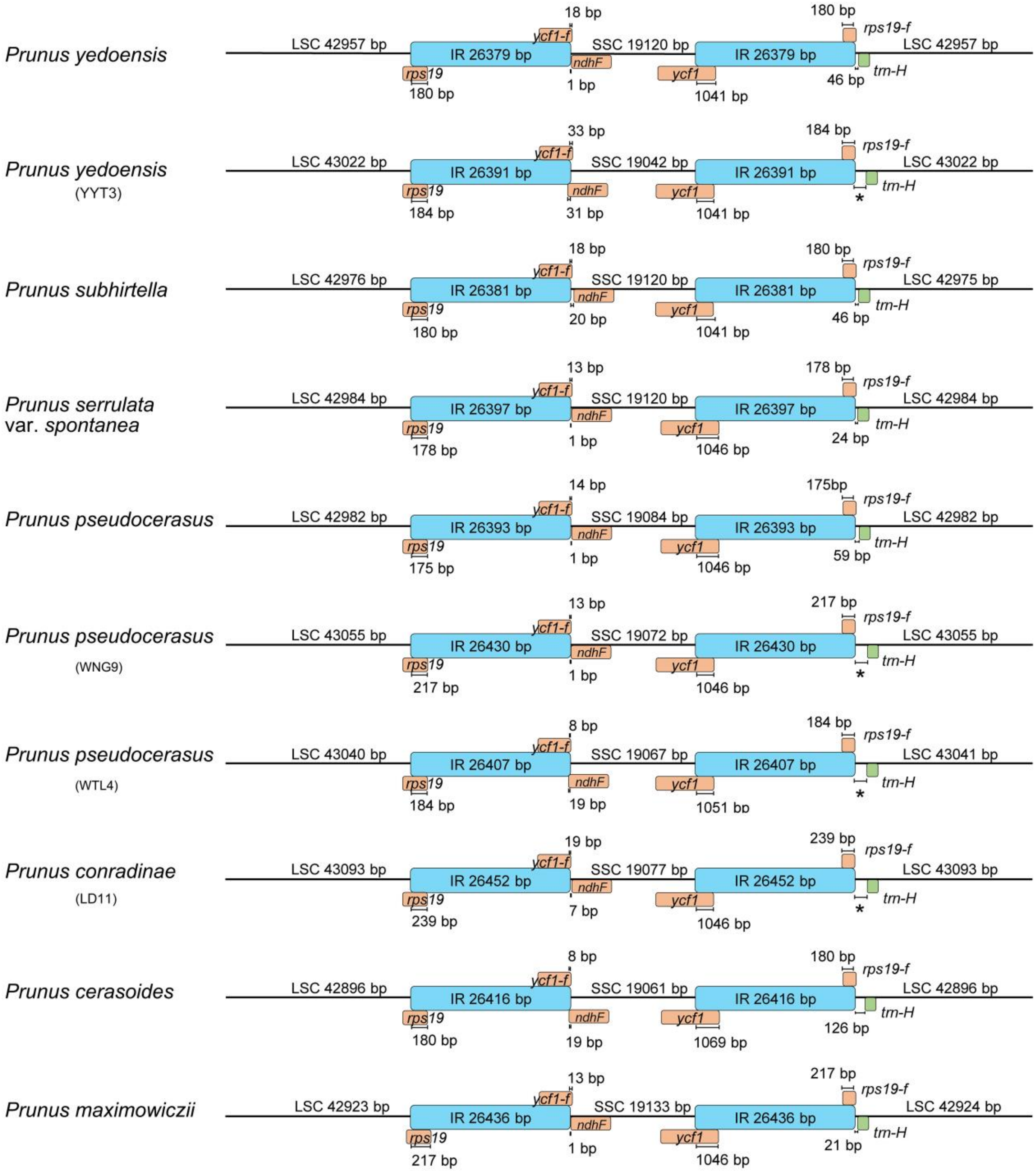

Prunus subg. *Prunus* (*Microcerasus*, Dwarf cherry)

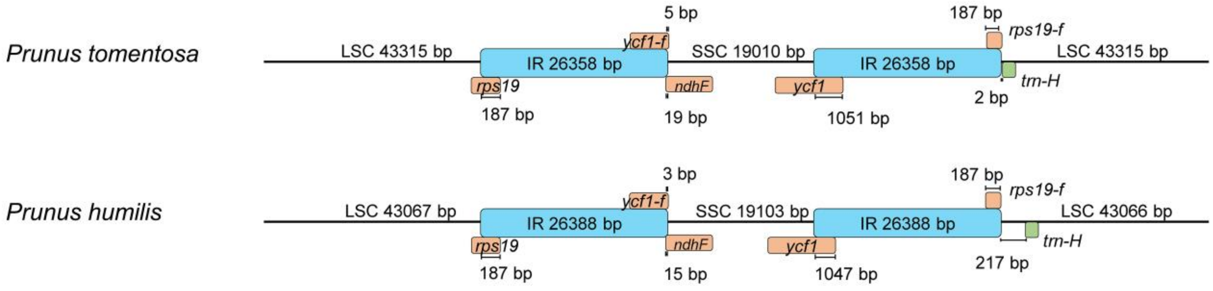

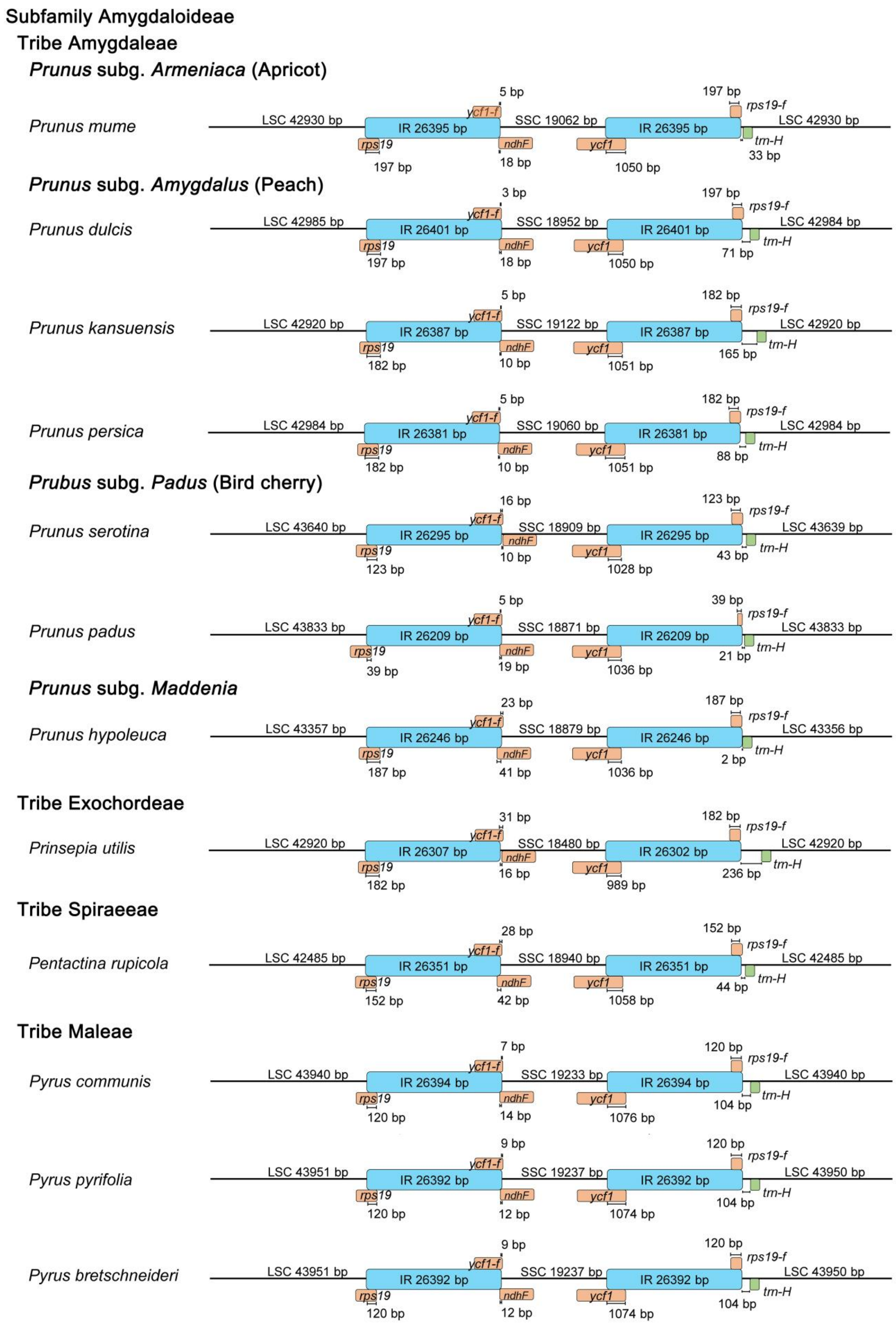
Subfamily Amygdaloideae
Tribe Amygdaleae
Prunus subg. Armeniaca (Apricot)
Prunus mume
LSC 42930 bp
IR 26395 bp
SSC 19062 bp
IR 26395 bp
LSC 42930 bp
5 bp
197 bp
ycf1-f
rps19-f
rps19
ndhF
ycf1
trn-H
197 bp
18 bp
1050 bp
33 bp
Prunus subg. Amygdalus (Peach)
Prunus dulcis
LSC 42985 bp
IR 26401 bp
SSC 18952 bp
IR 26401 bp
LSC 42984 bp
3 bp
197 bp
197 bp
18 bp
1050 bp
71 bp
Prunus kansuensis
LSC 42920 bp
IR 26387 bp
SSC 19122 bp
IR 26387 bp
LSC 42920 bp
5 bp
182 bp
182 bp
10 bp
1051 bp
165 bp
Prunus persica
LSC 42984 bp
IR 26381 bp
SSC 19060 bp
IR 26381 bp
LSC 42984 bp
5 bp
182 bp
182 bp
10 bp
1051 bp
88 bp
Prubus subg. Padus (Bird cherry)
Prunus serotina
LSC 43640 bp
IR 26295 bp
SSC 18909 bp
IR 26295 bp
LSC 43639 bp
16 bp
123 bp
123 bp
10 bp
1028 bp
43 bp
Prunus padus
LSC 43833 bp
IR 26209 bp
SSC 18871 bp
IR 26209 bp
LSC 43833 bp
5 bp
39 bp
39 bp
19 bp
1036 bp
21 bp
Prunus subg. Maddenia
Prunus hypoleuca
LSC 43357 bp
IR 26246 bp
SSC 18879 bp
IR 26246 bp
LSC 43356 bp
23 bp
187 bp
187 bp
41 bp
1036 bp
2 bp
Tribe Exochordeae
Prinsepia utilis
LSC 42920 bp
IR 26307 bp
SSC 18480 bp
IR 26302 bp
LSC 42920 bp
31 bp
182 bp
182 bp
16 bp
989 bp
236 bp
Tribe Spiraeeae
Pentactina rupicola
LSC 42485 bp
IR 26351 bp
SSC 18940 bp
IR 26351 bp
LSC 42485 bp
28 bp
152 bp
152 bp
42 bp
1058 bp
44 bp
Tribe Maleae
Pyrus communis
LSC 43940 bp
IR 26394 bp
SSC 19233 bp
IR 26394 bp
LSC 43940 bp
7 bp
120 bp
120 bp
14 bp
1076 bp
104 bp
Pyrus pyrifolia
LSC 43951 bp
IR 26392 bp
SSC 19237 bp
IR 26392 bp
LSC 43950 bp
9 bp
120 bp
120 bp
12 bp
1074 bp
104 bp
Pyrus bretschneideri
LSC 43951 bp
IR 26392 bp
SSC 19237 bp
IR 26392 bp
LSC 43950 bp
9 bp
120 bp
120 bp
12 bp
1074 bp
104 bp

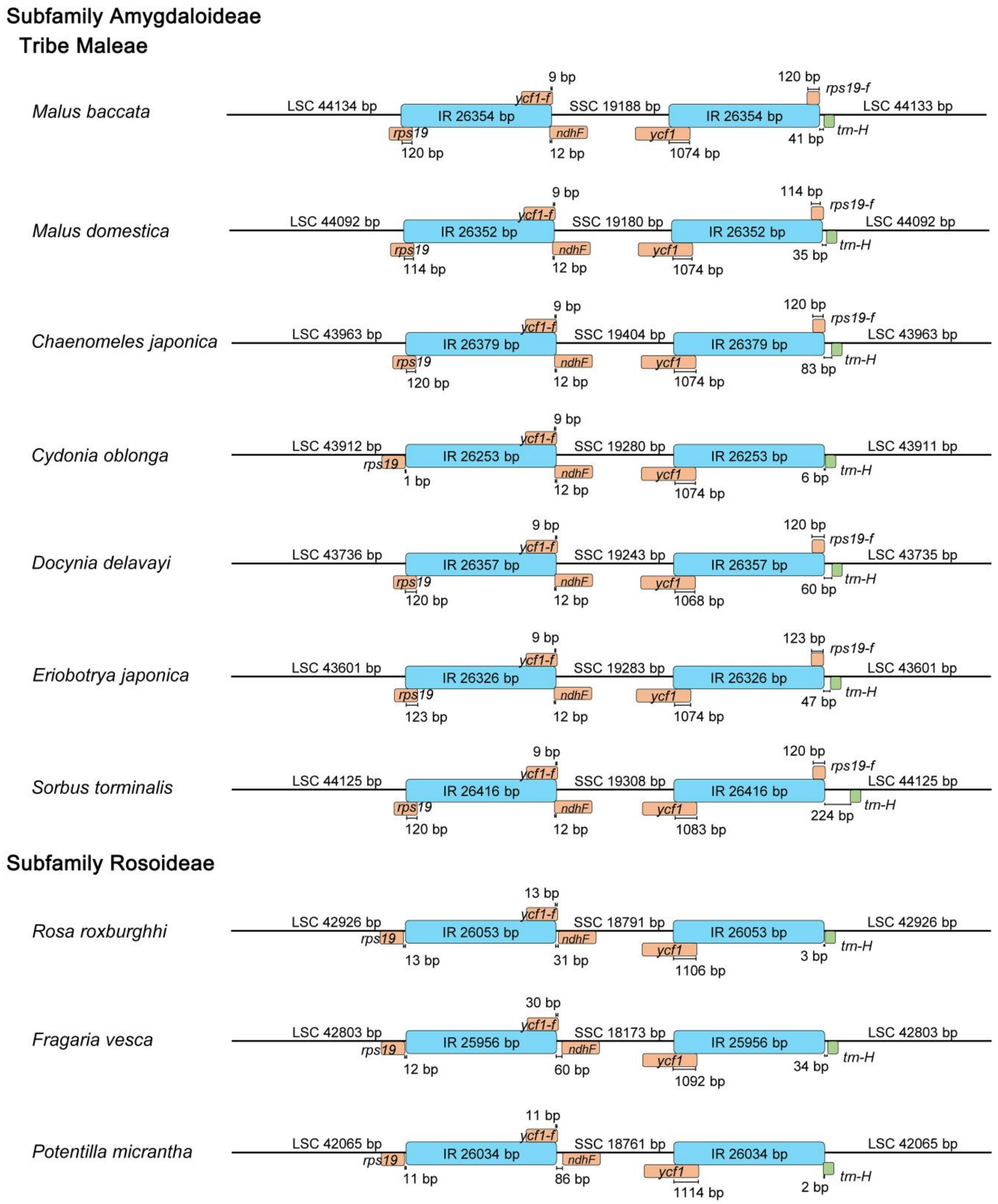

Supplementary Figure 1 The distribution and length of LSC, SSC, IRs and their neighboring genes in 34 representative Rosaceae plastomes. *: no exact value was available due to the missing data. LSC: long-single copy; SSC: short-single copy; IR: inverted repeat regions.

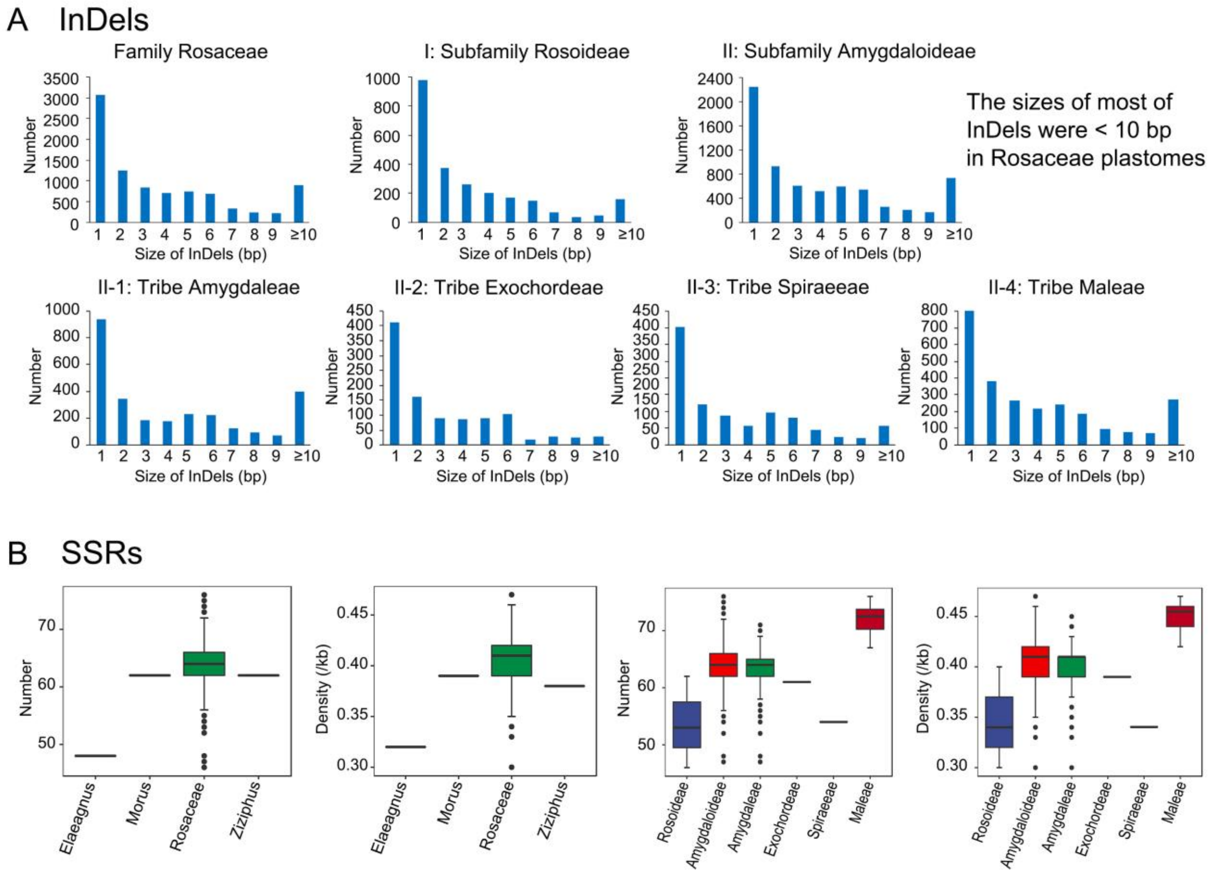

Supplementary Figure 2 The characteristics of InDels and SSRs across the Rosaceae plastomes. a. The number of insertion and deletion with different lengths. b. The number and density of SSRs at different taxonomic levels.

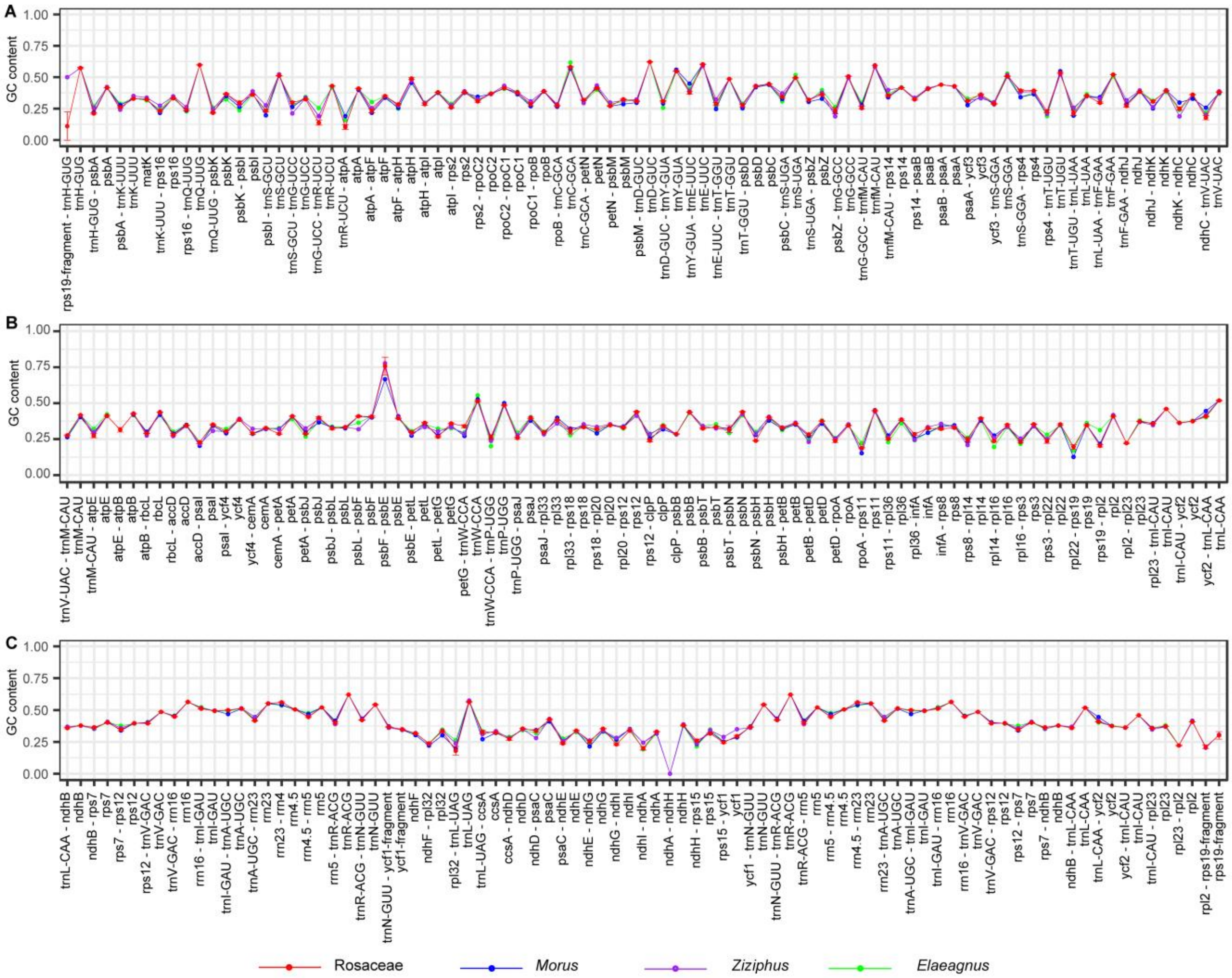

Supplementary Figure 3 GC contents within each inter- and intra-genic region among Rosaceae and outgroups. Black arrows indicated the high variations of GC content in Rosaceae family. Red arrows represented the regions with remarkedly high or low GC contents in Rosaceae and outgroups. The GC content of the intergenic region of *rps19*-fragment and *trnH-GUG* should be ignored due to the high missing data.

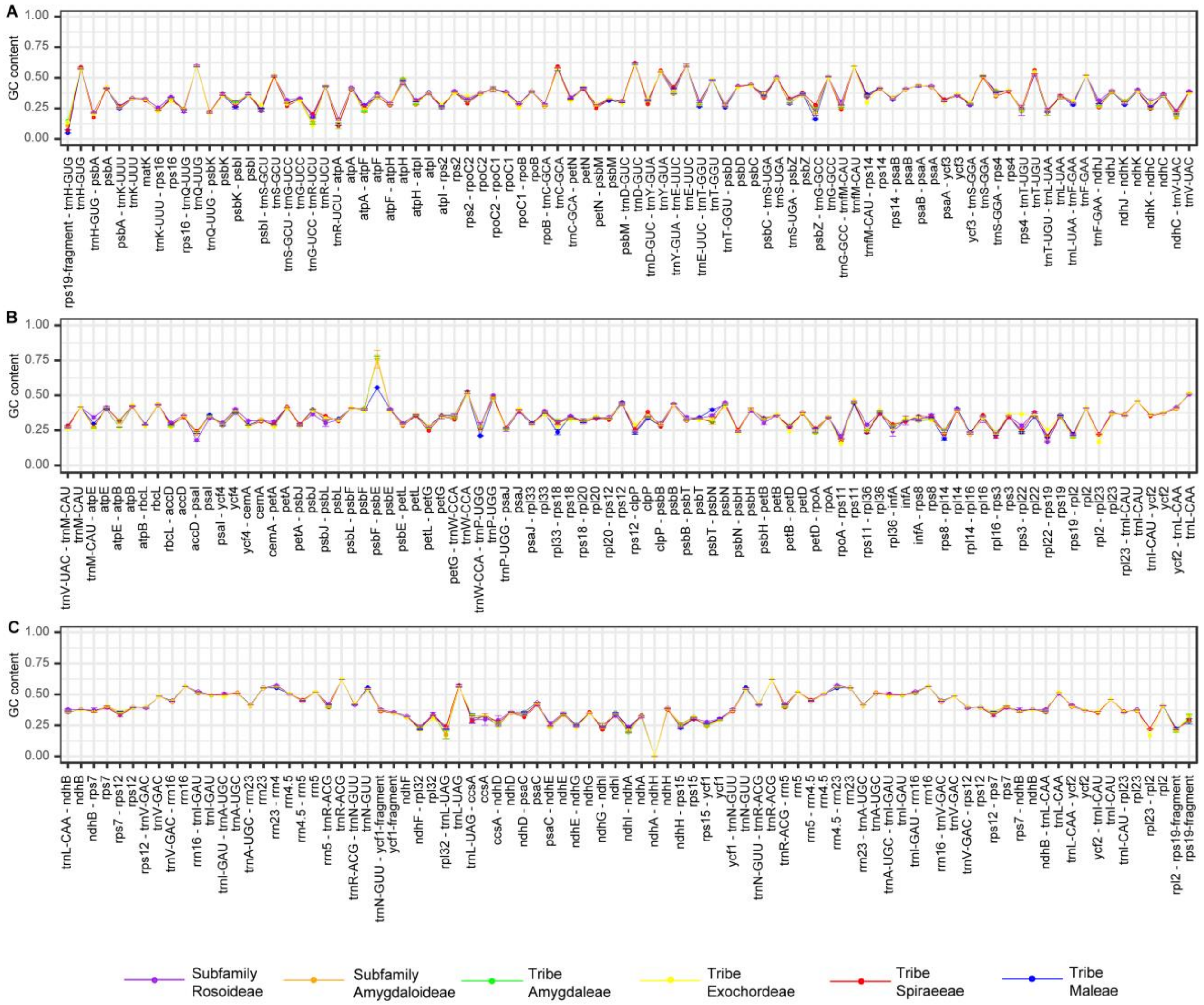

Supplementary Figure 4 GC contents within each inter- and intra-genic region between subfamilies Amygdaloideae and Rosoideae, and among tribes of subfamily Amygdaloideae. Black arrows indicated the high variations of GC content. Red arrows represented the regions with remarkedly high or low GC contents between subfamilies, and among tribes. The GC content of the intergeniHc region of *rps19*-fragment and *trnH-GUG* should be ignored due to the high missing data.

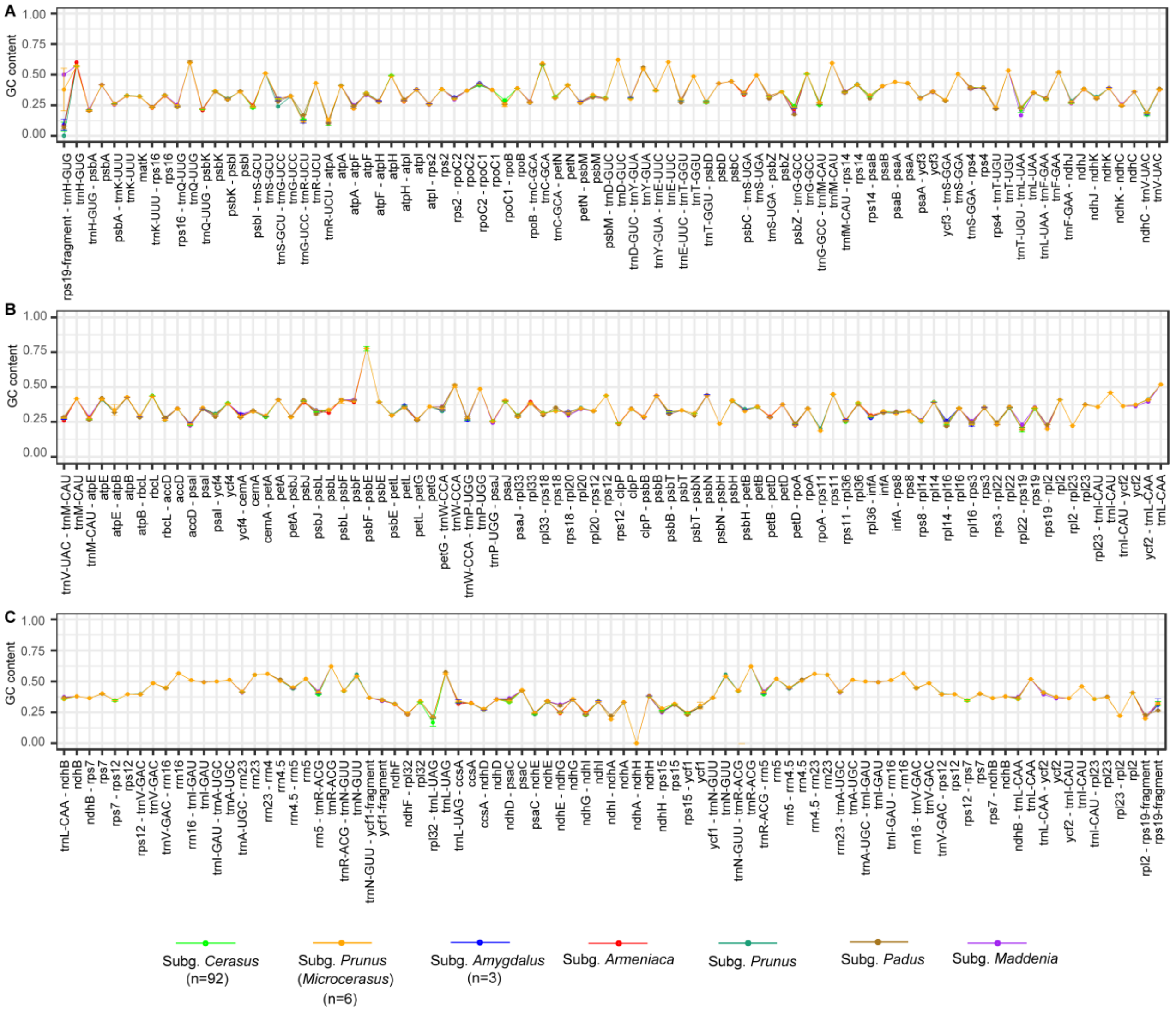

Supplementary Figure 5 GC contents within each inter- and intra-genic region among *Cerasus* and its relatives. Black arrows indicated the high variations of GC content between *Cerasus* and its relatives. Red arrows represented the regions with remarkedly high or low GC contents among subgenera. The GC content of the intergenic region of *rps19*-fragment and *trnH-GUG* should be ignored due to the high missing data.

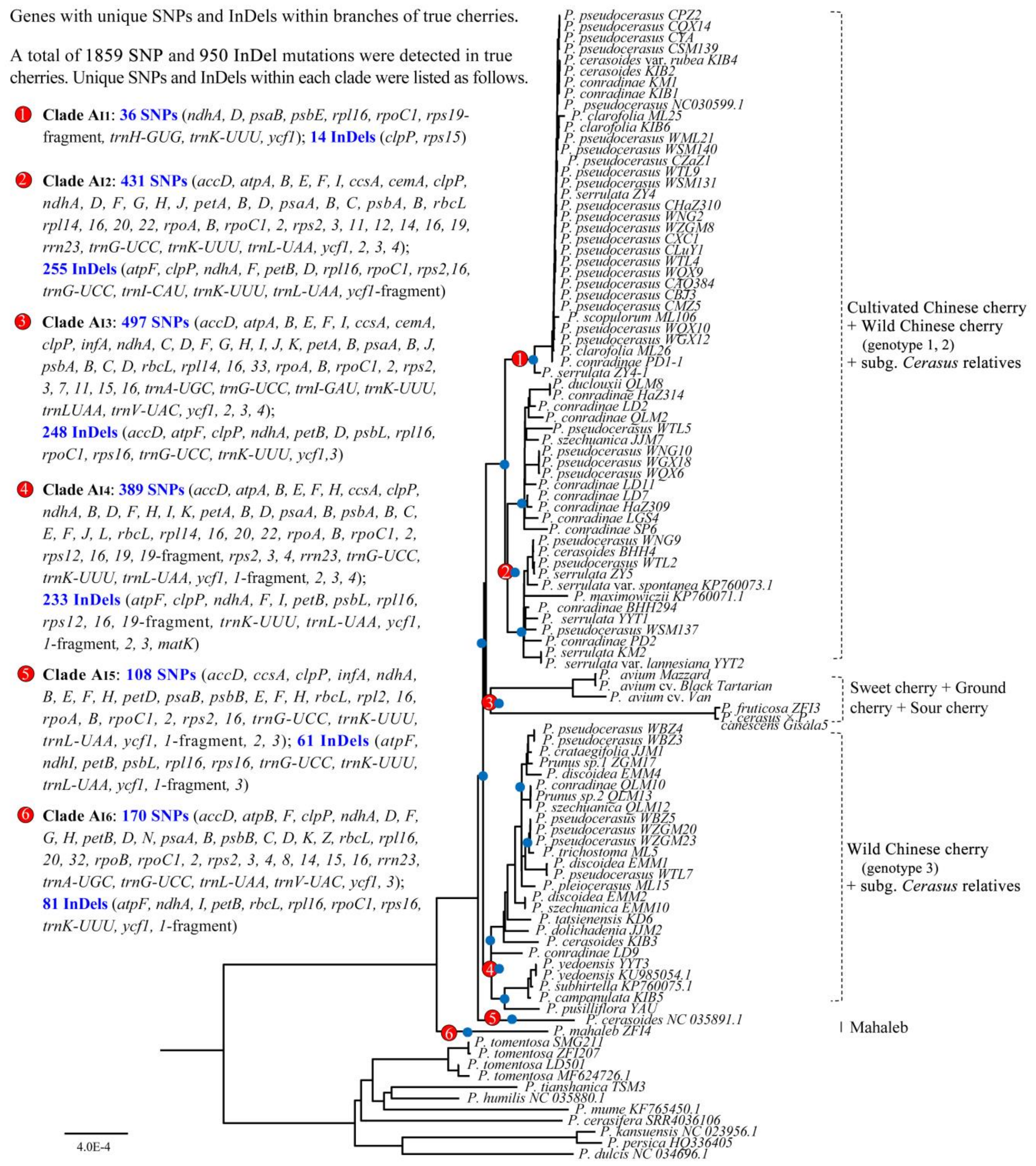

Supplementary Figure 6 Detected SNPs and InDels among true cherry. The six groups were corresponding to the phylogenetic tree in Fig. 3. Unique SNPs and InDels were detected and showed for each group. Plastid genes harboring SNPs and InDels were also exhibited here. No chloroplast gene underwent significantly positive selection within 15 nodes (blue dots) according to PAML analyses based on Branch-site model.

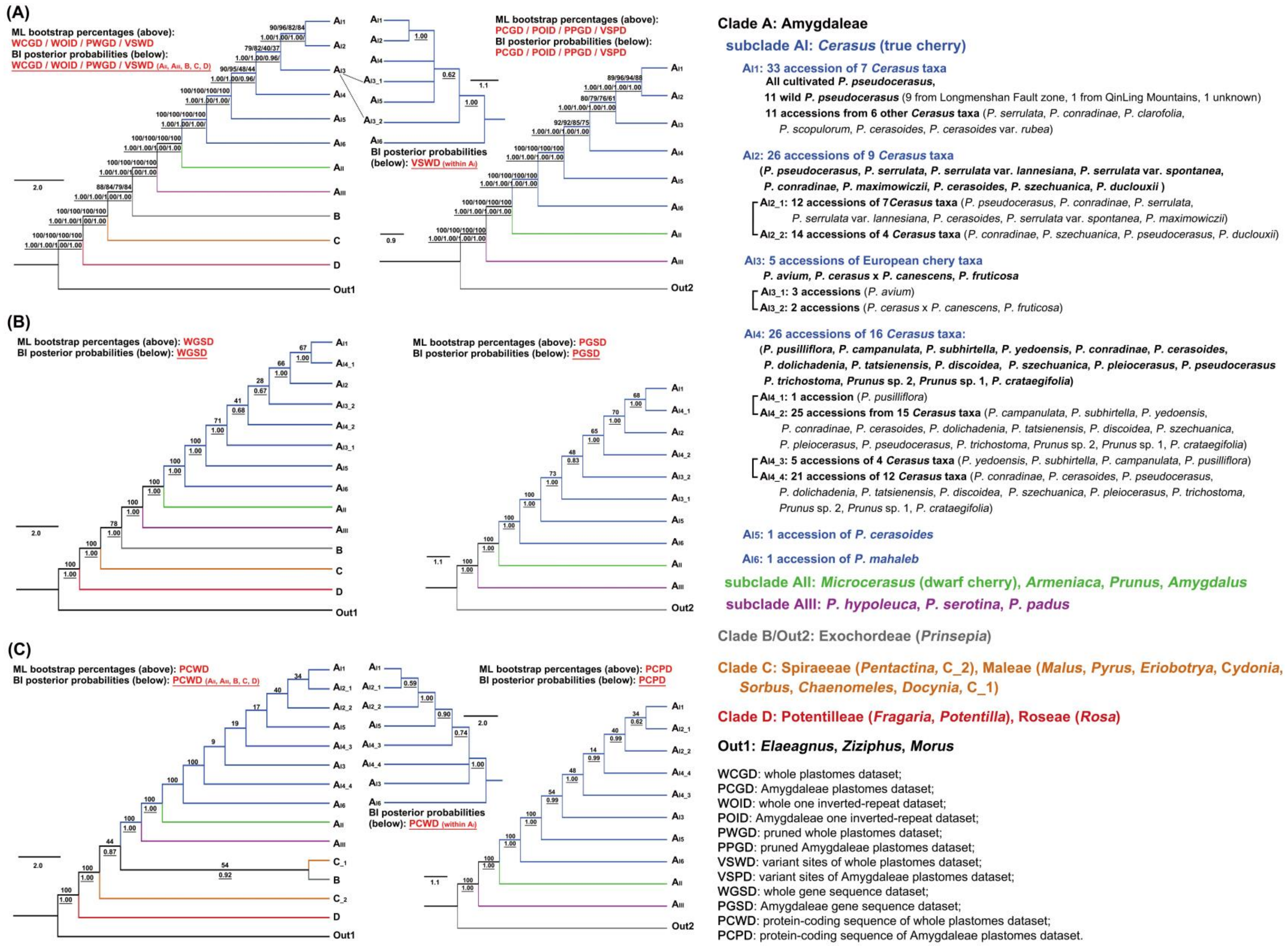

Supplementary Figure 7 The summary of different major clades generated by different data and methods in this study. The maximum likelihood (ML) bootstrap value and Bayesian inference (BI) posterior probabilities were shown. The numbers of accessions and taxa were listed within each major clade.

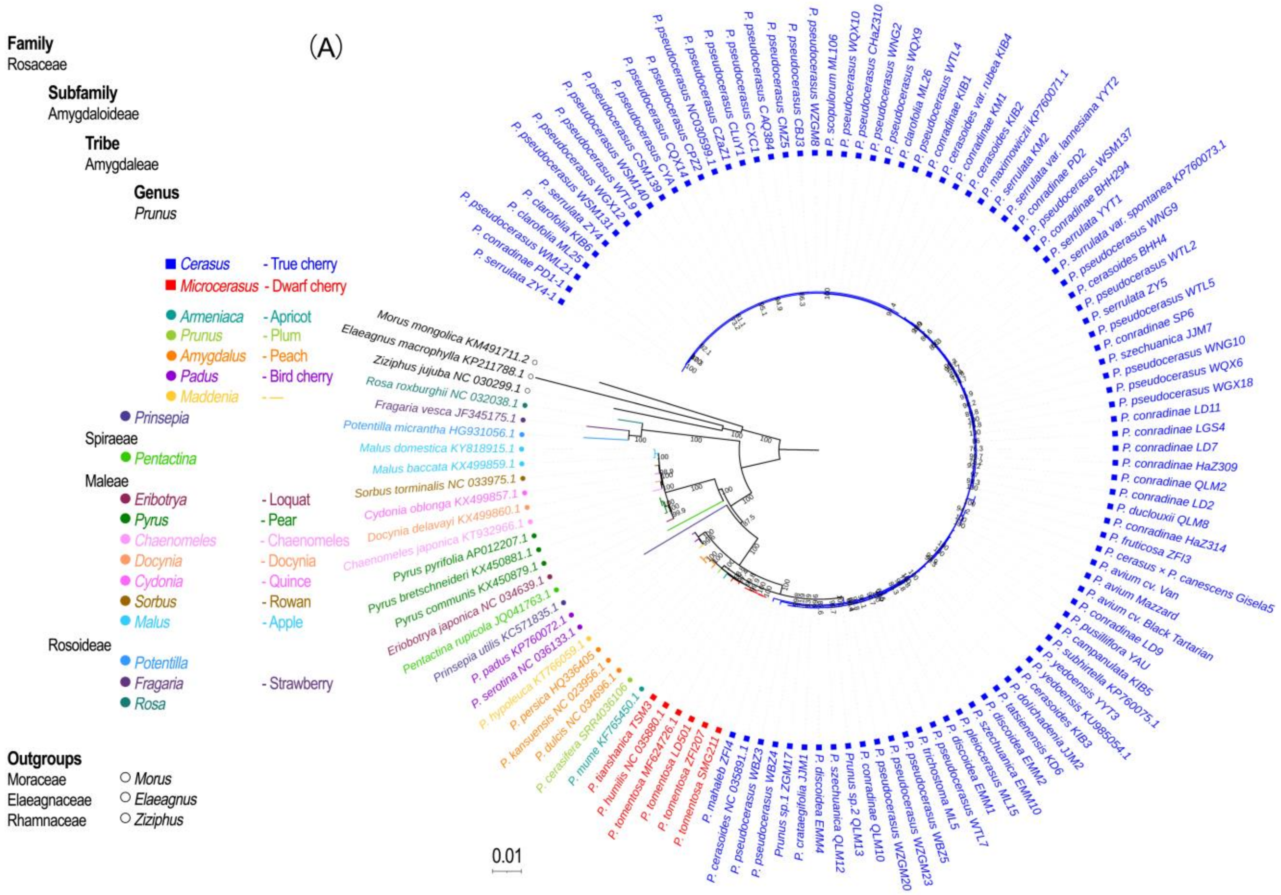

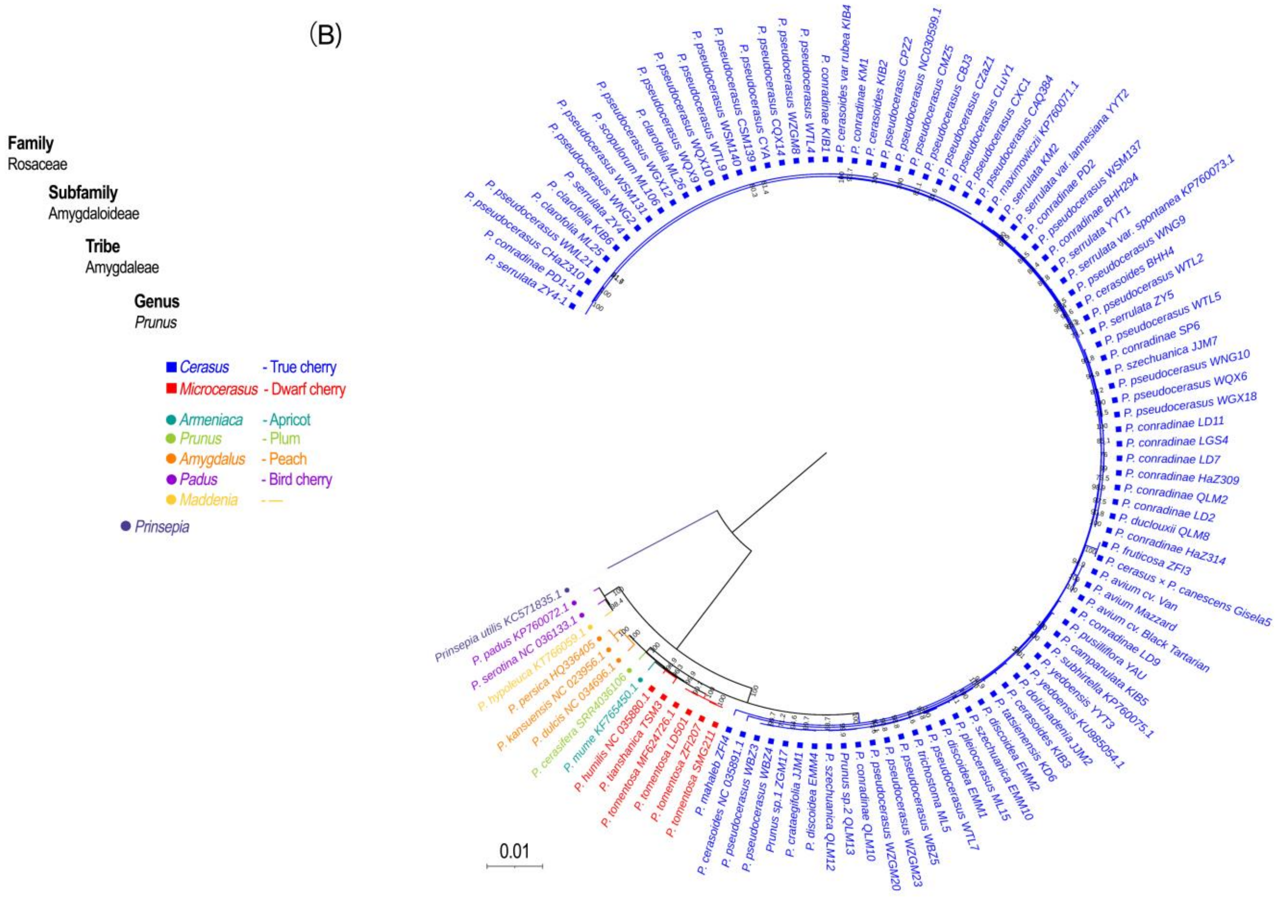

Supplementary Figure 8 Maximum-likelihood phylogenetic trees constructed with WCGD (A) and PCGD (B) datasets. The ML bootstrap values (BS) over 50% are shown on the major clades.

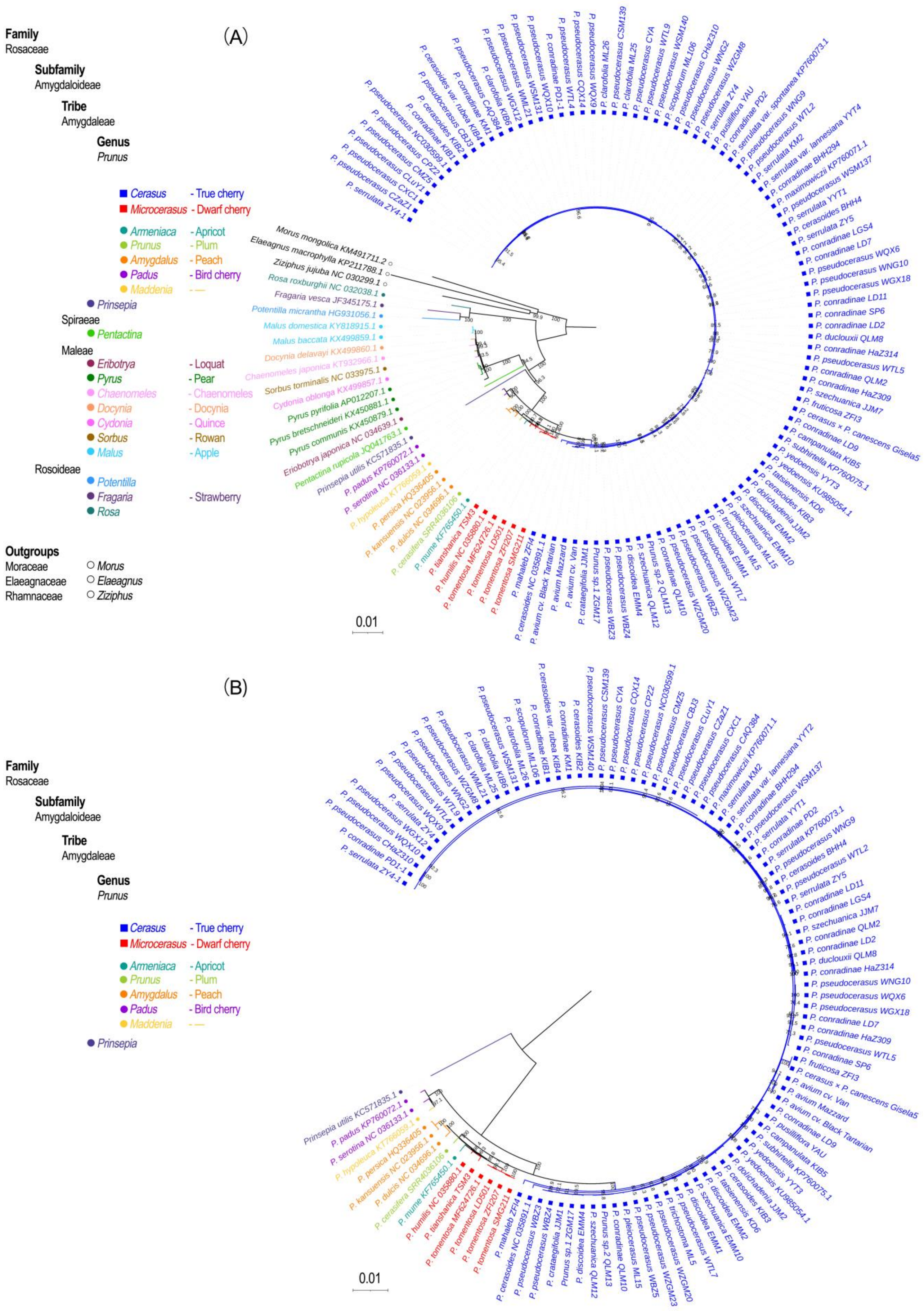

Supplementary Figure 9 Maximum-likelihood phylogenetic trees constructed with WOID (A) and POID (B) datasets. The ML bootstrap values (BS) over 50% are shown on the major clades.

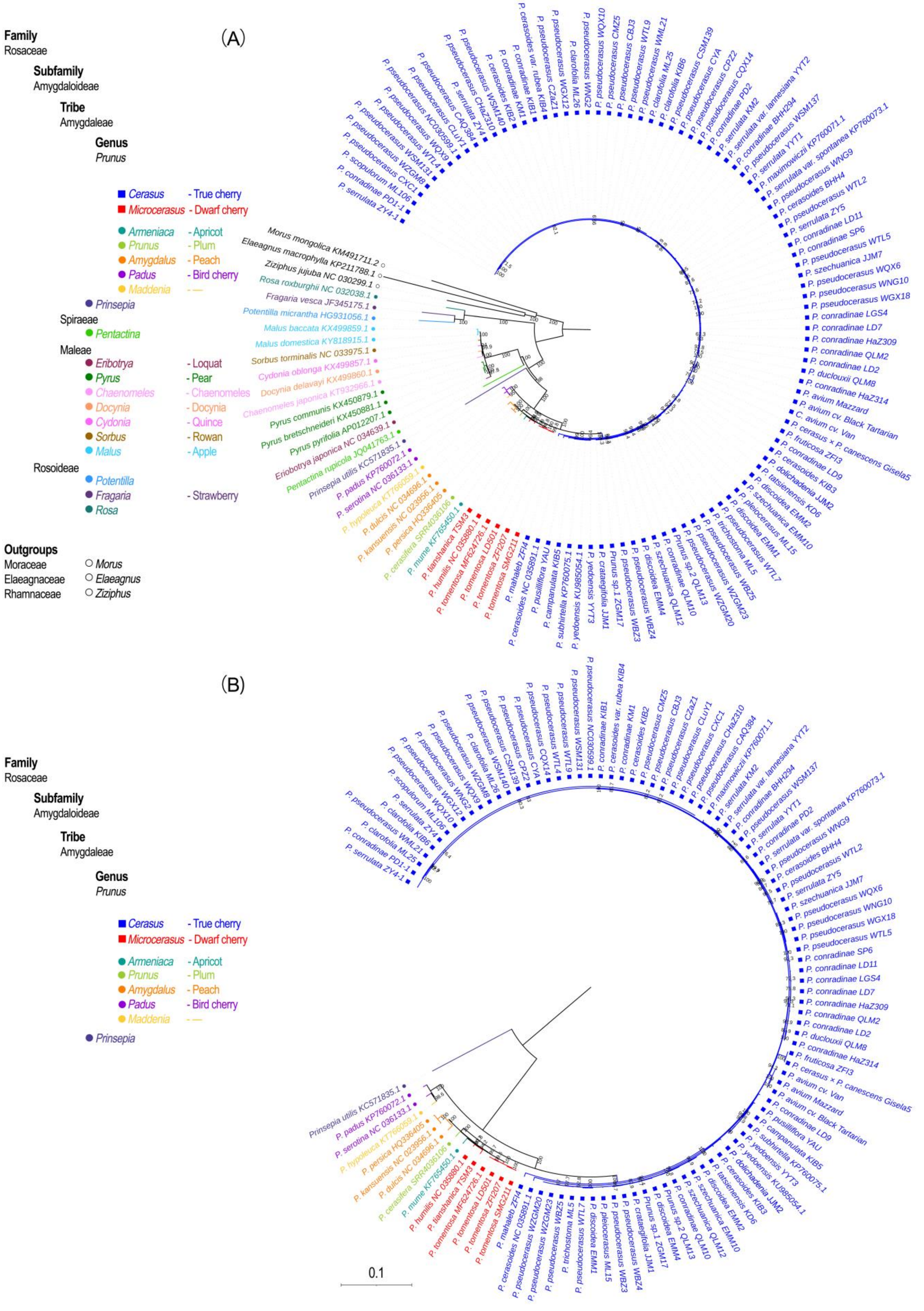

Supplementary Figure 10 Maximum-likelihood phylogenetic trees constructed with VSWD (A) and VSPD (B) datasets. The ML bootstrap values (BS) over 50% are shown on the major clades.

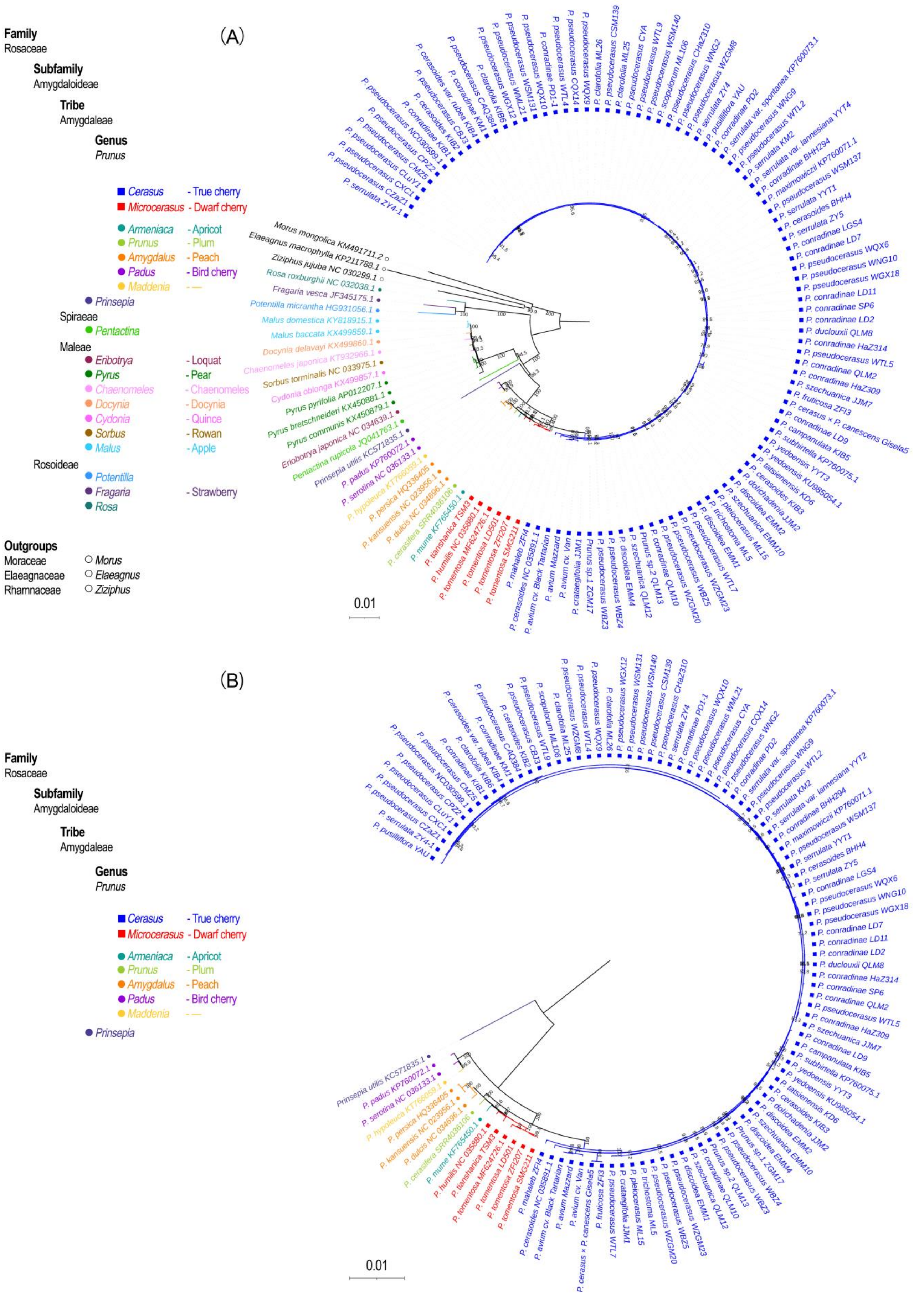

Supplementary Figure 11 Maximum-likelihood phylogenetic trees constructed with WGSD (A) and PGSD (B) datasets. The ML bootstrap values (BS) over 50% are shown on the major clades.

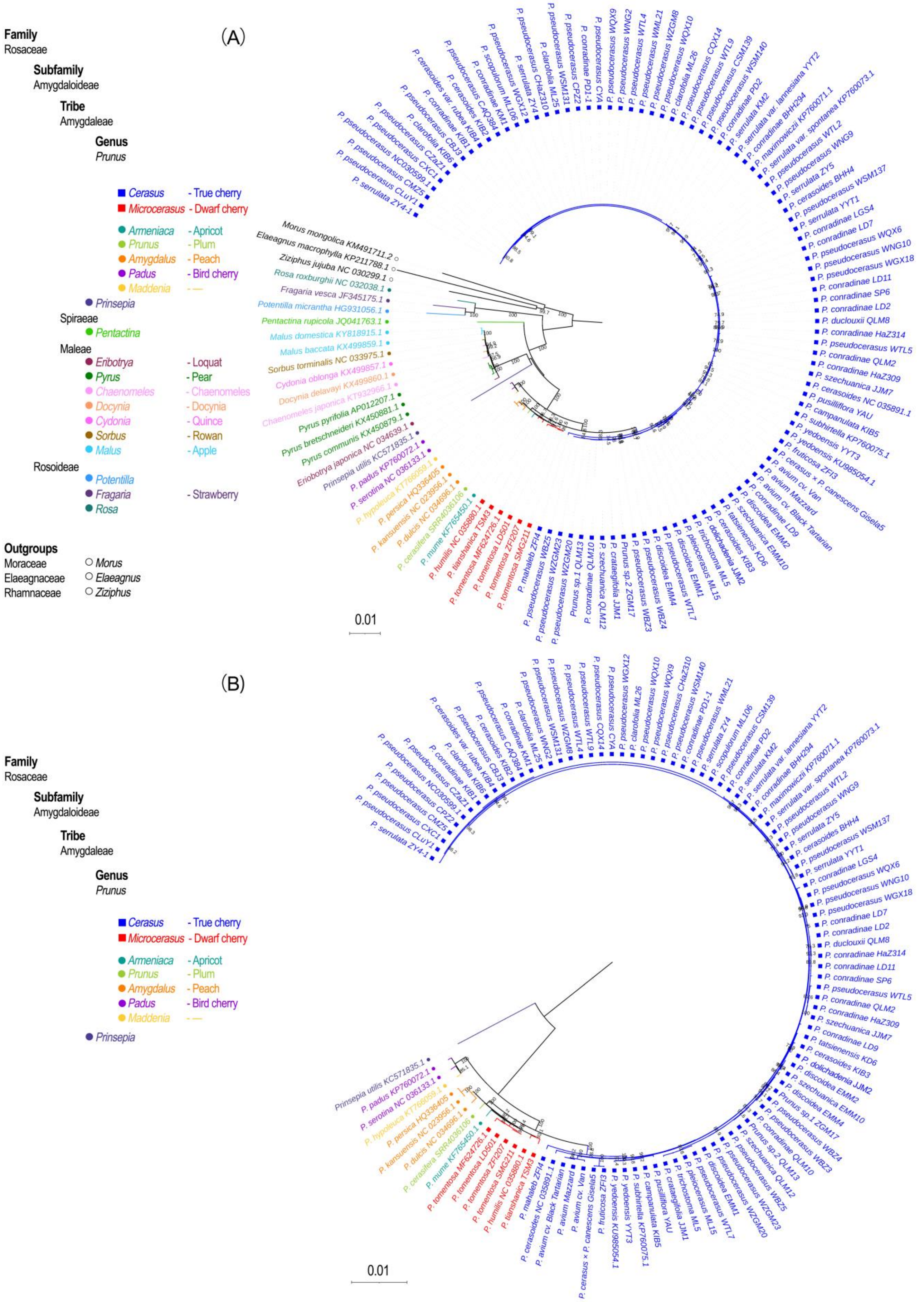

Supplementary Figure 12 Maximum-likelihood phylogenetic trees constructed with PCWD (A) and PCPD (B) datasets. The ML bootstrap values (BS) over 50% are shown on the major clades.

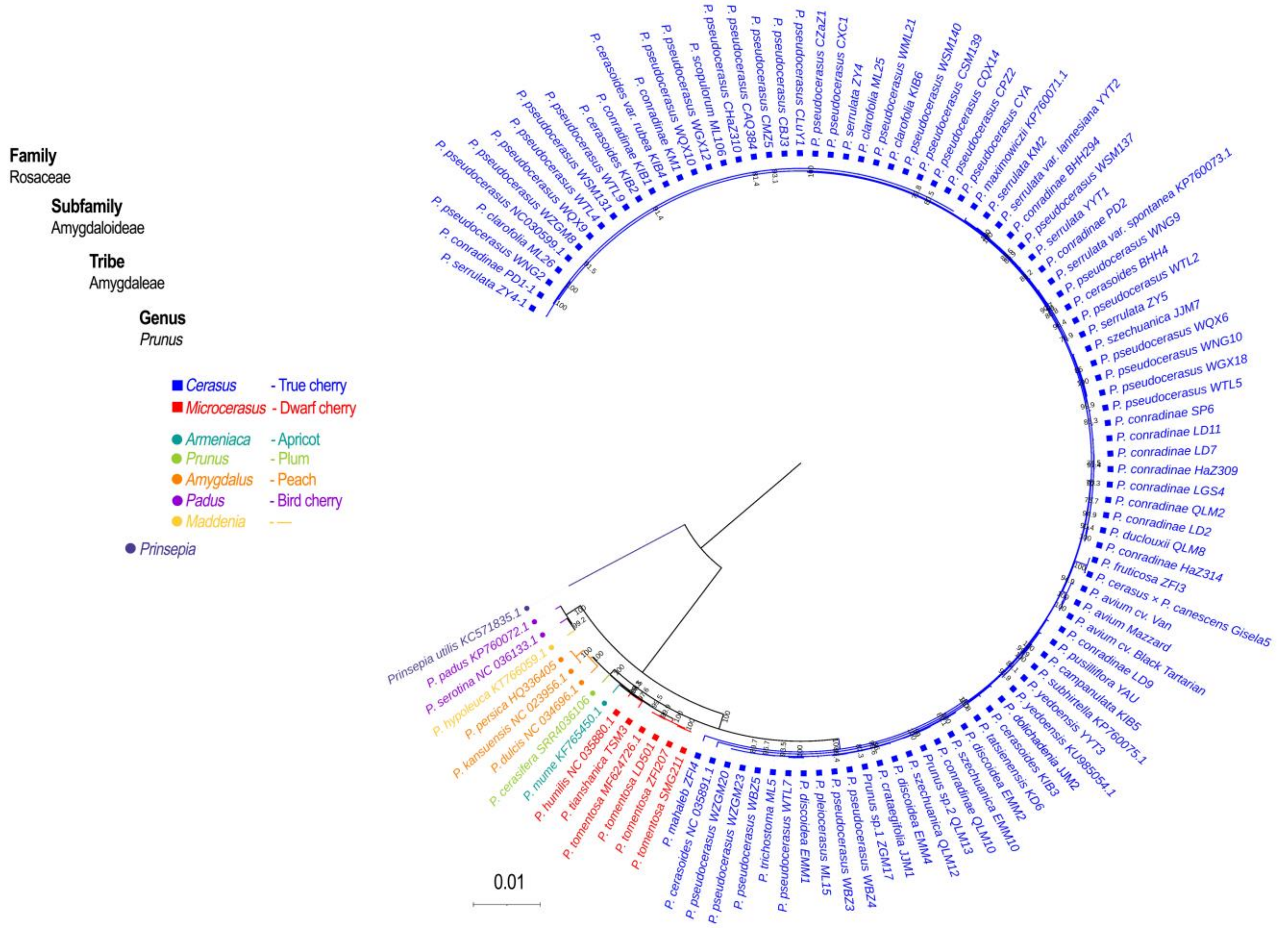

Supplementary Figure 13 Maximum-likelihood phylogenetic tree constructed with PPGD dataset. The ML bootstrap values (BS) over 50% are shown on the major clades.

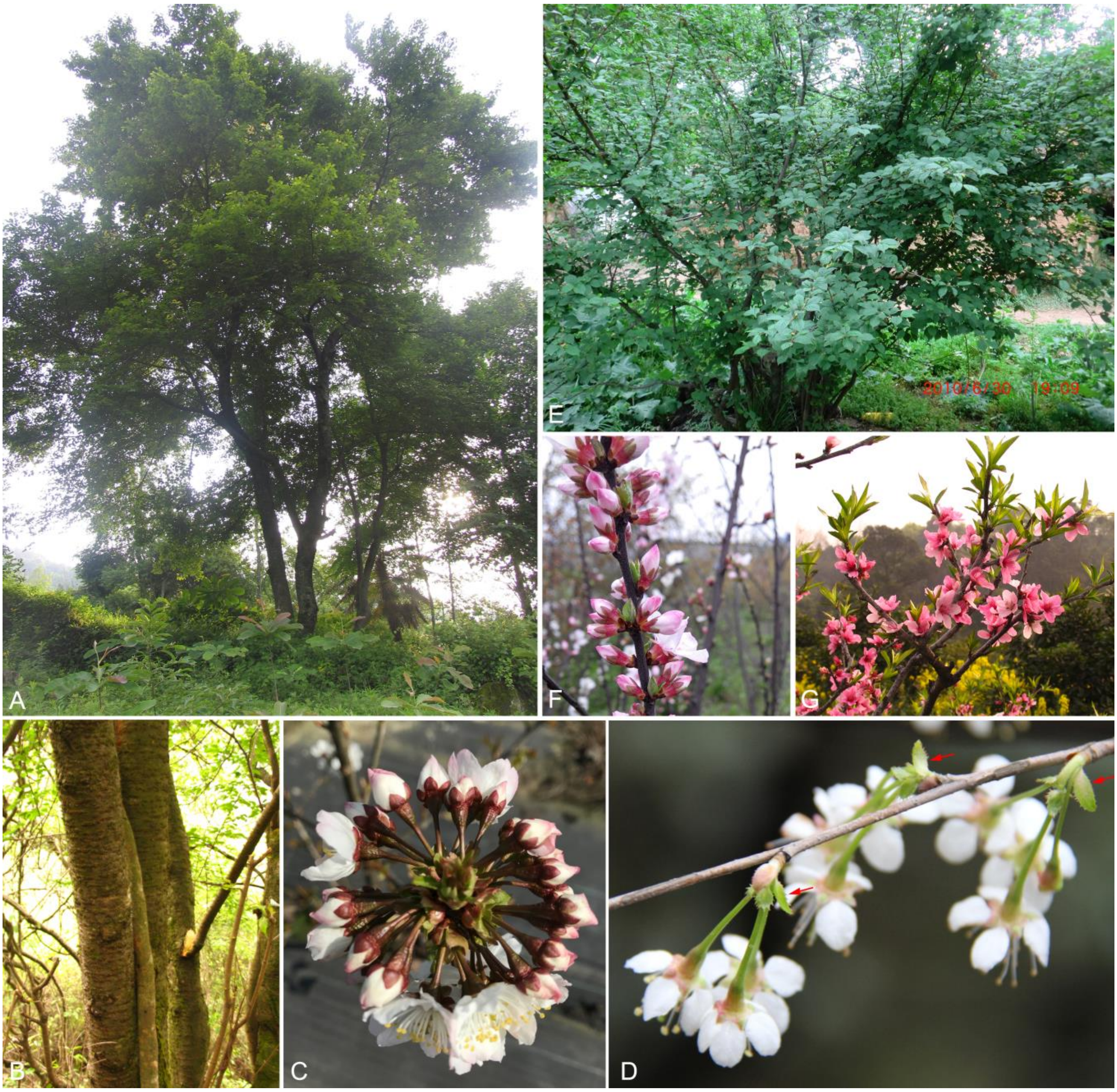

Supplementary Figure 14 The main morphological differences among true cherry (A-D), dwarf cherry (E-F) and close relatives (G). **A**-**C**: *P. pseudocerasus*, **A**: growth habit: tall trees, **B**: bark with lateral lined lenticels, **C**: inflorescences corymbose, **D**: *P. discoidea*, inflorescences umbellate with long pedicel and bract (red arrows) below each flower; **E**-**F**: *P. tomentosa*, **E**: growth habit: shrubs, **F**: flowers 2 in a fascicle with pedicel almost absent; **G**: *Prunus persica*, 1(or 2)-flowered with pedicel nearly absent.

References

Bate-Smith, E.C. (1961). Chromatography and taxonomy in the Rosaceae, with special reference to *Potentilla* and *Prunus*. *Bot. J. Linn. Soc*. 58: 39-54.

Bentham, G., and Hooker, J.D. (1865). *Genera plantarum, vol. 1*. London: L. Reeve & Co. Ltd.

Bortiri, E. et al. (2001). Phylogeny and systematics of *Prunus* (Rosaceae) as determined by sequence analysis of ITS and the chloroplast *trn*L-*trn*F spacer DNA. *Syst. Bot.* 26: 797-807.

Bortiri, E., Heuvel, B.V., and Potter, D. (2006). Phylogenetic analysis of morphology in *Prunus* reveals extensive homoplasy. *Plant Syst. Evol.* 259: 53-71.

Bortiri, E., Oh, S.H., Gao, F.Y., and Potter, D. (2002). The phylogenetic utility of nucleotide sequences of sorbitol 6-phosphate dehydrogenase in *Prunus* (Rosaceae). *Am. J. Bot.* 89: 1697-1708.

Chin, S.W., Shaw, J., Haberle, R., Wen, J., and Potter, D. (2014). Diversification of almonds, peaches, plums and cherries–molecular systematics and biogeographic history of *Prunus* (Rosaceae). *Mol. Phylogenet. Evol.* 76: 34-48.

de Tournefort, J. (1700). *Institutiones Rei Herbariae. 1st ed*. Paris: Typograhia Regia.

Focke, W. O. (1894). Rosaceae. Die natürlichen pflanzenfamilien III. Engler, A., Prantl, K. Engelmann, Leipzig, Germany, 1-61.

Ghora, C., and Panigrahi, G. (1995). *The family Rosaceae in India, vol. 2*. Dehra Dun: Bishen Singh Mahendra Pal Singh.

Hutchinson, J. (1964). *The genera of flowering plants*. Oxford (UK): Clarendon Press.

Ingram, C. (1948). *Ornamental cherries*. London: Country Life Limited.

Koehne, E. (1911). Die Gliederung von *Prunus* subgen. *Padus*. Verhandhunben des botanischen Vereins der Provinz Brandenburg. Berlin., 52, 101-108.

Komarov, L. V. (1971). *Rosaceae: Rosoideae, Amygdaloideae. In Flora of the U.S.S.R, vol. 10, 1-512. English translation*. Washington, D.C.: Smithsonian Institution.

Krüssmann, G. (1978). *Manual of cultivated broad-leaved trees and shrubs. vol. 3*. (Pru-Z. English translation 1986). Portland: Timber Press.

Lee, S., and Wen, J. (2001). A phylogenetic analysis of *Prunus* and the Amygdaloideae (Rosaceae) using ITS sequences of nuclear ribosomal DNA. *Am. J. Bot.* 88: 150-160.

Linnaeus, C. (1754). Genera plantarum, 5th ed. Stockholm: Sweden.

Miller P. (1754). The gardener’s dictionary, 4th ed. London.

Mowrey, B.D., and Werner, D.J. (1990). Phylogenetic relationships among species of *Prunus* as inferred by isozyme markers. *Theor. Appl. Genet.* 80: 129-133.

Potter, D., Still, S.M., Grebenc, T., Ballian, D. Božič, G., Franjiæ, J., et al. (2007). Phylogeny and classification of Rosaceae. *Plant Syst. Evol.* 266: 5-43. doi:10.1007/s00606-007-0544-z.

Rehder, A. (1940). Manual of cultivated trees and shrubs hardy in North America exclusive of the subtropical and warmer temperate regions, 2nd ed. New York: Macmillan.

Schneider, C. (1905). Illustriertes Handbuch der Laubholzkunde. Jena: G. Fischer.

Shi, S., Li, J.L., Sun, J.H., Yu, J., and Zhou, S.L. (2013). Phylogeny and classification of *Prunus sensu lato* (Rosaceae). *J. Integr. Plant Biol.,* 55: 1069-1079. doi:10.1111/jipb.12095

Shishkin, B.K., and Yuzepchuk, S.V. (1971). *Flora of the U.S.S.R. vol.10.* Jerusalem: Israel Program for Scientific Translations Ltd.

Takhtajan, A.L. (1997). Diversity and Classification of Flowering Plants. New York: Columbia University Press.

Yü, D.J., Lu, L. T., Ku, T. C., Li, C.L., and Chen, S.X. (1986). Flora of China, Vol. 38. Beijing: Science Press.
